# Conglomerate Crystallization in the Cambridge Structural Database (2020–2021)

**DOI:** 10.1021/acs.cgd.3c00019

**Published:** 2023-03-22

**Authors:** Mark P. Walsh, James A. Barclay, Callum S. Begg, Jinyi Xuan, Matthew O. Kitching

**Affiliations:** † Process Research and Development, Carbogen Amcis Ltd., 303 Clayton Lane, Manchester, M11 4SX, U.K.; ‡ Department of Chemistry, Durham University, Lower Mount Joy, South Rd., Durham, DH1 3LE, U.K.

## Abstract

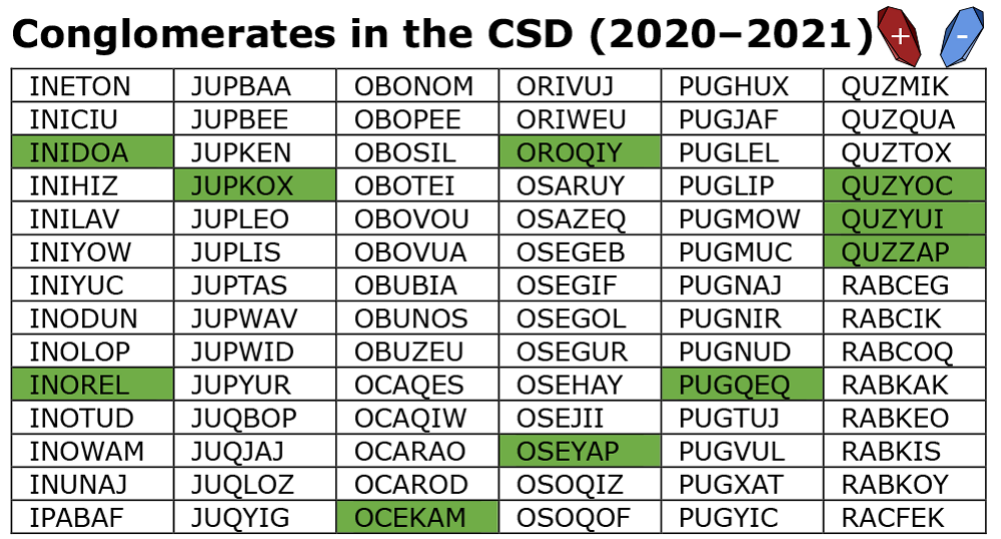

Conglomerate crystals are materials capable of undergoing spontaneous resolution and were responsible for the discovery of molecular chirality. Their relevance to modern chemical and crystallographic sciences has been hindered by the difficulty in identifying and searching materials with this characteristic ability to spontaneously bias their own enantioenrichment. With the release of the November 2021 distribution of the Cambridge Structural Database (CSD) (version 5.43), a fresh quantity of chiral conglomerate crystals is expected to have been published in the CSD without identification. Indeed, no crystals in the CSD have been identified as a spontaneously resolving conglomerate crystal in their crystallographic information file since the 2019 release, despite the deposition of over 108,000 new crystal structures into the database over the same time period. A manual inspection of crystals deposited between 2020 and 2021 was conducted to identify 343 new chiral materials which exhibit conglomerate crystallization behavior. It is hoped that the continued manual curation of this list will aid those in the crystallographic and synthetic communities to study and exploit this spontaneous enantioenrichment behavior.

## Abstract 2

Conglomerate crystals are materials capable of undergoing spontaneous resolution and were responsible for the discovery of molecular chirality. The study of this crystallographic behavior has been hindered by the difficulty in identifying and searching materials with this characteristic ability to spontaneously bias their own enantioenrichment. A manual inspection of crystals deposited within the Cambridge Structural Database between 2020 and 2021 was conducted to identify 343 new chiral materials which exhibit conglomerate crystallization behavior. Several recommendations for how these materials can be identified within crystallographic databases are suggested for the wider crystallographic community to consider.

## Introduction

The Cambridge Structural Database (CSD) is undergoing continued growth and has proven to be an invaluable resource for the study of crystallographic phenomena. Landmark papers which underpin our understanding of broad crystallographic trends often rely on the ability to survey the millions of crystal entries contained within the CSD with the aid of automatic searching tools. Research into topics such as C–H···O, C–H···N, and C–H···Cl hydrogen bonding,^1^ N–H···F, O–H···F hydrogen bonding,^2^ false conglomerates,^3^ kryptoracemates,^4,5^ molecular symmetry assignment,^6^ achiral molecules in noncentrosymmetric space groups,^7^ crystallization of chiral compounds,^8^ hydrogen bond prediction,^9,10^ and high Z′ crystallization^11,12^ have all benefited from such an approach. These efforts have been fruitful because of the high quality crystallographic data and metadata recorded within each individual crystallographic information file (CIF) entry, which have been collected by the crystallographic community and made available by the deposition to a crystallographic database.

However, crystallographic databases such as the CSD cannot currently be used to automatically analyze a fundamental crystallographic phenomenon: spontaneous resolution. A racemic material may spontaneously resolve by crystallizing as conglomerate crystals, that is, the spontaneous formation of individually enantioenriched crystals. The implications of this behavior and how to implement a conglomerate crystallization as a strategy for resolution and asymmetric synthesis have been discussed previously.^13^ This conglomerate crystallization behavior is not being actively tracked within crystallographic databases because it is not standard practice to include the necessary metadata within a CIF. Without the inclusion of the relevant information which would identify a crystal as a conglomerate upon deposition of the CIF to a crystallographic database, chiral conglomerate crystals will be indistinguishable from other enantioenriched crystals which have originated from nonspontaneous asymmetric synthesis or have been isolated from natural sources when searched by automated means.

The identification of conglomerate crystals prior to deposition to a crystallographic database is hindered by the fact that most chiral conglomerate crystals are synthesized and crystallized by synthetic chemists. While synthetic chemists are familiar with Pasteur’s spontaneous resolution of tartrate salts,^14^ they may not be fully aware of the synthetic potential that this behavior presents. For most synthetic chemists, identifying and reporting on crystallographic phenomena are not the focus of their publications. It is not unreasonable for the synthetic chemist to assume that a racemic material would also produce racemic crystals. However, in roughly 10% of cases the crystals will exhibit conglomerate behavior, and therefore each individual crystal will no longer be racemic. While it is not the responsibility of the synthetic community to record and discuss crystallographic behaviors, the identification of conglomerate crystallization requires the cooperation between the synthetic and crystallographic communities. More effective communication between these traditionally separate fields would be to their mutual benefit.

Previous work on cataloging the phenomenon of conglomerate crystallization was initially conducted by Jacques, Colet, and Wilen in their definitive book.^15^ Our group recently conducted a manual search of the CSD to unearth previously unreported instances of chiral molecules undergoing conglomerate crystallization, encompassing crystals deposited to the CSD from 1963–2019 as well as literature sources.^13^ Since the completion of that search, updated distributions of the CSD have been released containing approximately 108,000 new crystal entries by the end of 2021 (as per version 5.43; November 2021). Since no means to automatically record and search conglomerate crystallizations are currently available, we sought to continue to manually catalogue this important crystallographic behavior. By continuing this identification of chiral conglomerate crystals we hope to aid the crystallographic community in understanding this special crystallographic behavior and allow the synthetic community to exploit these substrates in their pursuits to obtain enantioenriched materials via preferential crystallization and spontaneous deracemization.

### Application in Asymmetric Synthesis

Despite the lack of means to search for conglomerate behaviors in crystallographic databases, there is a continued interest in developing spontaneous deracemization protocols for asymmetric synthesis,^16^ which is evident by the number of high quality publications being published on this topic between 2020 and 2021.^17−26^ Often the method for finding a new a substrate capable of conglomerate crystallization is by brute force—the chemist(s) will synthesize a small library of a core scaffold in a racemic fashion and subsequently grow and analyze the crystals of each substrate. In some cases, multiple substrates with similar substitutions can exhibit conglomerate behavior.^23^

Exploiting crystallization as a means to achieve asymmetric synthesis is not typically considered by synthetic chemists. Recent reports of crystallization-driven enantioselective syntheses have demonstrated impressive stereocontrol in their products.^27,28^ Protocols which combine a cocrystallization event with solution phase racemization also demonstrate the viability of combining synthetic transformations with crystallization in producing enantioenriched materials.^29−31^ While these protocols are not classed as spontaneous deracemizations, since they employ an enantioenriched agent to bias the diastereomeric relationship in the crystal, they highlight the possibility of engineering a conglomerate via cocrystallization and racemizing the desired stereocenter(s) in order to achieve a spontaneous deracemization. Excitingly, a recent report of cocrystallization of two racemic compounds to form a stable conglomerate system allowed for the preferential crystallization and resolution of both compounds.^32^ The possibility of combining an engineered cocrystallization conglomerate system with simultaneous racemization to access spontaneously enantioenriched materials should be an exciting prospect for those in the field of asymmetric synthesis.

Although achieving spontaneous chiral symmetry breaking and amplification of chiral information is an enticing proposal, the major bottleneck for the widespread uptake of this strategy by synthetic chemists has been the inability for the synthetic chemist to know what substrates will be suitable for this process. Identifying a material which is capable of conglomerate crystallization behavior is the step which is the least predictable and most difficult to control. With the continued manual curation of a list of chiral materials capable of crystallizing in this manner, synthetic chemists will be able to survey the potential substrates and then focus on developing racemization conditions in order to enable spontaneous deracemization of the substrate(s).

## Results and Discussion

### Searching the CSD

The CSD version 5.43 (November 2021) distribution was used to conduct the search for chiral conglomerates published between 2020 and 2021. Search queries were generated using the CCDC software *Conquest*, with parameters chosen to try and minimize the total number of crystals to be manually checked while also maximizing the potential number of chiral conglomerate candidates. Crystals *MUST* exist in Sohncke space group *ANDZ*′ = 1 *AND* were published between 2020 and 2021. The crystals *MUST* be organic, no polymers, single crystal only, *R*_1_ < 0.075, with no errors, and allowing for disorder and salts. Crystals which were published solely as a *CSD Communications* had to be excluded as the synthetic routes for the crystallized materials could not be interrogated. The crystal entries must *NOT* be in carbohydrate, steroid, peptide, or nucleoside/nucleotide classes as these could be enantioenriched by natural sources. In order to minimize the retrieval of achiral molecules from the CSD, all crystal entries *MUST* also contain a carbon center with C(Nonmetal)_4_*OR* H–C(Nonmetal)_3_. Even with this constraint, the search would still contain achiral molecules which crystallized as conglomerates; however, we were only concerned with conglomerates which contained a stereogenic element which would be recognized by a synthetic chemist (i.e., a stereogenic element which may exist in the solution phase). From our search, 898 achiral conglomerate crystals were identified in this search list (available in the Supporting Information).

It was also found that specific strings of text could be used to exclude certain natural products, including “isolated”, “sourced from”, “extracted”, “bark”, “marine”, “sponge”, and “penicillium”. These combined queries created within *Conquest* generated a list of 5968 crystals as potential conglomerates. Natural products could be further filtered when sorting the resulting CSD hits by their structure names; generic naming such as “d-(+)-xylose”, “crokonoid B”, and “wortmannolol” could be excluded due to their natural sources or as targets for asymmetric total syntheses. Compounds listed with known stereochemical assignments could also be excluded from the search. Compound names containing the following stereochemical notation: (+)-, (*−*)-, d-, l-, (*R*)-, and (*S*)-, were removed from the search as these were either sourced from the natural chiral pool or were produced from enantioselective synthetic methodologies and XRD was used for absolute configurational assignment. *This produced a list of 5465 crystal entries in the CSD which were interrogated manually*. From the manual interpretation of the synthetic routes described to produce each reported crystal, a total of 343 chiral conglomerate crystals were identified to have been published in the CSD between 2020 and 2021. The full list of chiral conglomerate structures with their associated Refcodes, molecular structures, and references are available in the Supporting Information.

### Publishing Trends

**Figure 1:**
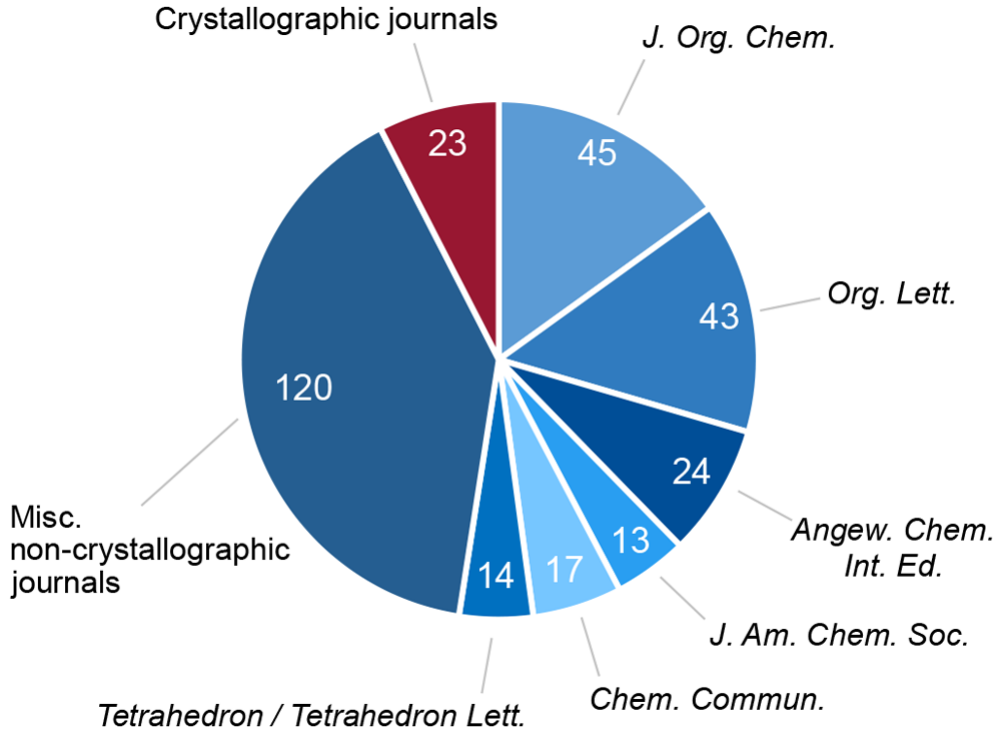
Publication trends of chiral conglomerate crystals (2020–2021).

The trend noted in our previous study^13^—that synthetic chemists are the primary generators of chiral conglomerate crystals within the CSD—has only strengthened between 2020 and 2021. Only 7.5% of chiral conglomerate crystals discovered in the CSD between 2020 and 2021 were published in crystallographic focused journals, as displayed in Figure 1. Chiral conglomerate crystallization is prevalent in noncrystallographic journals, and it remains mostly hidden. Only 38 of the identified conglomerate crystals mentioned the conglomerate behavior in the main text of the paper—most of these instances are groups which pursue the use of conglomerate crystallization for spontaneous deracemization protocols. Of these 38 crystals which have their conglomerate behavior identified within their associated manuscript, none have their conglomerate behavior identified within their CIF using text comments or *CIF Dictionary* approved fields. For example, values of “_chemical_enantioexcess_bulk = 0” and “_chemical_enantioexcess_crystal = 1” would denote an enantiopure crystal arising from racemic bulk material, designating the crystal to be a conglomerate. A potential reason for this omission is the necessary experimental burden to accurately provide these values. The “_chemical_enantioexcess_*” values should be entered only when a suitable technique is described for measuring enantiopurity of a sample in the “_chemical_enantioexcess_*_technique” fields. The experimentalist should employ a valid technique to quantify the enantiopurity of the bulk material, and the crystal used during the diffraction study before such values are described in a CIF. In fact, despite the increasing numbers of crystals deposited every year, no single crystal published in the CSD between 2020 and 2021 was identified as a conglomerate crystal within the deposited CIF. Searching text strings such as “conglomerate” and “spontaneous resolution” within *Conquest* yielded no hits during this time span, highlighting that this crystallographic behavior requires the intervention of the crystallographic community in order for it to be recorded routinely during deposition to a database.

### Space Group Frequency

The frequency of the Sohncke space groups of the conglomerate crystallizations reported in 2020–2021 could be compared to both the previously unearthed conglomerate crystallizations^13^ and the overall frequency of Sohncke space groups for enantioenriched species in the CSD.^8^ Unsurprisingly, the overall distribution of the space groups of the current cohort of chiral conglomerate crystals reported in this work matches those of our previous conglomerate search and the distribution of Sohncke space groups in the CSD. However, there is a over-representation of the *P*2_1_2_1_2_1_ space group in both conglomerate crystallization data sets (1963–2019: 65%; 2020–2021: 63%) versus that present for all enantioenriched chiral species in the CSD (52%). It is also noted that there is an under-representation of the *P*2_1_ space group for the conglomerate crystal data sets (1963–2019: 27%; 2020–2021: 28%) versus the CSD data set (34%). The reason for this discrepancy is unclear, but may hold a fundamental insight into the nature of conglomerate crystallizations. However, due to the high abundance of crystals appearing within these two space groups, this observation will not aid in predicting or searching conglomerate crystallization behaviors.

**Figure 2:**
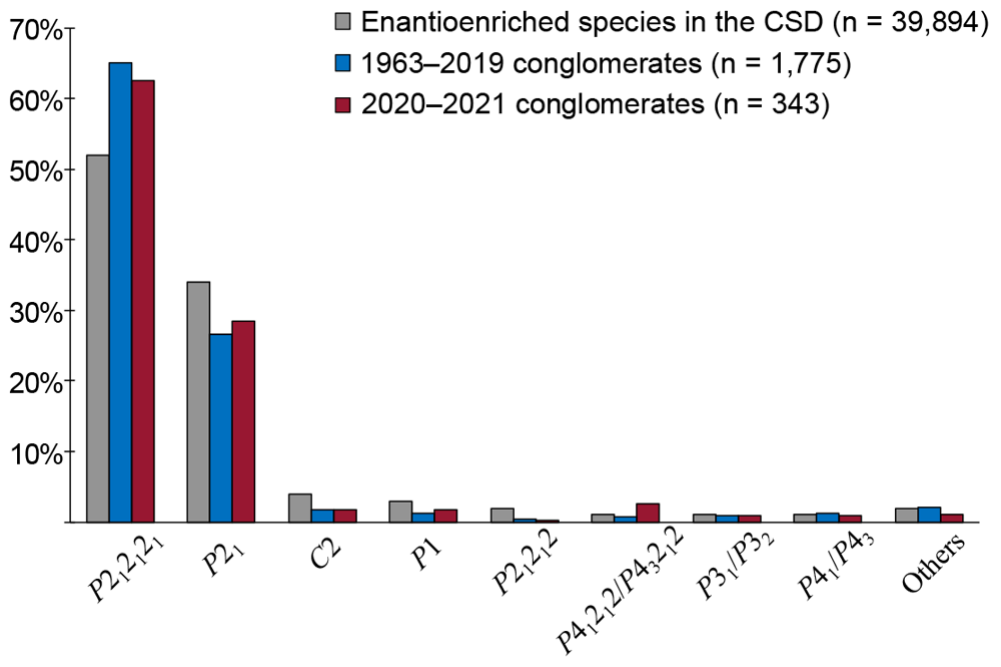
A comparison of the distribution of Sohncke space groups for enantioenriched chiral molecules held within the CSD (gray), our previous chiral conglomerate search (blue), and the chiral conglomerates found in this work (red).

### Conglomerates of Interest

**Figure 3:**
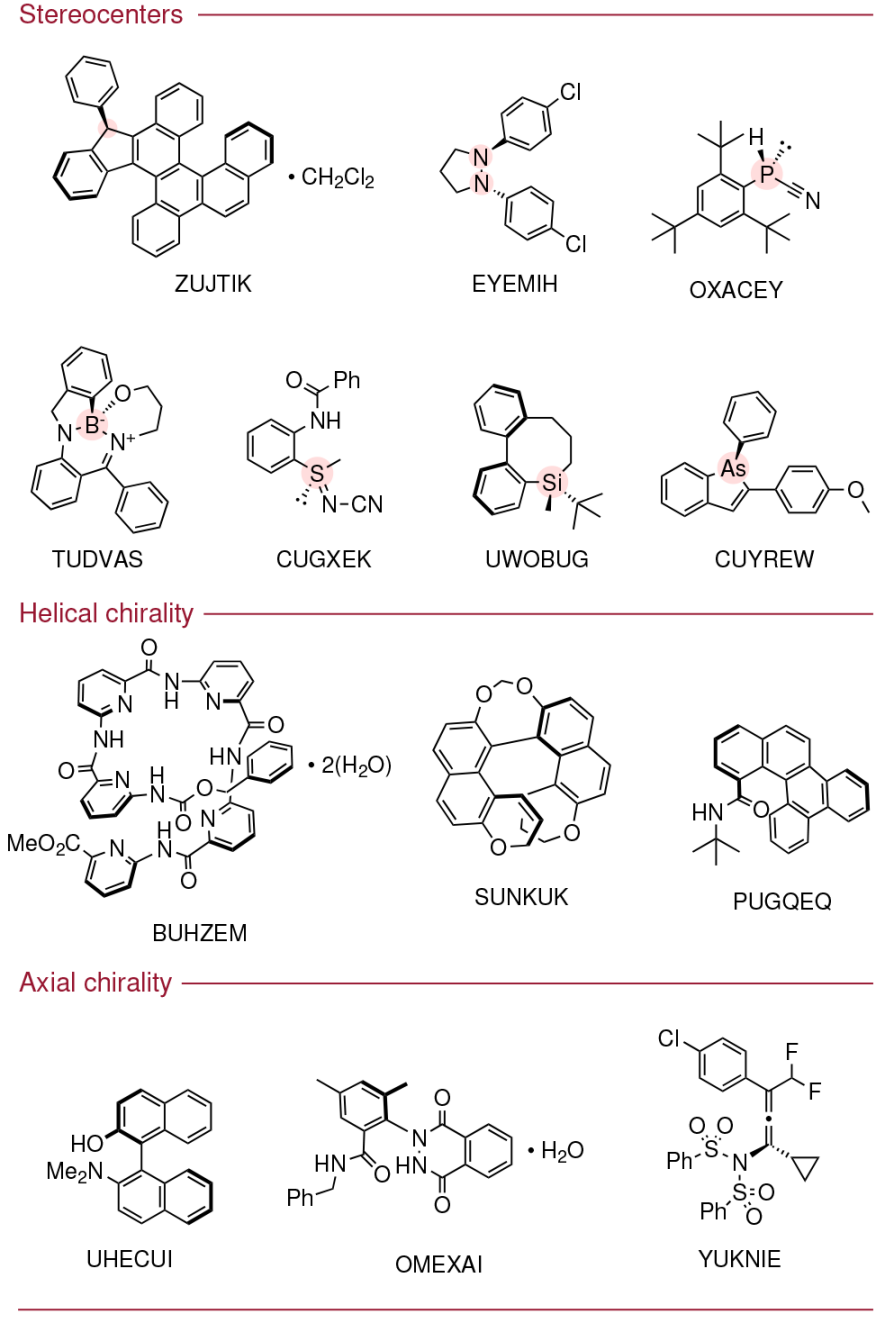
Examples of the stereogenic elements resolved by conglomerate crystallization. The identified conglomerate structures are labeled with their associated CSD Refcodes: ZUJTIK,^33^ EYEMIH,^34^ OXACEY,^35^ TUDVAS,^36^ CUGXEK,^37^ UWOBUG,^38^ CUYREW,^39^ BUHZEM,^40^ SUNKUK,^41^ PUGQEQ,^42^ UHECUI,^43^ OMEXAI,^44^ and YUKNIE.^45^

The structural diversity of materials that undergo conglomerate crystallization is upheld in the set of chiral conglomerates presented in this search. The full list of chiral conglomerate crystals with their chemical structures, associated Refcodes, and literature references are available in the Supporting Information. Figure 3 highlights a small subset of the total list to display the diversity of the materials capable of conglomerate crystallization. Carbon, nitrogen, phosphorus, boron, sulfur, silicon, and arsenic based stereocenters are among the point stereogenic elements which have been resolved through this behavior. However, most stereocenters are unsurprisingly carbon-based, due to their prevalence in organic synthesis.

Stereocenters are not the only stereogenic elements observed within conglomerate crystallization. Helical based chirality is also possible to enantioenrich through crystallization. While helical based conglomerate crystals grown from achiral monomers through impressive enantioselective supramolecular assembly in their crystal structures have been reported,^46,47^ we wish to only highlight structures which may preserve their enantioenrichment upon dissolution of the crystal. Axial based chirality has also been present within chiral conglomerate crystallization, with atropisomeric scaffolds (UHECUI^43^ and OMEXAI^44^) and even unsymmetrical allene systems (YUKNIE^45^) being reported.

**Figure 4:**
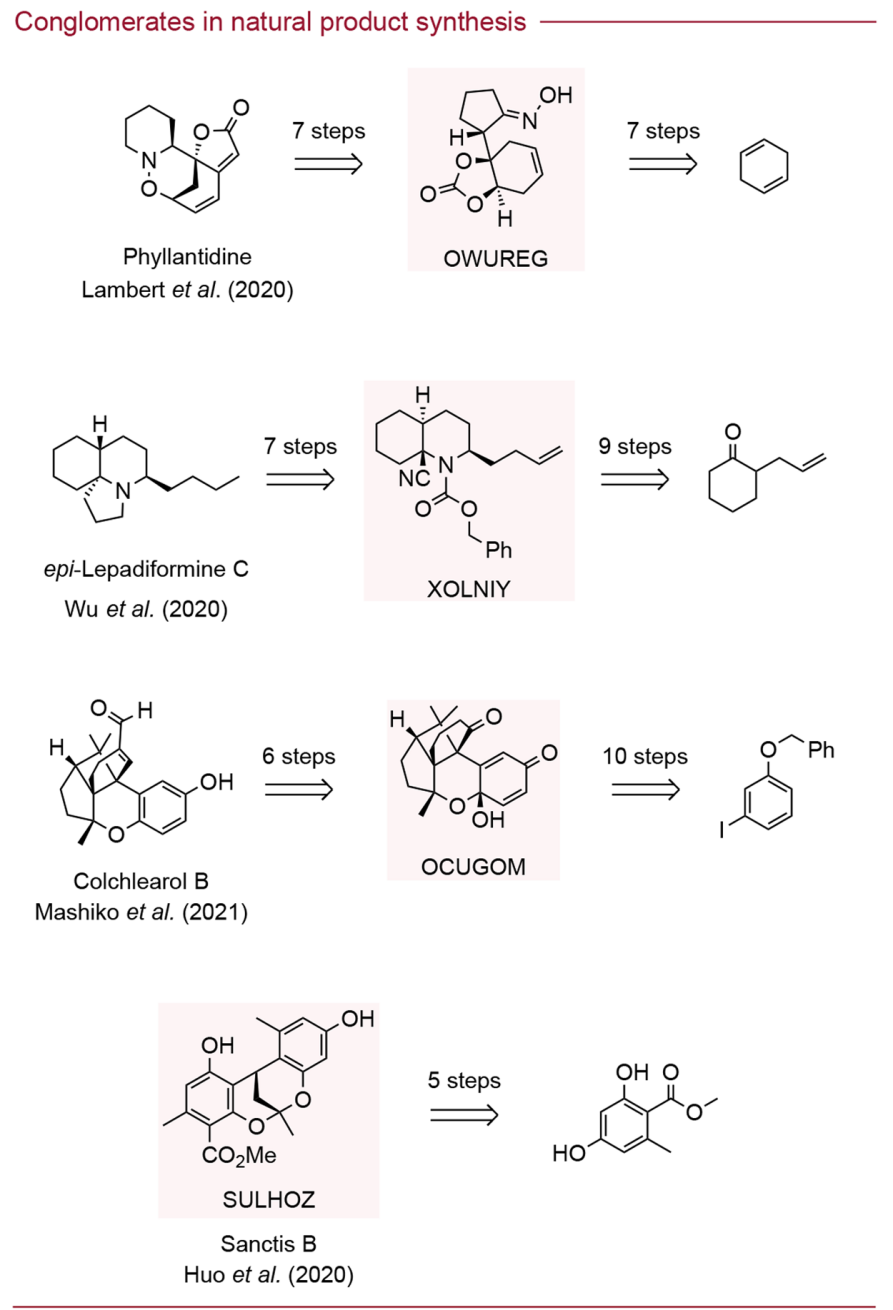
Natural product syntheses with conglomerate crystals identified in the published synthetic routes. The identified conglomerate structures are labeled with their associated CSD Refcodes: OWUREG,^48^ XOLNIY,^49^ OCUGOM,^50^ and SULHOZ.^51^

Further examples of conglomerate crystallization were observed to have occurred within the synthesis of natural products in this updated distribution of the CSD, as shown in Figure 4 (OWUREG,^48^ XOLNIY,^49^ and OCUGOM^50^). Another example of a natural product structure, Sanctis B (SULHOZ^51^), was noted to have crystallized as a conglomerate crystal. Admittedly, these structures identified as conglomerate crystals would be too complex to undergo spontaneous deracemization under the current state-of-the-art racemization protocols. However, the use of preferential crystallization could allow for bulk resolution of these substrates. Furthermore, the possibility that synthetic chemists could design new racemization protocols for simpler conglomerate substrates would, through spontaneous deracemization, allow enantioselective synthesis of natural products without input from the natural chiral pool.

### Engineered Conglomerate Crystals

**Figure 5:**
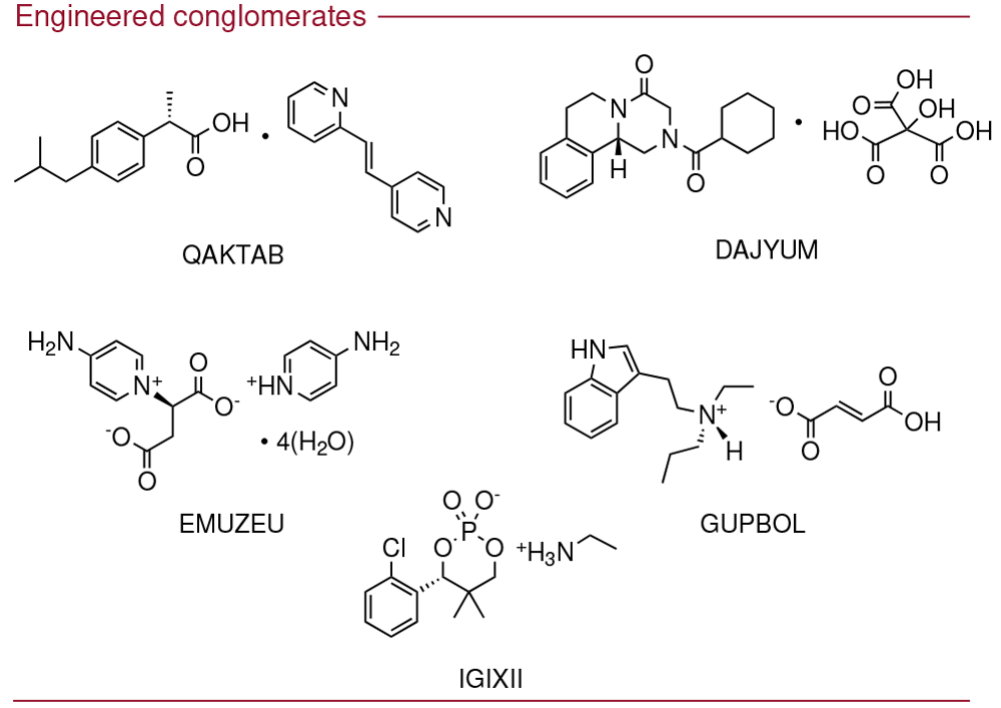
Chiral conglomerates crystals formed by crystal engineering. The conglomerate structures are labeled with their associated CSD Refcodes: QAKTAB,^52^ DAJYUM,^53^ EMUZEU,^54^ GUPBOL,^55^ IGIXII.^56^

More examples of the use of crystal engineering in order to produce conglomerate crystallizations have been noted and are presented in Figure 5. In two cases (QAKTAB^52^ and DAJYUM^53^), a cocrystallization strategy was employed. In the remaining three examples (EMUZEU,^54^ GUPBOL,^55^ and IGIXII^56^) the substrates were transformed into an organic salt. Notably, all examples presented in Figure 5 use an achiral agent to form the required cocrystal or salt in the engineered crystal. Further study into the engineering of conglomerate crystallizations would enable more control of which target substrates can undergo this type of crystallization behavior, without being at the mercy of the current probabilistic nature of this phenomenon.

The second instance of conglomerate polymorphism (OSUNEY/*P*6_1_ and OSUNEY01/*P*2_1_) was painstakingly observed and isolated from precise melting experiments.^57^ Such examples where multiple polymorphic structures exhibit conglomerate behaviors are exceptionally rare and are ideal case studies for the study of conglomerate crystallization.

### Proposed Applications

The most exciting potential of discovering a conglomerate crystallization from the point of view of a synthetic chemist is the possibility of combining it with racemization conditions in order to facilitate a spontaneous deracemization of the bulk material. It is hoped that once armed with this list of potential substrates, synthetic chemists can begin to hypothesize which conglomerate substrates can be paired with known racemization conditions. Overcoming the lack of documentation of this crystallization phenomenon will lower the barrier to entry for synthetic chemists to develop new protocols for this form of chiral amplification in their syntheses.

### Future Reporting

The presence of unidentified conglomerates within the CSD is symptomatic of the lack of communication between two traditionally separate disciplines—namely the synthetic and the structural communities. To take full advantage of this phenomenon, information transfer between these groups needs to be facilitated. Nowhere can this difference be seen more clearly than when considering *CSD Communications* as a publication avenue. The fastest growing component in the CSD is the use of *CSD Communications* for publication of individual crystal structures.^58^ In 2021, 5,110 structures were published as *CSD Communications*, making it the top journal in the CSD, with 9.3% of the total structures published that year. For comparison, 1,645 structures were published in the CSD with *Cryst. Growth Des.* and 1,530 structures with *CrystEngComm*. The publishing mechanism offered by *CSD Communications* achieves its admirable aim of the rapid communication of crystal structures. However, there is no consensus on how to identify conglomerate behavior within a CIF, and publishing a crystal structure within *CSD Communications* alone does not allow authors to report the synthetic route of the material, thereby obscuring the identification of conglomerate behaviors by manual inspection. This has been the most accessible method of publishing a lone crystal structure for a synthetic chemist, but ultimately it is both the synthetic and the crystallographic communities that suffer from the loss of information by not identifying the conglomerate behavior within the CIF or not reporting a synthetic protocol for the material. As such, the optimal solution is to capture the conglomerate crystallization behavior during the deposition process to a crystallographic database. This might be achieved by prompting the user to consider if the chiral material had originated from a racemic process by means of a checkbox and creating a searchable identifier associated with the deposited CIF if the conditions for a conglomerate crystal are met. This has the advantage of capturing conglomerate behavior without requiring the user to be familiar with the crystallographic terminology. Without the intervention of the crystallographic databases, the crystallographic community could adopt the inclusion of a searchable identifier within the CIF prior to deposition to a crystallographic database either by including a text string or by utilizing the “_chemical_enantioexcess_*” CIF fields in order to allow for the identification and automatic searching of conglomerate crystallization. It will be the wider crystallographic community which ultimately decides on the standard practice to record this metadata.

## Conclusion

By conducting a manual search of the distribution of the CSD version 5.43 (November 2021) an additional 343 chiral conglomerate crystallizations have been identified to have been published between 2020 and 2021. This list is presented in full within the Supporting Information. Trends in the journals that contained chiral conglomerate crystallizations reinforced the previous observation that the majority of examples of this behavior appear in noncrystallographic journals. By manually curating the structures which are capable of undergoing this form of crystallization, substrates which may be paired with racemization conditions can be identified by synthetic chemists in order to mediate new spontaneous deracemization protocols.

## Data Availability

The annotated output from *Conquest* with the classification for each crystal (.xlsx) and the chiral conglomerate crystals identified in this work have been collated as CIF and Refcode list formats (.cif, .txt, .gcd) and are freely available from the *Zenodo* data repository (10.5281/zenodo.7473978).

## Supporting Information Available

The full curated list of chiral conglomerate crystals published between 2020–2021, along with their chemical structures and their associated reference (PDF)

## Abbreviations

CCDC: Cambridge Crystallograpic Data Center
CIF: Crystallographic Information File
CSD: Cambridge Structural Database
IUCr: International Union of Crystallography
XRD: X-ray diffraction.
