# Supplementary material for: Conglomerate Crystallization in the Cambridge Structural Database (2020–2021)
Source: Cryst Growth Des. 2023 Mar 22;23(4):2837–44. doi: 10.1021/acs.cgd.3c00019 (PMC10080650; doi:10.1021/acs.cgd.3c00019)
Supplement: Supplementary file 1 — cg3c00019_si_001.pdf [file cg3c00019_si_001.pdf]

Supporting Information

Conglomerate Crystallization in the Cambridge Structural Database (2020–2021)

Mark P. Walsh[a*], James A. Barclay[b†], Callum S. Begg[b†], Jinyi Xuan[b†], Matthew O. Kitching[b*]

[a] Process Research and Development, Carbogen Amcis Ltd., 303 Clayton Lane, Manchester, M11 4SX, United Kingdom.

[b] Department of Chemistry, Durham University, Lower Mount Joy, South Rd., Durham, DH1 3LE, United Kingdom.

[†] These authors contributed equally to this work

* Correspondence to be sent to:

markpwalsh1@gmail.com

matthew.o.kitching@durham.ac.uk

Contents

Search parameters.. 3

Conglomerate crystals found within the CSD (2020-2021)... 4

Conglomerate crystals which undergo racemic twinning... 20

References... 21

Search parameters

CSD version 5.43 (November 2021) was used for the search. Queries were generated using Conquest, with the following queries chosen to try and minimise the total number of crystals to be checked while also maximising the potential number of conglomerate candidates. Crystals must exist in Sohncke space group AND Z' = 1 AND were published between 2020–2021. Must NOT be in carbohydrate, steroid, peptide or nucleoside/nucleotide classes. Must have carbon centre with C(Non-metal)$_4$ OR H-C(Non-metal)$_3$. The main focus was put on carbon stereocentres since they make up 98% of all stereocentres for chiral compounds within in the CSD.

Must be organic, no polymer, single crystal only, $R_1 < 0.075$, no errors, with disorder and salts allowed.

Crystals published solely as a *CSD Communication* were excluded as the synthetic routes for the crystallised materials could not be interrogated.

It was also found that specific strings of text could be used to exclude certain natural products, including: "isolated", "sourced from", "extracted", "bark", "marine", "sponge", "penicillium". This generated a list of 5,968 crystals as potential conglomerates. Natural products could be further filtered when sorting the resulting CSD hits by their structure names; generic naming such as "D-(+)-xylose", "crokonoid B", "wortmannolol" could be excluded due their natural sources or as targets for asymmetric total syntheses.

Compounds listed with known stereochemical assignments could be excluded from the list too. Compound names with the following: (+), (-), D, L, (R) and (S), were removed from the list as these were either sourced from the natural chiral pool or were produced from enantioselective methodologies and XRD was used for absolute configuration assignment. ***This produced a list of 5,465 crystal entries in the CSD which was sorted manually.***

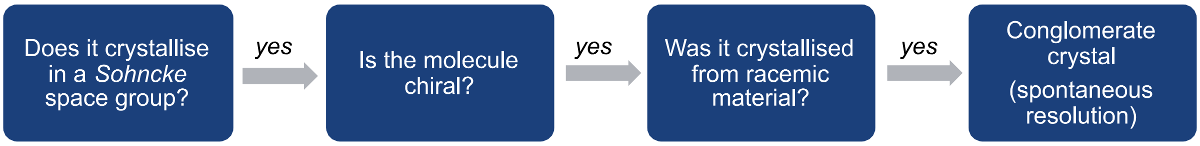

Figure 1 - Flowchart for conditions in the manual identification of a chiral conglomerate crystallisation

Conglomerate crystals found within the CSD (2020-2021)

CSD Code	Space Group	a / Å	b / Å	c / Å	α / °	β / °	γ / °	Publication Year	Reference
**ABULES**	$C222_1$	22.6932	23.0691	35.8716	90	90	90	2021	[1]
**AHIWAS**	$P2_1$	9.8666	9.9949	11.3565	90	114.722	90	2020	[2]
**AHURAZ**	$P2_12_12_1$	5.3984	14.114	20.889	90	90	90	2020	[3]
**AJAQIO**	$P2_12_12_1$	7.3708	10.0629	25.85	90	90	90	2020	[4]
**AJONEV**	$P2_12_12_1$	6.0964	10.1676	19.2632	90	90	90	2020	[5]
**AJOPAT**	$C2$	17.076	9.7625	18.847	90	110.108	90	2020	[5]
**AJUHEV**	$P2_1$	10.5119	17.5916	10.5948	90	94.564	90	2021	[6]
**AJUHIZ**	$P2_1$	10.0405	17.6036	10.7504	90	94.678	90	2021	[7]
**AKEGOP**	$P2_12_12_1$	10.1353	16.1731	21.119	90	90	90	2021	[8]
**AKIJOW**	$P2_1$	7.589	9.6	13.7953	90	92.435	90	2021	[9]
**AMESUJ**	$P2_1$	8.0758	10.5523	12.7168	90	106.603	90	2021	[10]
**ANETUL**	$P2_1$	9.9631	9.1494	13.3605	90	102.001	90	2021	[11]
**ANUVEN**	$P2_12_12_1$	6.0967	11.8625	18.101	90	90	90	2021	[12]
**APOHOF**	$P2_12_12_1$	4.513	13.9662	18.0206	90	90	90	2021	[13]
**APUGOK**	$P2_1$	8.1686	13.0294	10.9124	90	107.327	90	2021	[14]
**AQETOI**	$P2_1$	9.52	10.397	10.131	90	112.109	90	2021	[15]
**AREGAI**	$P2_12_12_1$	6.9289	13.2425	17.2281	90	90	90	2020	[16]
**AREREX**	$P2_12_12_1$	7.4354	15.9512	17.842	90	90	90	2021	[17]
**ARIDUD**	$P2_12_12_1$	5.87	17.968	18.326	90	90	90	2021	[18]
**ARIHER**	$P2_1$	10.5277	14.804	12.4712	90	113.336	90	2021	[19]
**AROCES**	$P2_12_12_1$	10.279	10.468	16.68	90	90	90	2021	[20]
**AROCIW**	$P2_12_12_1$	9.8562	11.037	17.257	90	90	90	2021	[20]
**ASELOC**	$P1$	8.746	11.327	12.761	98.532	109.362	99.518	2021	[21]
**ASILAS**	$P2_12_12_1$	5.9555	15.031	16.1175	90	90	90	2021	[22]
**ATAROF**	$P2_12_12_1$	9.77468	11.1297	24.2596	90	90	90	2021	[23]
**ATEROJ**	$P2_12_12_1$	8.5974	10.8728	19.545	90	90	90	2021	[24]
**AWEROM**	$P2_1$	5.683	14.953	10.758	90	102.85	90	2021	[25]
**AWIFIY**	$P2_1$	8.5316	9.4812	10.8081	90	92.639	90	2021	[26]
**AWIWEL**	$P2_1$	9.3422	20.493	9.4596	90	98.619	90	2021	[27]
**AXUMEO**	$P2_1$	8.6489	8.5729	8.9404	90	94.093	90	2021	[28]
**AZETUX**	$P2_1$	10.2924	10.2096	10.3749	90	97.697	90	2021	[29]
**BACVEK**	$C222_1$	11.1311	25.229	13.9728	90	90	90	2020	[30]
**BADFIZ**	$P2_1$	6.3198	6.4188	14.989	90	100.159	90	2020	[31]
**BOZSUH**	$P2_12_12_1$	5.81156	17.547	18.1227	90	90	90	2020	[32]
**BUDBIO**	$P2_12_12_1$	7.0789	11.0797	25.1729	90	90	90	2020	[33]
**BUDFOY**	$P2_12_12_1$	7.057	13.908	14.326	90	90	90	2020	[34]
**BUDYOR**	$P2_12_12_1$	7.1121	12.1946	16.199	90	90	90	2020	[35]
**BUHZEM**	$P2_12_12_1$	10.3192	13.6281	25.6934	90	90	90	2020	[36]
**BUKZEP**	$P2_12_12_1$	7.5891	10.7774	17.9836	90	90	90	2020	[37]
**BULBAO**	$P2_12_12_1$	8.853	9.9104	18.1445	90	90	90	2020	[38]
**BUSFUT**	$P2_12_12_1$	11.416	11.899	15.552	90	90	90	2020	[39]
**BUYNIV**	$P2_12_12_1$	5.9043	13.8065	14.6027	90	90	90	2020	[40]
**BUYPIX**	$P2_12_12_1$	5.8542	12.9039	18.9035	90	90	90	2020	[40]

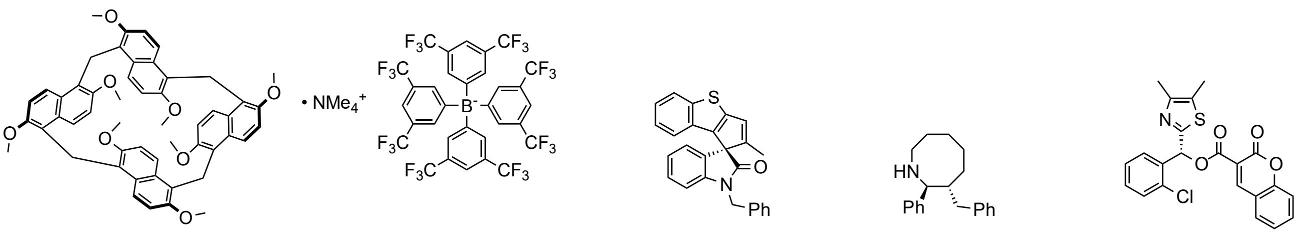

ABULES AHIWAS AHURAZ AJAQIO

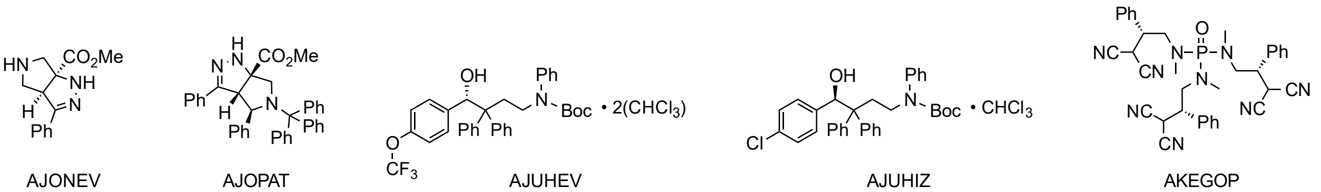

AJONEV AJOPAT AJUHEV AJUHIZ AKEGOP

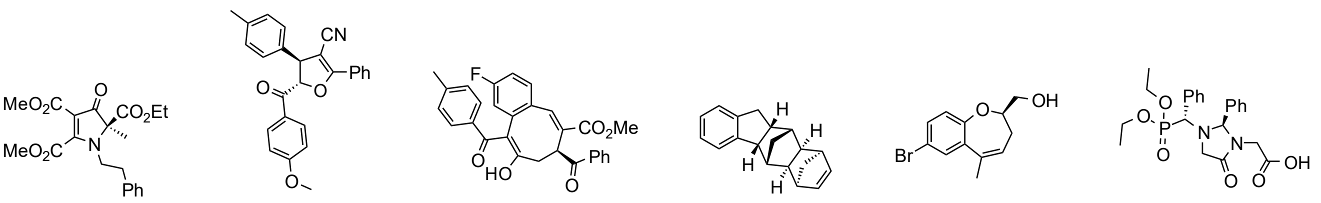

AKIJOW AMESUJ ANETUL ANUVEN APOHOF APUGOK

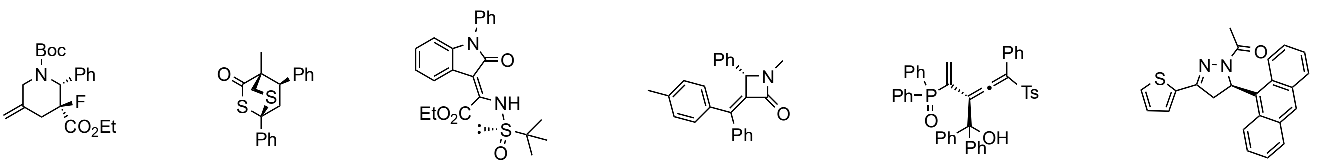

AQETOI AREGAI ARERAX ARIDUD ARIHER AROCES

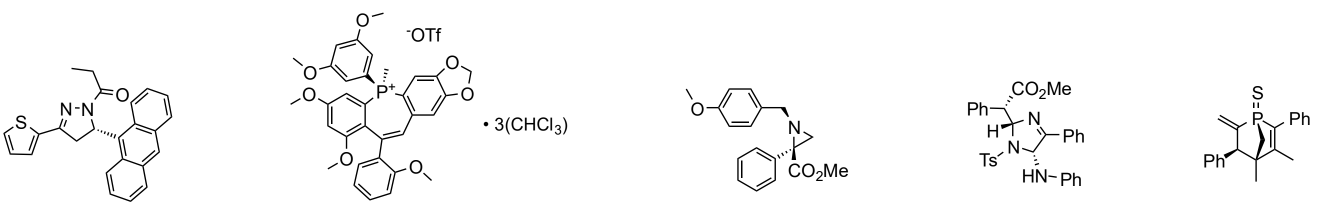

AROCIW ASELOC ASILAS ATAROF ATEROJ

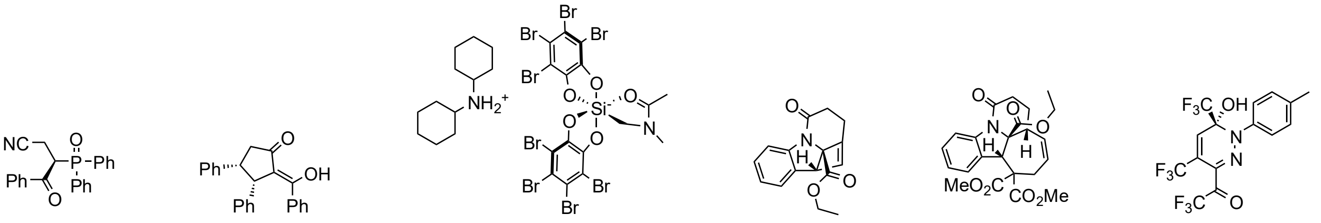

AWEROM AWIFIY AWIWEL AXUMEO AZETUX BACVEK

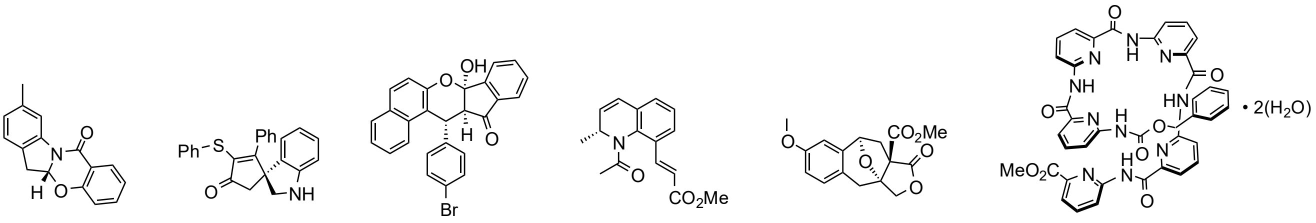

BADFIZ BOZSUH BUDBIO BUDFOY BUDYOR BUHZEM

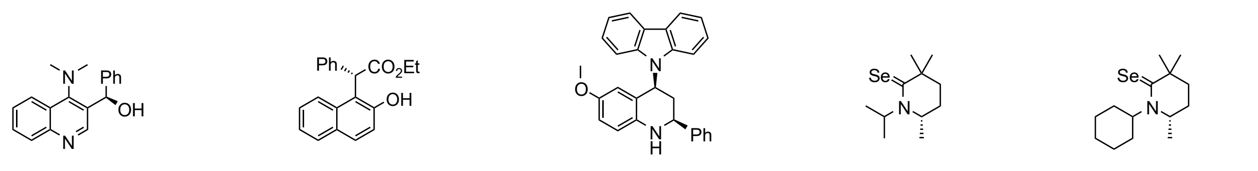

BUKZEP BULBAO BUSFUT BUYNIV BUYPIX

CSD Code	Space Group	a / Å	b / Å	c / Å	α / °	β / °	γ / °	Publication Year	Reference
**CACCOC**	$P2_12_12_1$	7.1141	11.5921	19.595	90	90	90	2020	[41]
**CUGXEK**	$P2_1$	7.8416	8.9737	10.0972	90	103.688	90	2020	[42]
**CURKEI**	$P2_1$	8.8533	13.6405	10.1517	90	94.146	90	2020	[43]
**CUXFAF**	$P2_1$	10.0127	11.0765	10.3165	90	91.013	90	2020	[44]
**CUYNAO**	$P2_12_12_1$	9.0888	10.564	21.463	90	90	90	2020	[45]
**CUYREW**	$P2_1$	12.064	6.135	12.744	90	114.742	90	2020	[46]
**CUYRIA**	$P2_1$	10.302	6.1145	14.508	90	105.269	90	2020	[46]
**DAGLIK**	$P2_12_12_1$	7.4173	10.9085	14.2789	90	90	90	2021	[47]
**DAHSUA01**	$P2_12_12_1$	5.9552	11.992	18.811	90	90	90	2021	[48]
**DAJYUM**	$P2_12_12_1$	8.47196	11.1287	25.959	90	90	90	2021	[49]
**DAKHEG**	$P2_1$	13.697	6.8247	14.818	90	113.94	90	2021	[50]
**DALTIX**	$P2_12_12_1$	10.8628	13.0031	18.3185	90	90	90	2021	[51]
**DUHYIR**	$P2_12_12_1$	12.3466	16.0952	26.4133	90	90	90	2020	[52]
**DUNGUR01**	$P2_12_12_1$	6.0044	10.0276	30.04	90	90	90	2020	[53]
**DUNXOC**	$P2_12_12_1$	8.7643	9.1745	20.1935	90	90	90	2020	[54]
**DURMOV**	$P2_12_12_1$	6.0732	11.111	29.291	90	90	90	2020	[55]
**EBOHIQ**	$P2_1$	8.057	14.0475	12.2644	90	95.457	90	2021	[56]
**ECAFUN**	$P2_12_12_1$	8.155	10.374	12.7178	90	90	90	2021	[57]
**EHIYIG**	$P2_12_12_1$	6.0526	8.1592	26.617	90	90	90	2020	[58]
**EKIGEN**	$P2_12_12_1$	8.122	10.346	19.193	90	90	90	2021	[59]
**EKIGUD**	$P2_12_12_1$	10.49	10.541	13.3225	90	90	90	2021	[59]
**EMEXOM**	$P2_12_12_1$	10.3181	12.6189	13.9769	90	90	90	2021	[60]
**EMEYON**	$P2_12_12_1$	10.6059	13.0397	13.6137	90	90	90	2021	[60]
**EMUZAQ**	$P2_12_12_1$	4.8091	10.5257	35.4617	90	90	90	2021	[61]
**EMUZEU**	$P2_12_12_1$	4.8872	10.5776	35.399	90	90	90	2021	[61]
**ENAWUO**	$P2_12_12_1$	10.058	11.363	16.781	90	90	90	2020	[62]
**ENEBUX**	$P2_12_12_1$	5.484	11.3292	24.217	90	90	90	2020	[63]
**ENOYOY**	$P2_1$	5.3407	12.745	18.872	90	92.51	90	2021	[64]
**ENUDEZ**	$P2_12_12_1$	11.7931	14.9063	16.3539	90	90	90	2021	[65]
**EPAPAP**	$P2_12_12_1$	8.4316	9.5742	19.0433	90	90	90	2021	[66]
**EPAROF**	$P2_12_12_1$	8.3029	12.1063	20.187	90	90	90	2021	[67]
**EQOQUZ**	$P2_12_12_1$	7.8737	11.9021	22.0911	90	90	90	2021	[68]
**ESEPUQ**	$P2_12_12_1$	8.827	10.3033	10.9546	90	90	90	2021	[69]
**ESIKOJ01**	$P2_12_12_1$	10.3098	11.4153	12.3089	90	90	90	2021	[70]
**ETEGOC**	$P6_1$	19.9112	19.9112	9.2146	90	90	120	2021	[71]
**ETOCUO**	$P2_1$	11.8138	4.7616	12.089	90	101.212	90	2021	[72]
**EVILAZ**	$P2_1$	11.5552	10.9344	12.4336	90	114.632	90	2021	[73]
**EWATOO**	$P2_12_12_1$	8.6663	10.2215	16.751	90	90	90	2021	[74]
**EWIWEP**	$P2_1$	5.8685	13.0985	6.8273	90	95.211	90	2021	[75]
**EWIZES**	$P2_12_12_1$	12.6733	22.4008	23.4422	90	90	90	2021	[76]

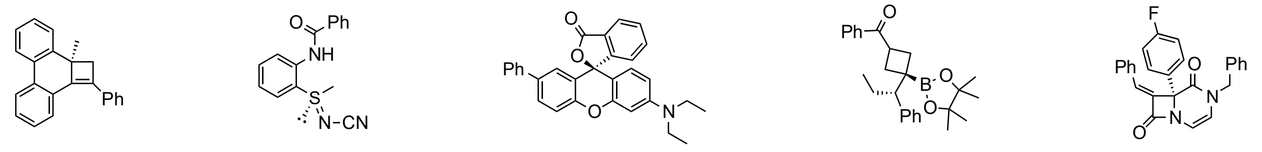

CACCOC CUGXEK CURKEI CUXFAF CUYNAO

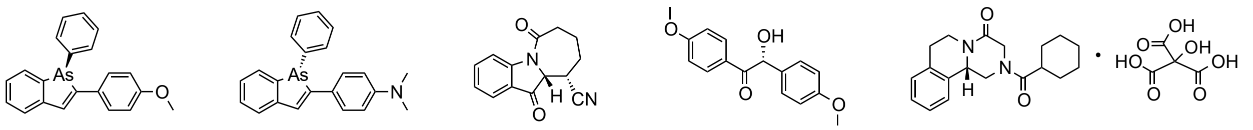

CUYREW CUYRIA DAGLIK DAHSUA01 DAJYUM

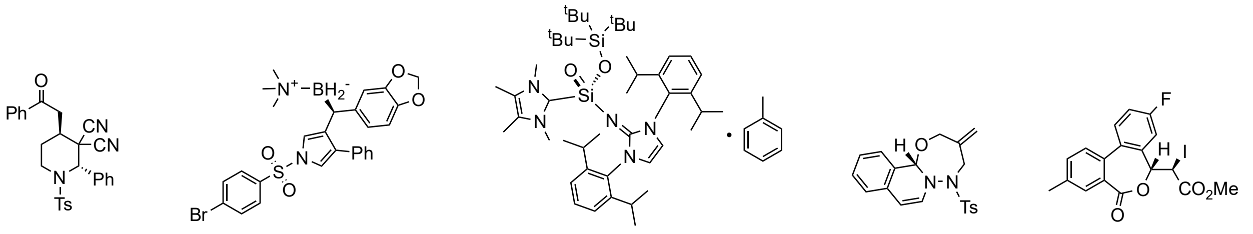

DAKHEG DALTIX DUHYIR DUNGUR01 DUNXOC

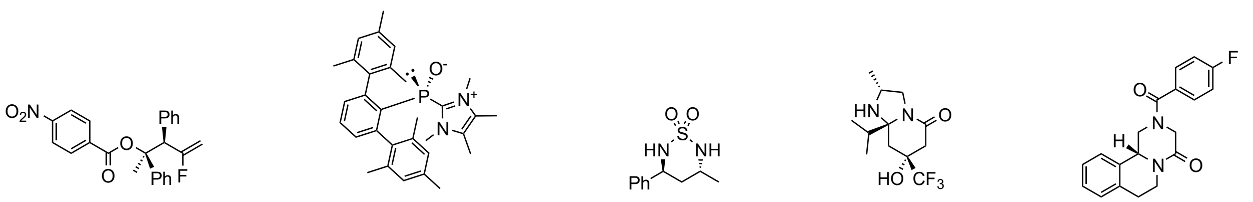

DURMOV EBOHIQ ECAFUN EHIYIG EKIGEN

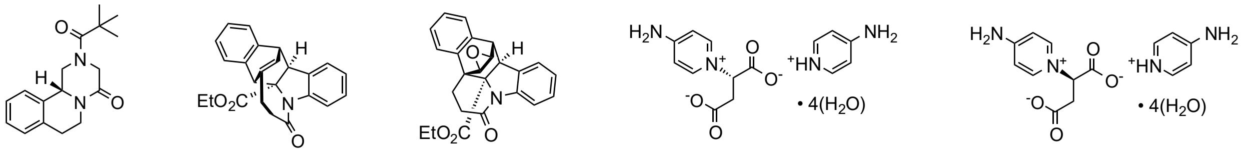

EKIGUD EMEXOM EMEYON EMUZAQ EMUZEU

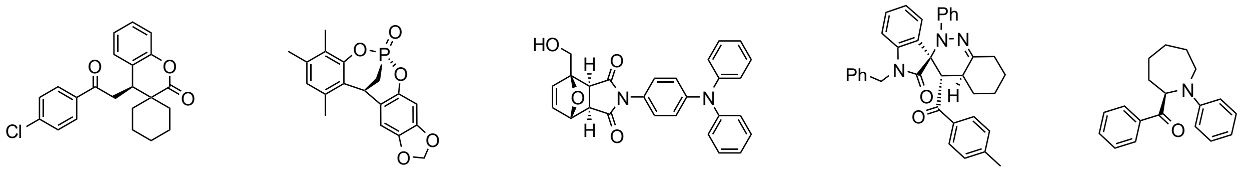

ENAWUO ENEBUX ENOYOY ENUDEZ EPAPAP

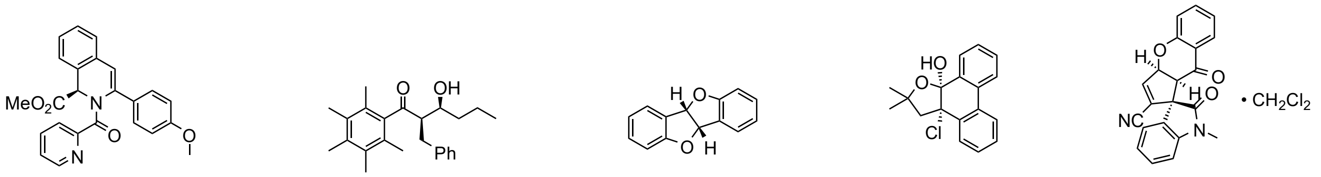

EPAROF EQOQUZ ESEPUQ ESIKOJ01 ETEGOC

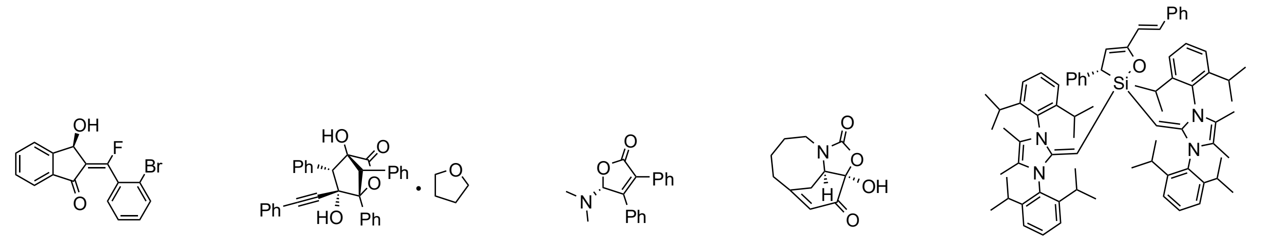

ETOCUO EVILAZ EWATOO EWIWEP EWIZES

CSD Code	Space Group	a / Å	b / Å	c / Å	α / °	β / °	γ / °	Publication Year	Reference
**EXIROV**	$P2_12_12_1$	5.81119	17.0903	19.3413	90	90	90	2021	[77]
**EYELUS**	$P4_1$	12.54	12.54	10.796	90	90	90	2021	[78]
**EYEMIH**	$P2_12_12_1$	5.1548	8.9662	29.347	90	90	90	2021	[78]
**EYIREM**	$P2_12_12_1$	9.9174	11.3714	14.2774	90	90	90	2020	[79]
**EZUBUZ**	$P2_12_12_1$	8.9528	12.4499	26.0558	90	90	90	2021	[80]
**FAGCUP**	$P2_12_12_1$	9.2598	10.046	18.4675	90	90	90	2020	[81]
**FAHSEQ**	$P2_12_12_1$	11.6435	12.6568	14.0232	90	90	90	2020	[82]
**FAKHUY**	$P2_12_12_1$	8.70779	9.8648	18.3289	90	90	90	2020	[83]
**FAKPAM**	$P2_12_12_1$	6.704	13.151	14.254	90	90	90	2020	[84]
**FAKQAN**	$C2221$	13.4355	14.6914	19.5353	90	90	90	2020	[85]
**FULQAH**	$P2_12_12_1$	5.806	8.5036	29.3026	90	90	90	2020	[86]
**FAGCUP**	$P2_12_12_1$	9.2598	10.046	18.4675	90	90	90	2020	[81]
**FAHSEQ**	$P2_12_12_1$	11.6435	12.6568	14.0232	90	90	90	2020	[82]
**FAKHUY**	$P2_12_12_1$	8.70779	9.8648	18.3289	90	90	90	2020	[83]
**GUPBOL**	$P2_1$	7.4839	14.1752	9.6461	90	110.537	90	2020	[87]
**GUPBUR**	$P2_12_12_1$	7.9845	8.5641	25.649	90	90	90	2020	[87]
**GUPHAD**	$P2_1$	5.7173	7.346	12.436	90	93.07	90	2020	[88]
**GUTMIU**	$P2_1$	8.21	6.33	17.277	90	102.41	90	2020	[89]
**GUTNAN**	$P2_1$	8.317	6.0774	17.513	90	103.095	90	2020	[89]
**GUVQOG**	$P2_12_12_1$	7.4006	12.058	14.3282	90	86	89	2020	[90]
**HAKPIW**	$P2_12_12_1$	5.6662	10.734	14.6887	90	90	90	2020	[91]
**HUFQEH**	$P2_12_12_1$	7.6871	13.649	25.234	90	90	90	2020	[92]
**HUGBOD**	$P2_12_12_1$	6.6852	15.879	17.665	90	90	90	2020	[93]
**HUHKOM01**	$P2_12_12_1$	17.6357	14.1494	5.8272	90	90	90	2020	[94]
**HUPHUY**	$P2_1$	7.3669	5.9405	17.4582	90	91.628	90	2020	[95]
**HUSTAT**	$P2_12_12_1$	10.8391	15.1936	18.0184	90	90	90	2020	[96]
**HUVLOC**	$P2_12_12_1$	5.6655	13.092	21.163	90	90	90	2020	[97]
**HUWYEG**	$P2_12_12_1$	7.2273	12.6042	29.1348	90	90	90	2020	[98]
**IBAMEH**	$P2_1$	6.9979	7.7768	15.6987	90	99.432	90	2021	[99]
**IBUDUI**	$P2_12_12_1$	8.5928	9.5612	16.2090	90	90	90	2021	[100]
**ICAJAB**	$P2_12_12_1$	8.3484	13.1045	30.1609	90	90	90	2021	[101]
**IGEZEC**	$P3_221$	16.177	16.177	10.333	90	90	120	2020	[102]
**IGIXII**	$P2_12_12_1$	6.5205	6.9248	36.1	90	90	90	2020	[103]
**IGIXOO**	$P2_12_12_1$	6.523	6.9313	36.119	90	90	90	2020	[103]
**IJIRUR**	$P2_1$	12.3846	8.6553	14.6888	90	90.5734	90	2021	[104]
**IKIDIS**	$P2_12_12_1$	6.5045	12.3554	25.1783	90	90	90	2021	[105]
**ILITIJ**	$P2_12_12_1$	7.91738	8.4298	23.2024	90	90	90	2021	[106]
**ILUVAP**	$P2_12_12_1$	10.051	10.6294	15.6476	90	90	90	2021	[107]
**INIDOA01**	$P2_12_12_1$	9.382	11.151	12.086	90	90	90	2020	[108]
**INOREL**	$P2_12_12_1$	8.47103	9.75285	18.4757	90	90	90	2021	[109]
**IPOJUV**	$P2_12_12_1$	11.5266	11.6338	16.6421	90	90	90	2021	[110]
**IRIZUH**	$P2_12_12_1$	7.2357	14.587	17.3437	90	90	90	2021	[111]
**ISAROM**	$P2_12_12_1$	7.418	8.3999	27.5589	90	90	90	2021	[112]

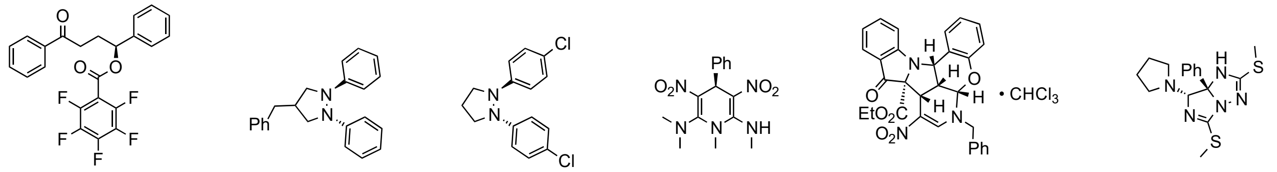

EXIROV EYELUS EYEMIH EYIREM EZUBUZ FAGCUP

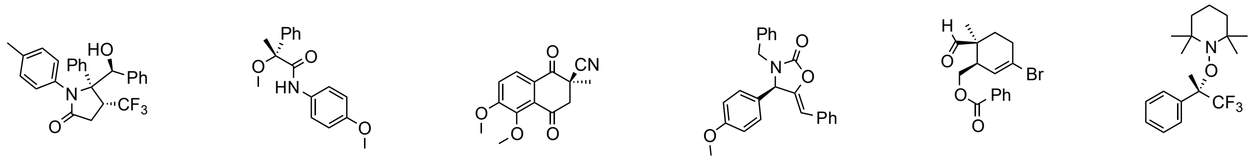

FAHSEQ FAKHUY FAKPAM FAKQAN FULQAH FUNLIM

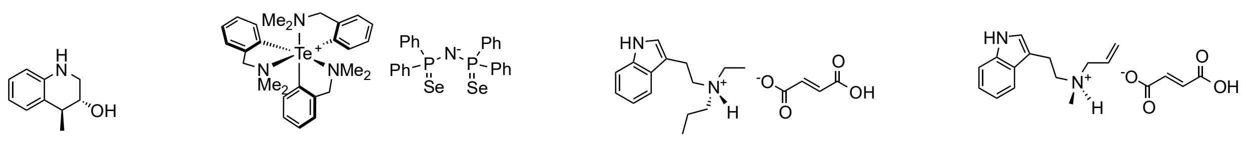

GUBDEP GUNGEE GUPBOL GUPBUR

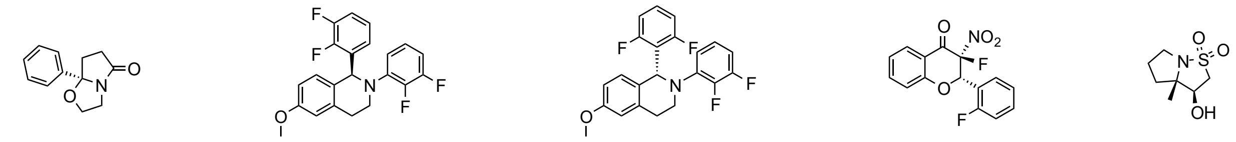

GUPHAD GUTMIU GUTNAN GUVQOG HAKPIW

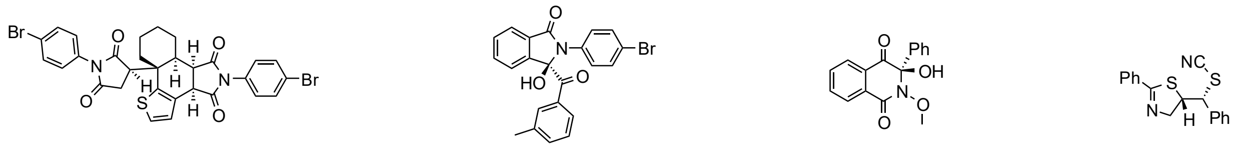

HUFQEH HUGBOD HUHKOM01 HUPHUY

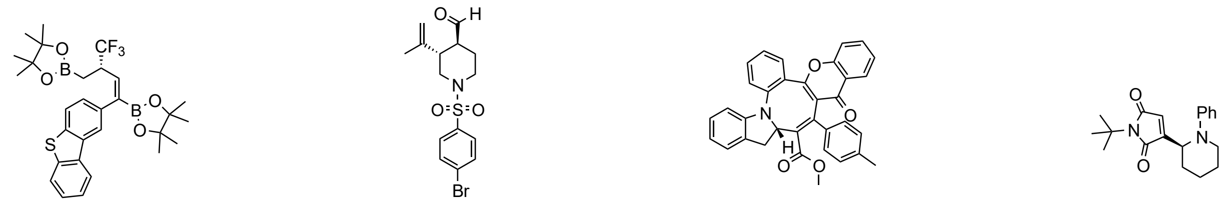

HUSTAT HUVLOC HUWYEG IBAMEH

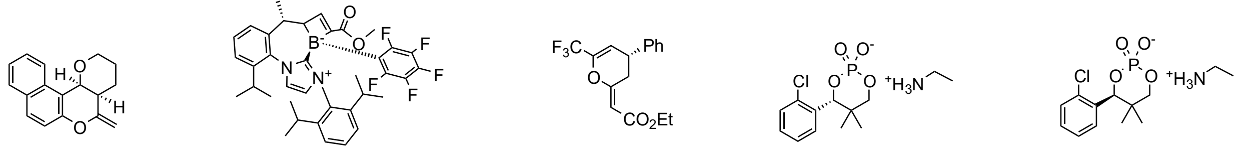

IBUDUI ICAJAB IGEZEC IGIXII IGIXOO

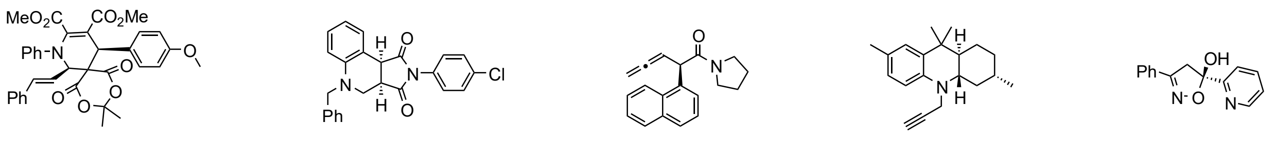

IJIRUR IKIDIS ILITIJ ILUVAP INIDOA01

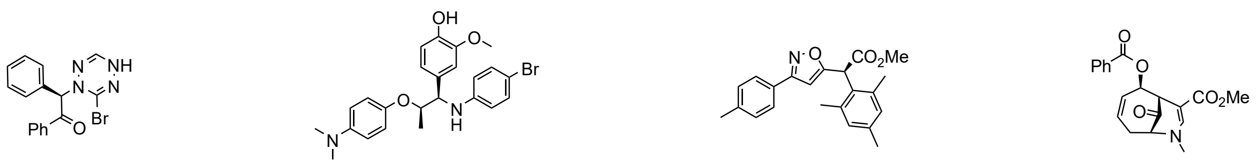

INOREL IPOJUV IRIZUH ISAROM

CSD Code	Space Group	a / Å	b / Å	c / Å	α / °	β / °	γ / °	Publication Year	Reference
**ISEFIY**	$P2_1$	4.7394	10.4283	11.7914	90	96.08	90	2021	[113]
**ISELUQ**	$P2_12_12_1$	5.1676	14.2304	23.7653	90	90	90	2021	[114]
**ITEREH**	$P2_12_12_1$	9.1649	11.9976	23.8507	90	90	90	2021	[115]
**ITUDEJ**	$P2_1$	5.3757	16.645	9.8936	90	93.728	90	2021	[116]
**IVUJUH**	$P2_12_12_1$	20.9403	15.0638	6.3033	90	90	90	2021	[117]
**IWIWUJ**	$P2_12_12_1$	9.2258	15.495	15.6334	90	90	90	2021	[118]
**IYETAK**	$P2_12_12_1$	8.315	11.835	16.239	90	90	90	2021	[119]
**IYOKEP**	$P2_12_12_1$	5.73769	14.639	18.3806	90	90	90	2021	[120]
**IYOKIT**	$P2_12_12_1$	8.2451	10.6533	16.2781	90	90	90	2021	[120]
**IYUNOI**	$P2_12_12_1$	10.3676	11.5961	17.3669	90	90	90	2021	[121]
**IYUXUY**	$P2_12_12_1$	7.5516	10.6961	18.4676	90	90	90	2021	[122]
**IZUHAP**	$P2_1$	10.0647	5.9175	19.388	90	104.726	90	2021	[123]
**JOYZEF**	$P2_12_12_1$	9.724	14.9525	16.4897	90	90	90	2020	[124]
**JOZFEM**	$P2_12_12_1$	8.16714	11.261	14.2325	90	90	90	2020	[125]
**JOZTUQ**	$P2_12_12_1$	9.0236	9.2931	20.0094	90	90	90	2020	[125]
**JUNNIS**	$P2_12_12_1$	7.905	7.9314	11.8753	90	90	90	2020	[126]
**JUNRAO**	$P2_12_12_1$	9.6863	12.2535	14.955	90	90	90	2020	[127]
**JUVKIX01**	$P2_1$	10.5252	7.625	10.494	90	104.938	90	2020	[128]
**JUXGAN**	$P2_12_12_1$	10.906	13.6614	16.6806	90	90	90	2020	[129]
**JUZKUN**	$P2_12_12_1$	11.3284	11.8731	15.1405	90	90	90	2020	[130]
**KADKEJ**	$P2_12_12_1$	5.6056	17.422	22.161	90	90	90	2020	[131]
**KADKOT**	$P2_1$	5.4142	20.89	8.3858	90	92.126	90	2020	[131]
**KAKXON**	$P2_12_12_1$	34.8779	8.8009	5.7937	90	90	90	2020	[132]
**KUGRIQ**	$P2_12_12_1$	7.3643	8.6863	22.636	90	90	90	2020	[133]
**KULMUC**	R3	23.4012	23.4012	5.73617	90	90	120	2020	[134]
**KUQFUA**	$P2_1$	7.157	7.4823	11.605	90	90.91	90	2020	[135]
**KUYPIG**	$P2_1$	9.3504	15.821	11.3649	90	111.435	90	2020	[136]
**LACGIJ**	$P2_1$	8.8532	13.7373	11.5286	90	107.761	90	2020	[137]
**LAKCAF**	$P2_12_12_1$	9.3468	11.3221	15.0175	90	90	90	2020	[138]
**LULWAT**	$P2_12_12_1$	7.729	14.599	15.765	90	90	90	2020	[139]
**LUTRAW**	$P2_1$	11.4495	5.7017	14.095	90	107.697	90	2020	[140]
**LUTREA**	$P2_1$	11.7753	5.7683	13.2106	90	105.956	90	2020	[140]
**LUVGAN**	$P2_1$	11.1124	5.2113	13.3792	90	93.648	90	2020	[141]
**MABBOK01**	$P2_12_12_1$	9.6194	11.0463	21.0521	90	90	90	2021	[142]
**MABCAX**	$P2_1$	5.7372	7.241	18.191	90	96.355	90	2021	[143]
**MABCEB**	$P2_12_12_1$	8.3204	11.5226	29.538	90	90	90	2020	[144]
**MALVOO**	$P1$	5.9286	8.2863	8.6201	109.21	90.208	103.829	2020	[145]
**MUDYOC**	$P2_1$	6.2036	8.1137	15.041	90	96.162	90	2020	[146]
**MUGYUL**	$P2_12_12_1$	5.5616	8.8633	30.771	90	90	90	2020	[147]
**MUGZAS**	$P2_12_12_1$	5.571	8.8183	32.576	90	90	90	2020	[147]
**MUGZEW**	$P2_12_12_1$	5.5425	8.8366	31.286	90	90	90	2020	[147]
**MUSHOA**	$P2_12_12_1$	8.8627	10.1024	13.6239	90	90	90	2020	[148]
**NAGJOY**	$P2_12_12_1$	11.2244	14.9261	15.9902	90	90	90	2020	[149]
**NALBEL**	$P2_1$	12.1261	5.3231	15.7434	90	99.951	90	2020	[150]
**NALDAJ**	$P2_1$	12.8242	5.186	14.912	90	99.717	90	2020	[150]
**NALDEN**	$P2_1$	13.111	5.176	14.827	90	99.291	90	2020	[150]
**NUCGEA02**	$P2_12_12_1$	6.5975	12.3565	16.7223	90	90	90	2020	[151]
**NUCGOK01**	$P2_12_12_1$	7.9707	12.9386	13.7305	90	90	90	2020	[151]

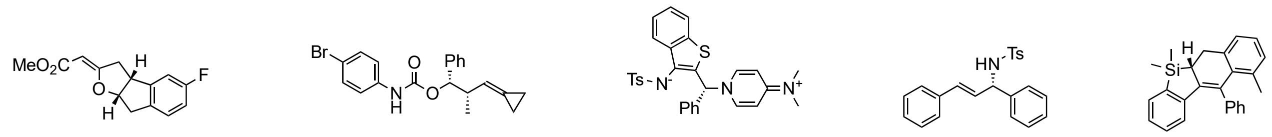

ISEFIY ISELUQ ITEREH ITUDEJ IVUJUH

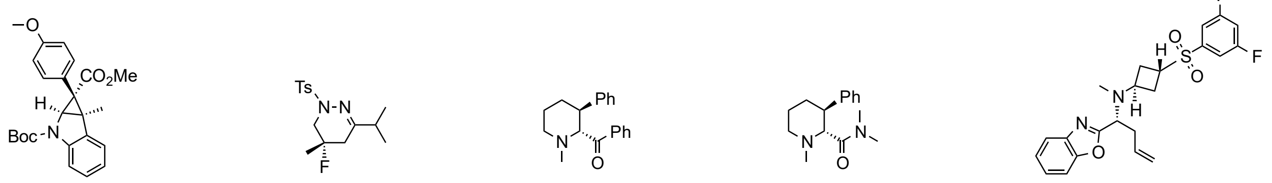

IWIWUJ IYETAK IYOKEP IYOKIT IYUNOI

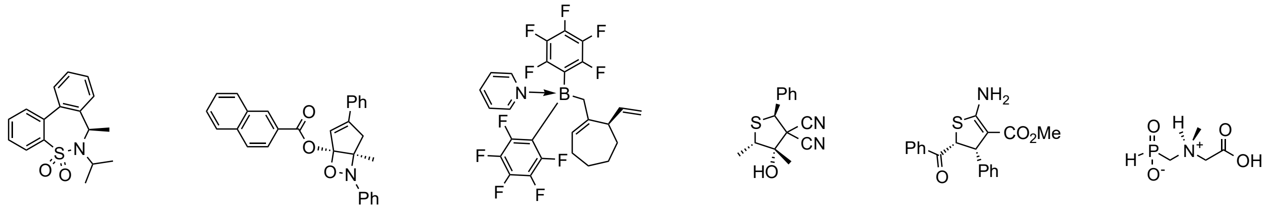

IYUXUY IZUHAP JOYZEF JOZFEM JOZTUQ JUNNIS

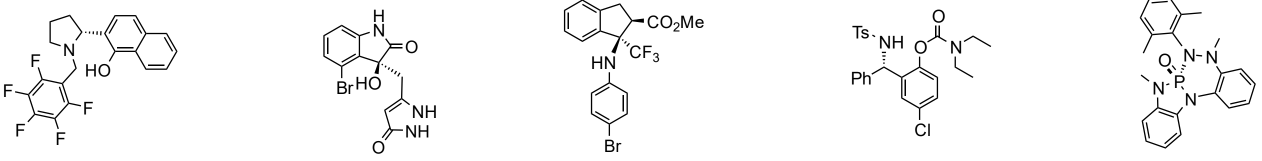

JUNRAO JUPKOX JUVKIX01 JUXGAN JUZKUN

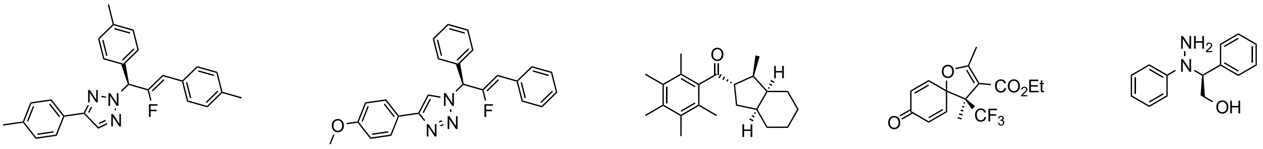

KADKEJ KADKOT KAKXON KUGRIQ KULMUC

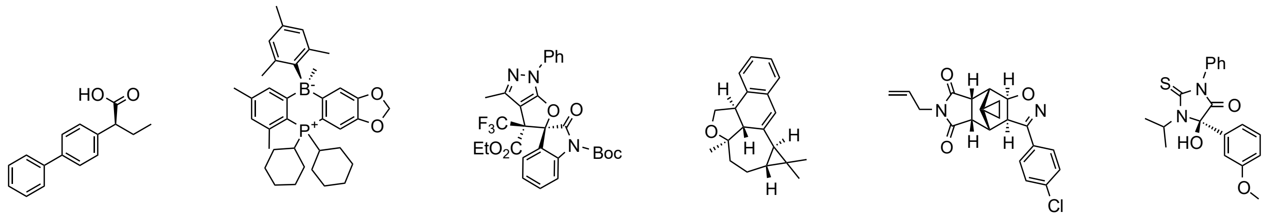

KUQFUA KUYPIG LACGIJ LAKCAF LULWAT LUTRAW

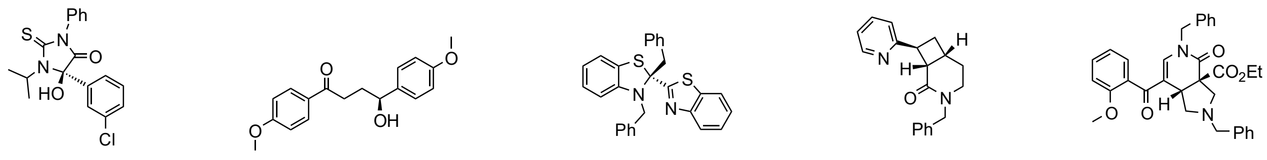

LUTREA LUVGAN MABBOK01 MABCAX MABCEB

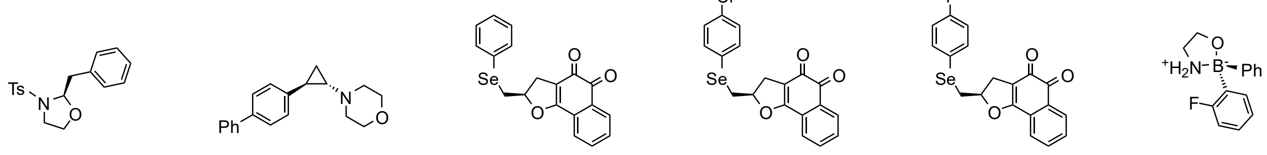

MALVOO MUDYOC MUGYUL MUGZAS MUGZEW MUSHOA

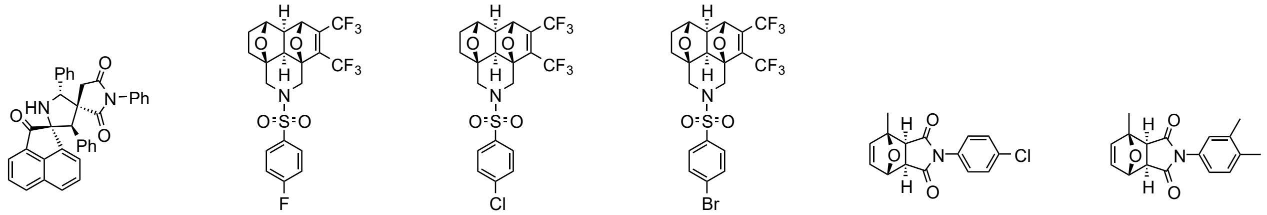

NAGJOY NALBEL NALDAJ NALDEN NUCGEA02 NUCGOK01

CSD Code	Space Group	a / Å	b / Å	c / Å	α / °	β / °	γ / °	Publication Year	Reference
**NUCGUQ01**	$P2_1$	5.4032	13.9697	9.6673	90	105.179	90	2020	[151]
**NUCHAX01**	$P2_1$	5.3713	13.958	9.5751	90	103.609	90	2020	[151]
**NUCHEB01**	$P2_12_12_1$	9.3007	10.6635	13.701	90	90	90	2020	[151]
**NUDQAH**	$P2_12_12_1$	8.6036	12.8834	14.9608	90	90	90	2020	[152]
**NUKTUL**	$P2_1$	8.4181	10.0355	10.2655	90	107.956	90	2020	[153]
**NUTPAW**	$P2_12_12_1$	7.1213	9.6599	24.5371	90	90	90	2020	[154]
**NUWMOK**	$P2_12_12_1$	8.8629	10.5496	21.448	90	90	90	2020	[155]
**NUXROQ**	$C2$	19.357	5.7941	21.216	90	115.066	90	2020	[156]
**NUYYOY**	$P2_12_12_1$	9.2745	11.3831	27.5962	90	90	90	2020	[157]
**OBOLAW**	$P2_12_12_1$	11.336	11.9463	16.0968	90	90	90	2021	[158]
**OCEKAM**	$P2_1$	8.1825	7.1124	10.5177	90	91.004	90	2021	[159]
**OCODOD**	$P2_12_12_1$	5.436	8.208	25.76	90	90	90	2021	[160]
**OCOFIZ**	$P2_1$	8.084	6.646	13.535	90	106.929	90	2021	[160]
**OCUGOM**	$P2_12_12_1$	11.1081	11.5891	12.8087	90	90	90	2021	[161]
**OHAVIF**	$P2_1$	5.7163	15.9831	13.7113	90	98.507	90	2020	[162]
**OHEJAP**	$P2_12_12_1$	8.51025	10.0196	30.6766	90	90	90	2020	[163]
**OMENAY**	$P2_1$	6.8601	20.5081	7.345	90	92.708	90	2021	[164]
**OMEXAI**	$P2_1$	9.2987	9.1501	12.3596	90	98.331	90	2021	[165]
**ONOYAU**	$P2_12_12_1$	10.3911	13.0014	24.381	90	90	90	2021	[166]
**ONOYIC**	$P2_12_12_1$	11.7538	12.8616	21.8976	90	90	90	2021	[166]
**OROQIY**	$P2_12_12_1$	9.3776	9.3907	10.4759	90	90	90	2021	[167]
**OSEYAP**	$P2_1$	7.076	8.203	13.853	90	97.152	90	2021	[168]
**OSUNEY**	$P6_1$	9.6734	9.6734	31.308	90	90	120	2021	[169]
**OSUNEY01**	$P2_1$	9.0476	8.5404	11.4193	90	113.152	90	2021	[169]
**OSUTAA**	$P2_12_12_1$	5.7593	16.9426	17.4689	90	90	90	2021	[170]
**OSUTII**	$P2_12_12_1$	5.7418	16.9894	17.3413	90	90	90	2021	[170]
**OSUTUU**	$P2_12_12_1$	5.686	16.9458	17.3485	90	90	90	2021	[170]
**OTIYUO01**	$P2_12_12_1$	5.8485	15.7132	16.2264	90	90	90	2021	[171]
**OTURON**	$P2_12_12_1$	5.6882	11.4345	27.5456	90	90	90	2021	[172]
**OVENAH**	$P2_1$	9.7431	6.9459	12.1403	90	105.257	90	2021	[173]
**OVEPEN**	$P2_12_12_1$	6.1138	11.1707	33.835	90	90	90	2021	[173]
**OVEYUM**	$P3_2$	9.8166	9.8166	14.9871	90	90	120	2021	[174]
**OVUKOI**	$P2_12_12_1$	6.0655	16.0001	17.3309	90	90	90	2021	[175]
**OWAQIP**	$C2$	14.1976	11.5651	12.5866	90	93.1742	90	2021	[176]
**OWOCAH**	$P2_1$	6.6459	9.1822	12.3862	90	95.365	90	2021	[177]
**OWUREG**	$P2_12_12_1$	8.0799	10.1073	13.937	90	90	90	2020	[178]
**OXACEY**	$P2_12_12_1$	6.0042	15.3676	19.9448	90	90	90	2021	[179]
**OXIJAJ**	$P2_12_12_1$	7.6661	13.863	19.578	90	90	90	2021	[180]
**OXUTIN**	$P2_12_12_1$	8.6947	11.949	21.1646	90	90	90	2021	[181]
**OYOHOC**	$P2_1$	7.6486	6.0185	15.9086	90	103.605	90	2021	[182]
**OZILAN**	$P2_12_12_1$	5.9908	11.5506	16.8253	90	90	90	2021	[183]
**OZOBOX**	$P2_12_12_1$	5.86313	16.7345	18.2562	90	90	90	2021	[184]
**POXGUH01**	$P2_1$	10.489	7.9983	12.7315	90	113.063	90	2020	[185]
**PUGQEQ**	$P2_12_12_1$	8.0469	10.7636	22.9804	90	90	90	2020	[186]
**PUHBIG**	$P2_12_12_1$	5.5128	14.1945	20.649	90	90	90	2020	[187]
**PUNJUG**	$P2_12_12_1$	8.5365	12.2999	16.448	90	90	90	2020	[188]
**PUQTED**	$P2_12_12_1$	5.5308	14.3198	18.1491	90	90	90	2020	[189]
**QAHWOP**	$P2_12_12_1$	11.6625	12.2376	21.7465	90	90	90	2020	[190]

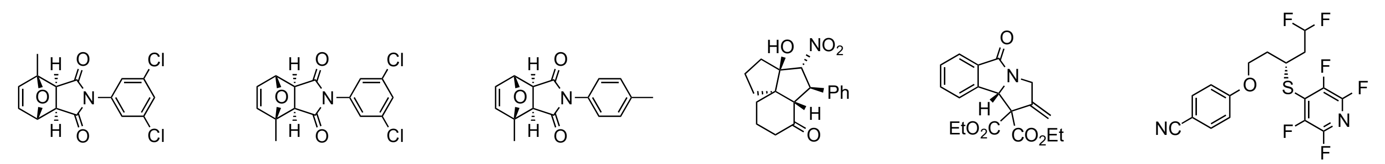

NUCGUQ01 NUCHAX01 NUCHEB01 NUDQAH NUKTUL NUTPAW

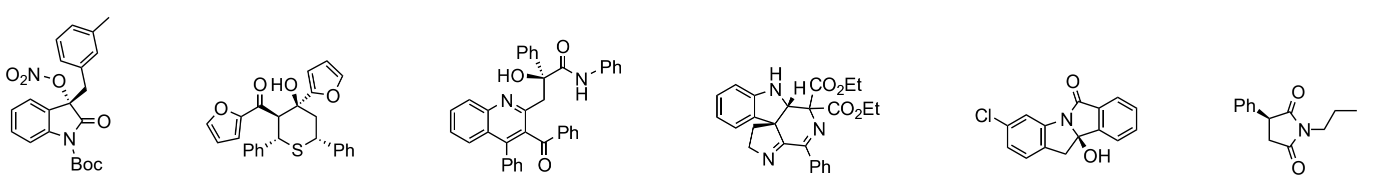

NUWMOK NUXROQ NUYYOY OBOLAW OCEKAM OCODOD

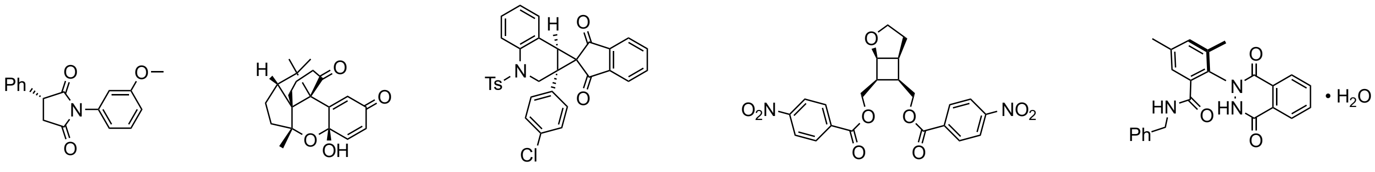

OCOFIZ OCUGOM OHEJAP OMENAY OMEXAI

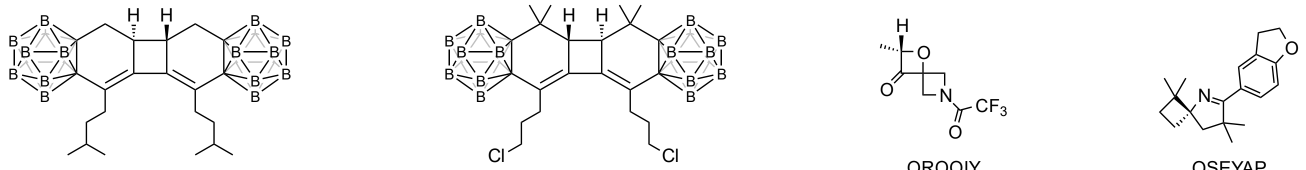

ONOYAU ONOYIC OROQIY OSEYAP

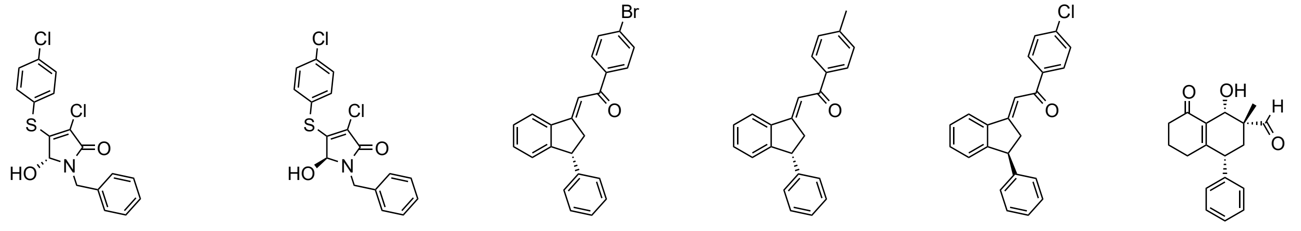

OSUNEY OSUNEY01 OSUTAA OSUTII OSUTUU OTIYUO01

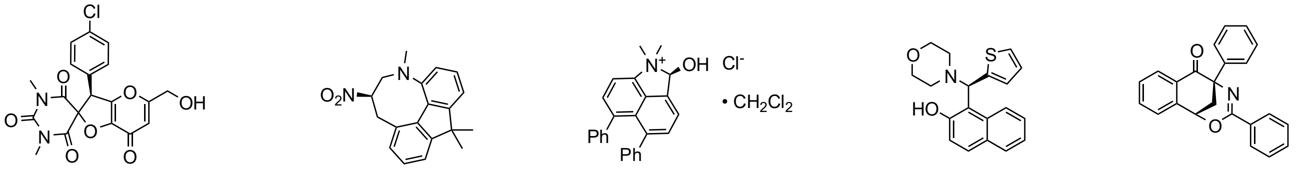

OTURON OVENAH OVEPEN OVEYUM OVUKOI

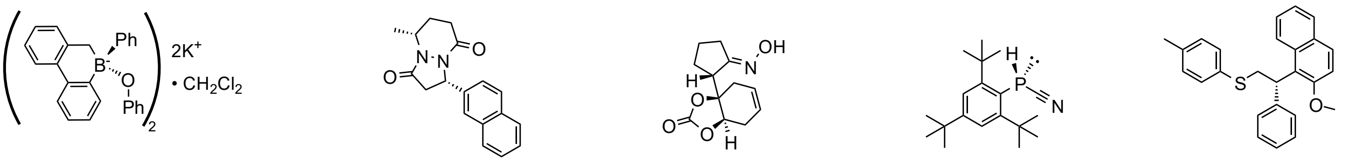

OWAQIP OWOCAH OWUREG OXACEY OXIJAJ

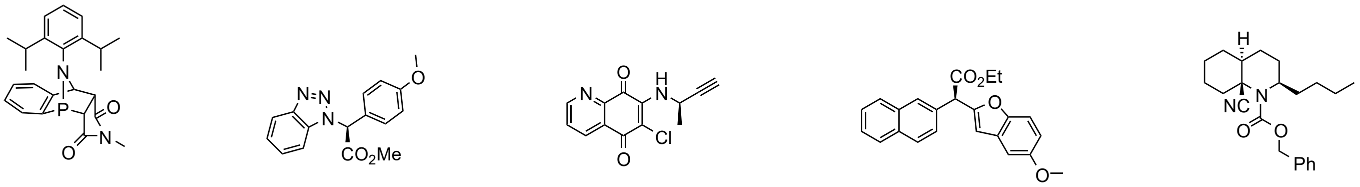

OXUTIN OYOHOC OZILAN OZOBOX POXGUH01

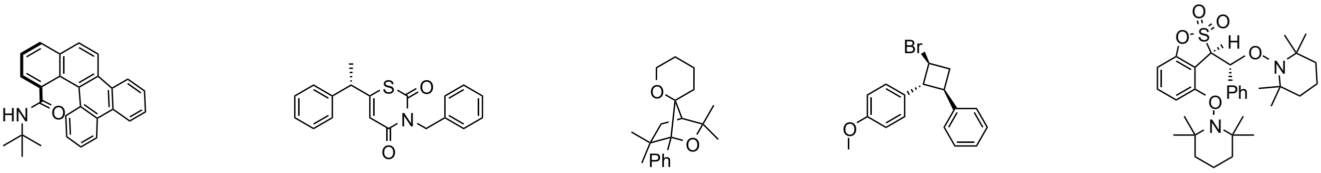

PUGQEQ PUHBIG PUNJUG PUQTED QAHWOP

CSD Code	Space Group	a / Å	b / Å	c / Å	α / °	β / °	γ / °	Publication Year	Reference
**QAKSEE**	$P2_12_12_1$	8.0361	12.9083	15.8908	90	90	90	2020	[191]
**QAKTAB**	$P2_1$	6.4896	9.5675	17.5232	90	99.892	90	2020	[192]
**QAKTEF**	$P2_12_12_1$	7.2968	9.5536	26.3314	90	90	90	2020	[193]
**QOSFAI**	$P2_12_12_1$	13.9773	17.9172	19.0021	90	90	90	2020	[194]
**QUJVID**	$P2_1$	9.571	9.2696	12.9263	90	104.86	90	2020	[195]
**QUKWUR**	$P2_1$	16.179	8.8178	17.233	90	103.819	90	2020	[196]
**QUQJIY**	$P4_32_12$	10.2523	10.2523	39.123	90	90	90	2020	[197]
**QUSNIE**	$P6_5$	16.3287	16.3287	10.7866	90	90	120	2020	[198]
**QUZYOC**	$P2_12_12_1$	5.442	8.871	28.748	90	90	90	2020	[199]
**QUZYUI**	$P2_12_12_1$	5.6145	8.7362	30.511	90	90	90	2020	[199]
**QUZZAP**	$P2_12_12_1$	5.4955	8.8034	29.525	90	90	90	2020	[199]
**RAFFEN**	$P2_12_12_1$	8.4085	9.5736	19.3778	90	90	90	2020	[200]
**RUCDOL**	$P2_1$	10.9732	15.7853	11.7248	90	117.543	90	2020	[201]
**RUGXOJ**	$P2_12_12_1$	14.205	14.517	18.553	90	90	90	2020	[202]
**RUPFUG**	$P2_12_12_1$	12.2814	12.4871	16.249	90	90	90	2020	[203]
**RURDIU**	$P2_12_12_1$	7.3917	8.1635	17.6951	90	90	90	2020	[204]
**RURNAW**	$P2_12_12_1$	6.7939	15.023	25.181	90	90	90	2020	[205]
**SUDVAR**	$P2_1$	12.6613	4.9988	14.716	90	102.355	90	2020	[206]
**SUFYEA**	$P2_1$	11.3905	6.8267	18.0192	90	102.296	90	2020	[207]
**SUGNAM**	$P2_12_12_1$	8.8499	15.4232	16.1861	90	90	90	2020	[208]
**SUHCOQ**	$P2_12_12_1$	5.7894	8.6724	27.3176	90	90	90	2020	[209]
**SUHFEJ**	$P2_12_12_1$	8.5611	13.8879	19.492	90	90	90	2020	[210]
**SUHKOY**	$P2_12_12_1$	8.6975	11.1079	19.6779	90	90	90	2020	[211]
**SULHOZ**	$P1$	5.0939	9.5167	10.1153	106.941	97.064	102.411	2020	[212]
**SUNKUK**	$P2_12_12_1$	7.49	13.833	18.496	90	90	90	2020	[213]
**SUTMOM**	$P2_12_12_1$	10.127	10.7963	21.9636	90	90	90	2020	[214]
**SUWKAZ**	$P2_1$	9.9459	7.2728	9.9756	90	110.265	90	2020	[215]
**SUWKON**	$P2_12_12_1$	7.7375	8.3001	20.809	90	90	90	2020	[215]
**TACGUD**	$P2_1$	6.91475	11.6845	8.08083	90	113.833	90	2020	[216]
**TUCTAP**	$P1$	9.7472	9.7516	10.8108	70.928	67.704	75.286	2020	[217]
**TUDVAS**	$P2_12_12_1$	8.0722	11.6849	19.3159	90	90	90	2020	[218]
**TUFLAK**	$P2_1$	6.5362	9.796	15.874	90	96.468	90	2020	[219]
**TUGVEZ**	$P2_12_12_1$	6.4818	12.5595	24.9955	90	90	90	2020	[220]
**TUHVEA**	$P2_1$	12.1302	6.8745	15.0614	90	112.566	90	2020	[221]
**TUXLAC**	$P2_1$	5.8289	18.094	9.0173	90	95.799	90	2020	[222]
**UCEFIV**	$P2_12_12_1$	8.5467	10.3708	13.7913	90	90	90	2021	[223]
**UCUCOO**	$P2_12_12_1$	6.932	11.065	19.212	90	90	90	2021	[224]
**UCUPAN**	$P2_12_12_1$	13.572	14.179	14.257	90	90	90	2021	[225]
**UGENOM01**	$P2_12_12_1$	6.15494	8.593	23.1982	90	90	90	2020	[226]
**UHABIR**	$P2_1$	11.6605	5.8592	13.2196	90	92.925	90	2020	[227]
**UHECOC**	$P2_12_12_1$	7.9372	12.2884	17.0584	90	90	90	2020	[228]
**UHECUI**	$P2_12_12_1$	8.3318	10.8815	18.0791	90	90	90	2020	[228]
**UKONAM**	$P2_12_12_1$	9.8269	12.5104	17.2187	90	90	90	2020	[229]
**ULACAO**	$P2_12_12_1$	9.1625	13.0052	25.035	90	90	90	2021	[230]
**ULOSUM**	$P2_1$	7.2473	12.2693	10.9251	90	90.353	90	2021	[231]
**ULUTAZ**	$P2_12_12_1$	10.7038	11.6096	17.5182	90	90	90	2021	[232]
**UNOZEF**	$P2_12_12_1$	7.6042	12.5232	19.5798	90	90	90	2021	[233]
**UNUHIX**	$P2_12_12_1$	7.40072	9.1371	18.7024	90	90	90	2021	[234]
**UNUXOT**	$P2_12_12_1$	9.9067	10.0177	22.7535	90	90	90	2021	[235]
**UNUYAG01**	$P2_1$	5.7279	9.0758	17.852	90	94.135	90	2021	[236]
**UPOREZ**	$P2_12_12_1$	7.9048	11.2276	19.3205	90	90	90	2021	[237]

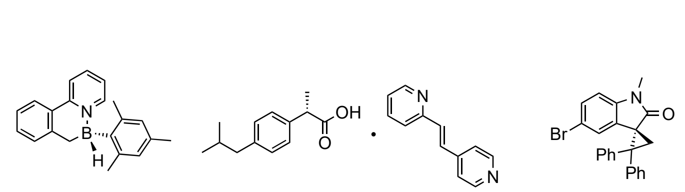

QAKSEE QAKTAB QAKTEF

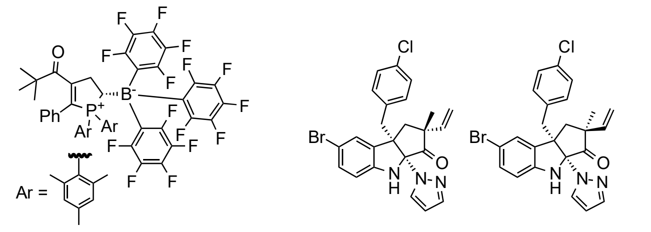

QOSFAI QUJVID

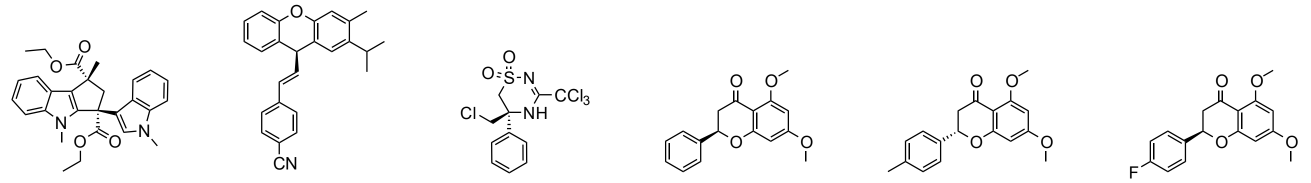

QUKWUR QUQJIY QUSNIE QUZYOC QUZYUI QUZZAP

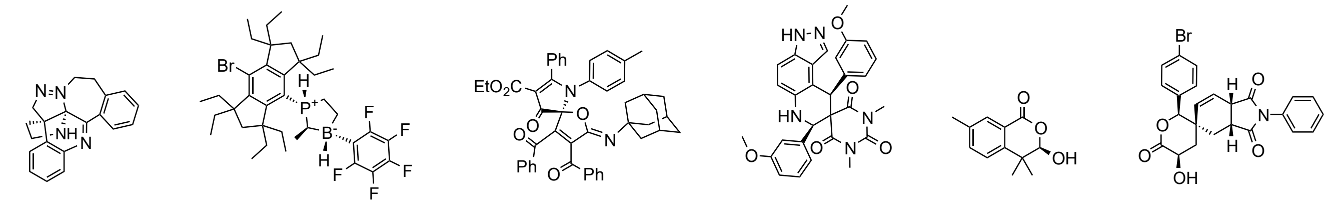

RAFFEN RUCDOL RUGXOJ RUPFUG RURDIU RURNAW

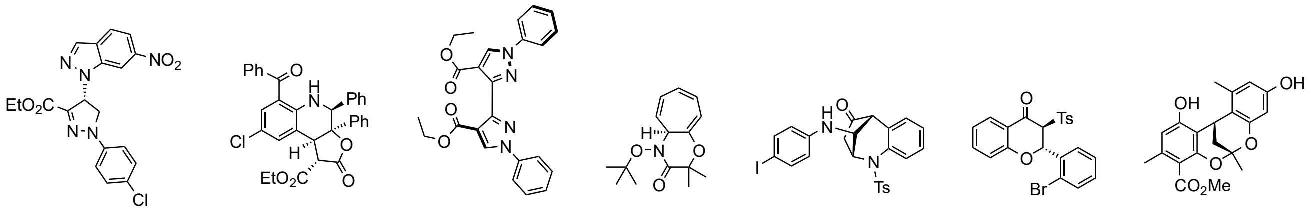

SUDVAR SUFYEA SUGNAM SUHCOQ SUHFEJ SUHKOY SULHOZ

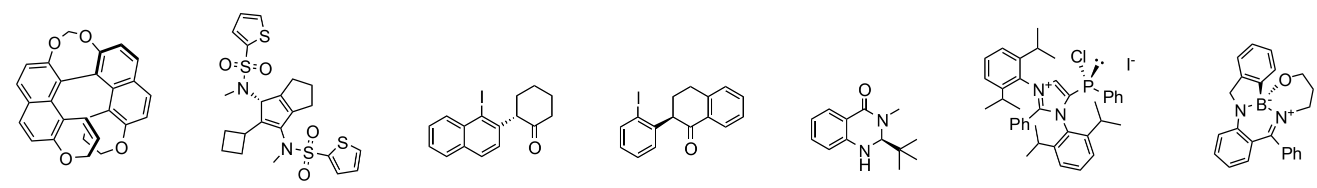

SUNKUK SUTMOM SUWKAZ SUWKON TACGUD TUCTAP TUDVAS

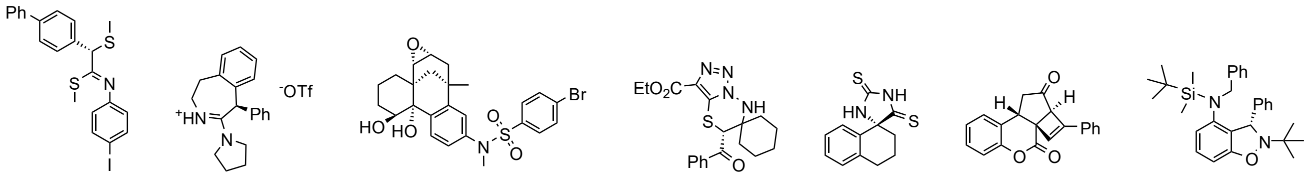

TUFLAK TUGVEZ TUHVEA TUXLAC UCEFIV UCUCOO UCUPAN

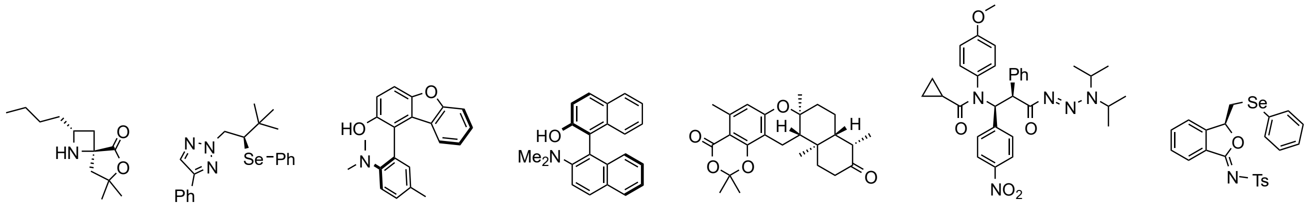

UGENOM01 UHABIR UHECOC UHECUI UKONAM ULACAO ULOSUM

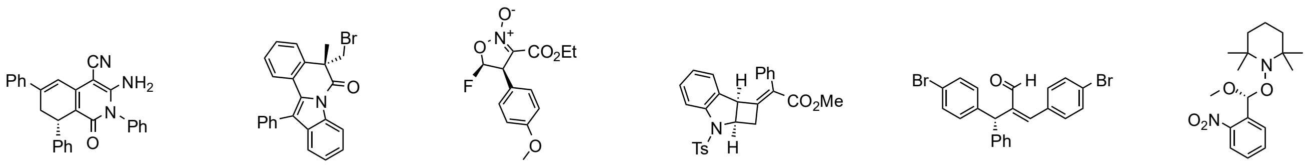

ULUTAZ UNOZEF UNUHIX UNUXOT UNUYAG01 UPOREZ

CSD Code	Space Group	a / Å	b / Å	c / Å	α / °	β / °	γ / °	Publication Year	Reference
**UQAHEC**	$P2_1$	9.8309	11.2489	10.0300	90	98.8630	90	2021	[238]
**URESAO**	$P3_2$	10.4373	10.4373	13.6316	90	90	120	2021	[239]
**URESOC**	$P1$	4.7441	9.5883	12.077	73.421	89.483	86.045	2021	[240]
**URINAN**	$P2_12_12_1$	7.6547	8.3977	21.0738	90	90	90	2021	[241]
**URUPAB**	$P2_12_12_1$	6.5441	8.977	28.197	90	90	90	2021	[242]
**USAFOM**	$P2_12_12_1$	6.55	8.1588	36.3815	90	90	90	2021	[243]
**USIHOW**	$C2$	20.4479	12.8314	16.3557	90	103.791	90	2021	[244]
**USUVAI**	$P2_12_12_1$	7.2944	11.356	15.9573	90	90	90	2021	[245]
**USUVEM**	$P2_1$	5.3438	14.9294	8.1896	90	98.718	90	2021	[245]
**USUWEN**	$P2_1$	5.9952	16.775	8.7581	90	106.597	90	2021	[245]
**UTAXEV**	$P2_12_12_1$	7.4679	11.1943	19.428	90	90	90	2021	[246]
**UTEZOL**	$P2_12_12_1$	8.7643	10.729	15.1952	90	90	90	2021	[247]
**UTONAV**	$P2_12_12_1$	9.8748	13.7568	19.028	90	90	90	2021	[248]
**UTOZOV**	$P2_12_12_1$	8.8331	9.3709	20.538	90	90	90	2021	[249]
**UWAWOH**	$P2_12_12_1$	8.6116	10.0205	14.119	90	90	90	2021	[250]
**UWOBUG**	$P2_1$	10.535	7.8133	10.777	90	101.52	90	2021	[251]
**UXIBIP**	$P2_12_12_1$	5.3543	10.9069	21.8346	90	90	90	2021	[252]
**UYUGON**	$P6_5$	23.8314	23.8314	7.7204	90	90	120	2020	[253]
**VAMWIT**	$P2_1$	6.0097	15.4823	13.245	90	101.648	90	2021	[254]
**VOZTIQ**	$P2_12_12_1$	8.0742	15.3584	18.0688	90	90	90	2020	[255]
**VUCHOT**	$P2_12_12_1$	9.691	11.0221	12.1283	90	90	90	2020	[256]
**VUFPIY**	$P4_3$	7.53409	7.53409	42.4001	90	90	90	2020	[257]
**VUHCUZ01**	$P2_1$	8.783	9.4326	15.8299	90	95.56	90	2020	[258]
**VUHPIA**	$P2_1$	12.2582	8.3935	14.2842	90	115.353	90	2020	[259]
**VUKYUY**	$P2_12_12_1$	8.9476	10.1905	15.587	90	90	90	2020	[260]
**VULREC**	$P2_12_12_1$	7.9495	9.8378	15.6228	90	90	90	2020	[261]
**VURPUW**	$P2_12_12_1$	9.9959	13.7259	16.9959	90	90	90	2020	[262]
**VUSTIP**	$P2_1$	10.547	9.089	10.569	90	117.789	90	2020	[263]
**VUWNUZ**	$P2_12_12_1$	10.1848	16.7586	20.4119	90	90	90	2020	[264]
**VUZWIZ**	$P2_1$	7.5964	5.974	17.6654	90	100.711	90	2020	[265]
**WALVUE01**	$P2_1$	5.556	16.76	10.12	90	90.089	90	2021	[266]
**WOSMID**	$P2_12_12_1$	9.51	12.0903	12.876	90	90	90	2020	[267]
**WUFKAM**	$P2_12_12_1$	6.4986	9.3449	16.9901	90	90	90	2020	[268]
**WUFLAN**	$P2_12_12_1$	5.8786	9.1357	26.6603	90	90	90	2020	[268]
**WUMRAA**	$P6_1$	24.902	24.902	5.852	90	90	120	2020	[269]
**WUNZEN**	$P2_12_12_1$	9.5993	10.1443	22.025	90	90	90	2020	[270]
**WUPXEN**	$P2_1$	11.065	7.5047	18.2302	90	96.753	90	2020	[271]
**WUWDOK**	$P2_12_12_1$	10.368	11.776	32.459	90	90	90	2020	[272]
**WUXDOL**	$P2_12_12_1$	9.903	12.9337	20.9573	90	90	90	2020	[273]
**XAGDUI**	$P2_12_12_1$	5.8636	12.121	21.7943	90	90	90	2020	[274]
**XAGFAQ**	$P2_12_12_1$	5.7367	13.2513	16.5649	90	90	90	2020	[275]
**XAGGAR**	$P2_12_12_1$	5.6348	13.4906	16.7334	90	90	90	2020	[275]
**XAKMIJ**	$P2_1$	9.9964	7.977	11.5581	90	113.947	90	2020	[276]
**XOFRAO01**	$P1$	7.9413	7.9555	8.0755	81.685	63.54	72.764	2020	[277]
**XOLNIY01**	$P2_1$	10.5292	7.9342	12.7336	90	113.133	90	2020	[278]
**XUBWEZ**	$P2_1$	8.2183	16.769	9.1367	90	103.2	90	2020	[279]
**XUGWEE**	$P2_1$	5.8236	16.7277	10.5963	90	96.022	90	2020	[280]
**XURCOF**	$P2_1212$	13.9949	23.7478	7.051	90	90	90	2020	[281]
**XURWIT**	$P2_12_12_1$	8.8686	10.3542	18.9662	90	90	90	2020	[282]
**XUWWIY**	$P2_1$	8.1235	9.9488	11.8624	90	103.849	90	2020	[283]
**YAGNON**	$P2_12_12_1$	8.2058	12.3378	17.8903	90	90	90	2021	[284]

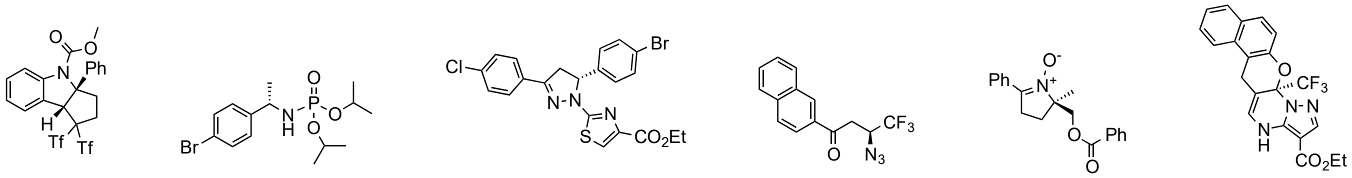

UQAHEC URESAO URESOC URINAN URUPAB USAFOM

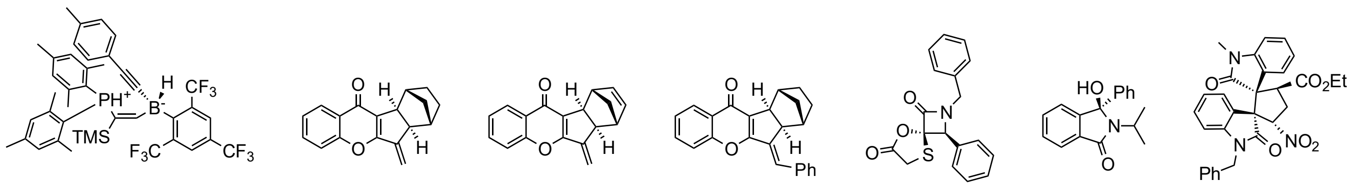

USIHOW USUVAI USUVEM USUWEN UTAXEV UTEZOL UTONAV

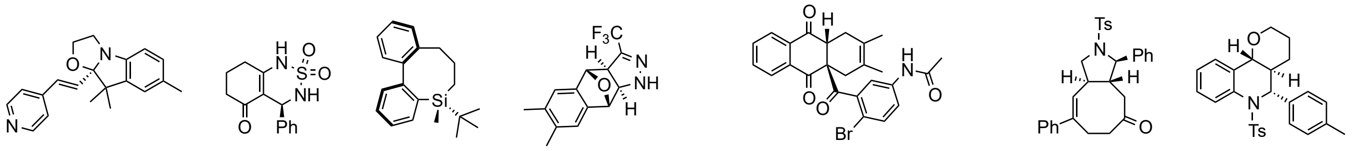

UTOZOV UWAWOH UWOBUG UXIBIP UYUGON VAMWIT VOZTIQ

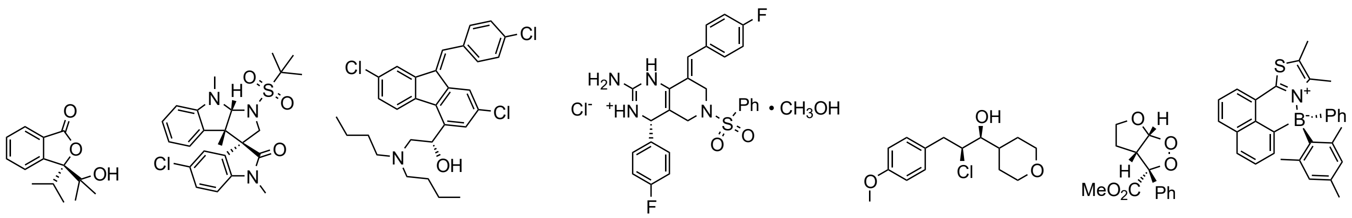

VUCHOT VUFPIY VUHCUZ01 VUHPIA VUKYUY VULREC VURPUW

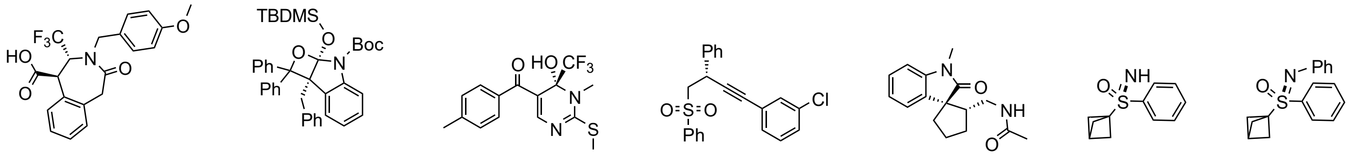

VUSTIP VUWNUZ VUZWIZ WALVUE01 WOSMID WUFKAM WUFLAN

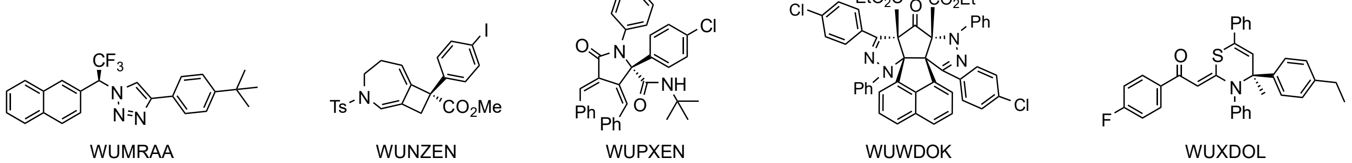

WUMRAA WUNZEN WUPXEN WUWDOK WUXDOL

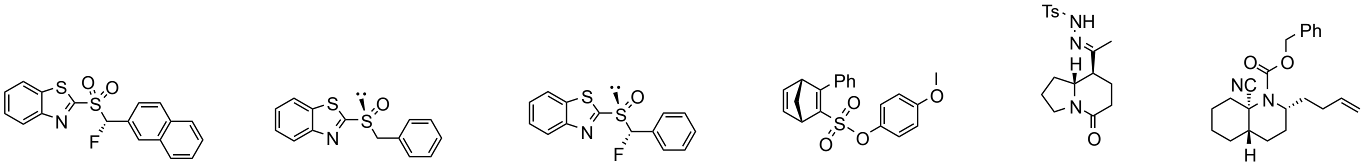

XAGDUI XAGFAQ XAGGAR XAKMIJ XOFRAO01 XOLNIY01

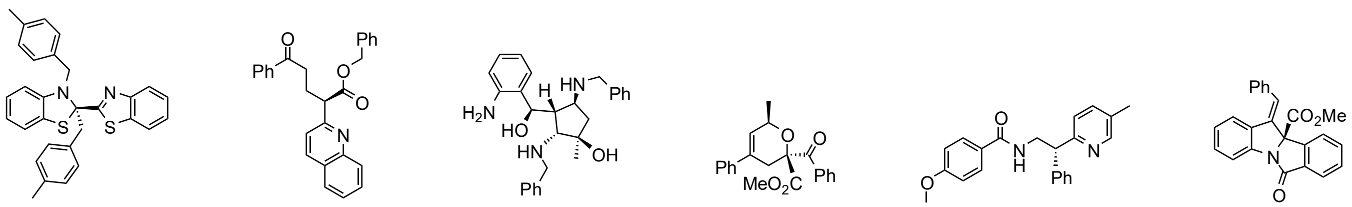

XUBWEZ XUGWEE XURCOF XURWIT XUWWIY YAGNON

CSD Code	Space Group	a / Å	b / Å	c / Å	α / °	β / °	γ / °	Publication Year	Reference
**YAGYUE**	$P2_1$	4.8515	21.804	11.5794	90	91.942	90	2021	[285]
**YAHXAK**	$P2_12_12_1$	10.3372	10.4523	14.5189	90	90	90	2021	[286]
**YALREM**	$P4_3$	10.7269	10.7269	14.5422	90	90	90	2021	[287]
**YAMSAK**	$P2_12_12_1$	8.79	12.01	21.15	90	90	90	2021	[288]
**YUKBEO**	$P2_12_12_1$	8.7556	12.3993	12.7861	90	90	90	2020	[289]
**YUKNIE**	$P2_1$	7.816	15.143	10.494	90	91.3	90	2020	[290]
**YUPZOB**	$P2_12_12_1$	5.521	8.801	30.85	90	90	90	2020	[199]
**YUPZUH**	$P2_12_12_1$	5.4469	8.8456	31.639	90	90	90	2020	[199]
**YUQBAQ**	$P2_1$	8.8108	5.9955	15.7023	90	97.468	90	2020	[199]
**YUQBEU**	$P2_12_12_1$	5.5067	8.7964	33.354	90	90	90	2020	[199]
**ZOYMUY**	$C2$	22.3238	6.7591	19.0609	90	122.823	90	2020	[291]
**ZUBVEA**	$P2_1$	9.919	7.2183	13.0454	90	94.293	90	2020	[292]
**ZUCRIB**	$C2$	27.1253	6.6649	12.131	90	119.012	90	2020	[293]
**ZUJTIK**	$P2_12_12_1$	10.6405	14.2884	18.1199	90	90	90	2020	[294]
**ZUJTOQ**	$P2_12_12_1$	10.641	14.2764	18.0395	90	90	90	2020	[294]
**ZUJVOS**	$P2_12_12_1$	8.7648	10.4987	12.6415	90	90	90	2020	[295]
**ZUNXUE**	$I2$	13.406	7.0933	16.704	90	95.72	90	2020	[296]
**ZUVLEK**	$P6_1$	8.6843	8.6843	32.226	90	90	120	2020	[297]
**ZUWJIN**	$P2_12_12_1$	6.0357	39.665	10.0021	90	90	90	2020	[298]
**ZUWPEP**	$P2_12_12_1$	7.9612	11.1841	19.4292	90	90	90	2020	[299]

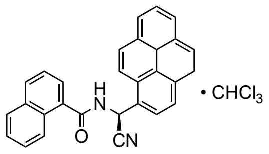
YAGYUE

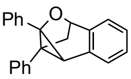
YAHXAK

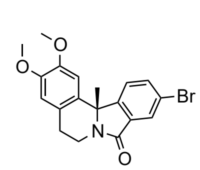
YALREM

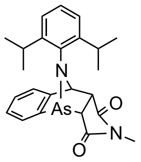
YAMSAK

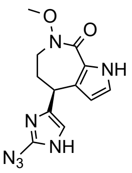
YUKBEO

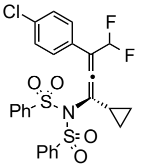
YUKNIE

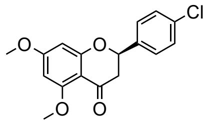
YUPZOB

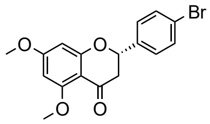
YUPZUH

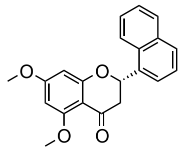
YUQBAQ

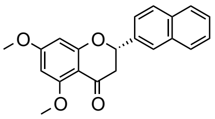
YUQBEU

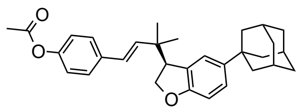
ZOYMUY

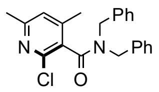
ZUBVEA

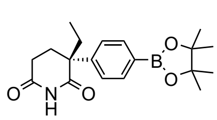
ZUCRIB

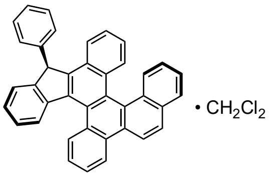
ZUJTIK

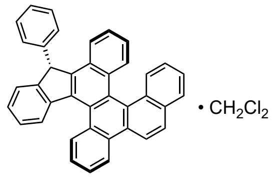
ZUJTOQ

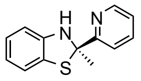
ZUJVOS

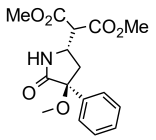
ZUNXUE

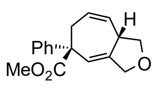
ZUVLEK

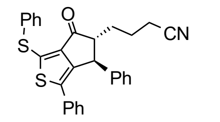
ZUWJIN

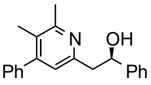
ZUWPEP

Conglomerate crystals which undergo racemic twinning

CSD Code	Space Group	a / Å	b / Å	c / Å	α / °	β / °	γ / °	Publication Year	Reference
**FAKSOD**	$P2_12_12_1$	9.0147	10.9941	11.1906	90	90	90	2020	[300]
**FUFDUI**	$P4_3$	8.574	8.574	29.928	90	90	90	2020	[301]
**GUHBIX**	$P2_1$	6.2909	12.7266	9.6707	90	90.04	90	2020	[302]
**GUZMUM**	$P2_1$	11.226	6.4276	11.494	90	99.141	90	2020	[303]
**HALQAQ**	$P2_12_12_1$	4.9552	15.4018	20.3511	90	90	90	2021	[304]
**IPEHET**	$P2_1$	8.5942	18.759	9.1396	90	100.374	90	2021	[305]
**IPIWUC**	$I4_1$	21.432	21.432	8.9094	90	90	90	2021	[306]
**IQUJEM01**	$P2_12_12_1$	5.4317	9.7084	26.7011	90	90	90	2021	[307]
**JUPTAS**	$P2_1$	9.3553	6.1653	16.682	90	105.942	90	2020	[308]
**KUPTOH**	$P2_12_12_1$	15.5188	21.4989	7.5658	90	90	90	2020	[309]
**MUDYUI**	$P2_1$	8.353	5.8263	10.678	90	109.178	90	2020	[146]
**MUSHUG**	$P2_12_12_1$	8.9464	10.2403	13.9027	90	90	90	2020	[310]
**OXACUO**	$P2_12_12_1$	10.3933	17.413	20.363	90	90	90	2021	[179]
**OYUXUE**	$P2_12_12_1$	6.0151	9.2249	39.209	90	90	90	2020	[311]
**OYUYAL**	$P2_1212$	41.158	9.2846	6.0202	90	90	90	2020	[311]
**SABKEP**	$P2_12_12_1$	8.0553	10.2611	10.6161	90	90	90	2020	[312]
**SILZEU01**	$P2_12_12_1$	7.9339	15.9616	17.3578	90	90	90	2020	[313]
**UCACEK**	$P2_12_12_1$	5.992	7.871	36.074	90	90	90	2021	[314]
**UJILIL**	$P2_12_12_1$	8.0621	9.2391	16.1087	90	90	90	2020	[315]
**ULEJAZ**	$P2_12_12_1$	8.0941	17.5936	32.602	90	90	90	2021	[316]
**UZESIE**	$P2_12_12_1$	5.9754	15.824	22.222	90	90	90	2021	[317]
**XAGDIW**	$P2_12_12_1$	5.6585	13.5586	17.3178	90	90	90	2020	[274]

References

[1] J. Li, H. Y. Zhou, Y. Han, C. F. Chen, *Angew. Chem. Int. Ed.* **2021**, *60*, 21927–21933. https://doi.org/10.1002/anie.202108209.

[2] B. Singh, S. K. Bankar, K. Kumar, S. S. V. Ramasastry, *Chem. Sci.* **2020**, *11*, 4948–4953. https://doi.org/10.1039/d0sc01932a.

[3] W. Chen, A. Paul, K. A. Abboud, D. Seidel, *Nat. Chem.* **2020**, *12*, 545–550. https://doi.org/10.1038/s41557-020-0438-z.

[4] H. Liu, D. G. Xia, Z. W. Chu, R. Hu, X. Cheng, X. H. Lv, *Bioorg. Chem.* **2020**, *100*, 103907. https://doi.org/10.1016/j.bioorg.2020.103907.

[5] A. S. Carlson, A. M. Petre, J. J. Topczewski, *Tetrahedron Lett.* **2021**, *67*, 6044–6059. https://doi.org/10.1016/j.tetlet.2021.152860.

[6] Q. F. Bao, M. Li, Y. Xia, Y. Z. Wang, Z. Z. Zhou, Y. M. Liang, *Org. Lett.* **2021**, *23*, 1107–1112. https://doi.org/10.1021/acs.orglett.1c00034.

[7] Q. F. Bao, M. Li, Y. Xia, Y. Z. Wang, Z. Z. Zhou, Y. M. Liang, *Org. Lett.* **2021**, *23*, 1107–1112. https://doi.org/10.1021/acs.orglett.1c00034.

[8] K. G. Ghosh, D. Das, P. Chandu, D. Sureshkumar, *J. Org. Chem.* **2021**, *86*, 2644–2657. https://doi.org/10.1021/acs.joc.0c02695.

[9] H. Wu, X. Li, Z. Yan, N. Ma, S. Song, G. Zhang, N. Jiao, *Org. Lett.* **2021**, *23*, 762–766. https://doi.org/10.1021/acs.orglett.0c03991.

[10] S. Sathiyamoorthi, A. I. Almansour, S. K. Raju, A. Natarajan, R. R. Kumar, *Synth. Commun.* **2021**, *51*, 234–244. https://doi.org/10.1080/00397911.2020.1821226.

[11] H. Zheng, R. Wang, K. Wang, D. Wherritt, H. Arman, M. P. Doyle, *Chem. Sci.* **2021**, *12*, 4819–4824. https://doi.org/10.1039/d1sc00158b.

[12] K. Kodama, S. Yamaguchi, S. Hayano, T. Houkawa, Y. Kitahara, H. Shitara, T. Hirose, *Tetrahedron* **2021**, *87*, 132082. https://doi.org/10.1016/j.tet.2021.132082.

[13] H. Huang, T. Zhang, J. Sun, *Angew. Chem. Int. Ed.* **2021**, *60*, 2668–2673. https://doi.org/10.1002/anie.202013062.

[14] M. N. Dimukhametov, V. F. Mironov, D. R. Islamov, I. A. Litvinov, O. I. Gnezdilov, Y. V. Danilova, *Mendeleev Commun.* **2021**, *31*, 107–109. https://doi.org/10.1016/j.mencom.2021.01.033.

[15] V. García-Vázquez, L. Hoteite, C. P. Lakeland, D. W. Watson, J. P. A. Harrity, *Org. Lett.* **2021**, *23*, 2811–2815. https://doi.org/10.1021/acs.orglett.1c00752.

[16] E. Merkulova, A. V. Kolobov, K. L. Ovchinnikov, V. N. Khrustalev, V. G. Nenajdenko, *Chem. Heterocycl. Compd.* **2021**, *57*, 245–252. https://doi.org/10.1007/s10593-021-02900-y.

[17] S. Song, Y. Li, D. Y. Chen, X. P. Wang, Y. L. Liu, L. Y. Chen, *ChemistrySelect* **2021**, *6*, 3187–3191. https://doi.org/10.1002/slct.202100578.

[18] A. R. Claus, T. A. C. Goulart, D. F. Back, G. Zeni, *Eur. J. Org. Chem.* **2021**, *2021*, 2180–2187. https://doi.org/10.1002/ejoc.202100176.

[19] C. Y. Zhang, J. Zhu, S. H. Cui, X. Y. Xie, X. D. Wang, L. Wu, *Org. Lett.* **2021**, *23*, 3530–3535. https://doi.org/10.1021/acs.orglett.1c00943.

[20] P. Praveena, B. K. Sarojini, Q. A. Wong, C. K. Quah, T. Vishwanath, *J. Mol. Struct.* **2021**, *1239*, 130524. https://doi.org/10.1016/j.molstruc.2021.130524.

[21] A. Tsurusaki, H. Shimatani, K. Kamikawa, *Asian J. Org. Chem.* **2021**, *10*, 154–159. https://doi.org/10.1002/ajoc.202000669.

[22] X. Cheng, B. G. Cai, H. Mao, J. Lu, L. Li, K. Wang, J. Xuan, *Org. Lett.* **2021**, *23*, 4109–4114. https://doi.org/10.1021/acs.orglett.1c00979.

[23] J. O. Strelnikova, A. N. Koronatov, N. V. Rostovskii, A. F. Khlebnikov, O. V. Khoroshilova, M. A. Kryukova, M. S. Novikov, *Org. Lett.* **2021**, *23*, 4173–4178. https://doi.org/10.1021/acs.orglett.1c01092.

[24] K. Zhang, Q. Zhang, D. Wei, R. Tian, Z. Duan, *Org. Chem. Front.* **2021**, *8*, 3740–3745. https://doi.org/10.1039/d1qo00535a.

[25] Z. Xu, L. Fu, X. Fang, B. Huang, L. Zhou, J. P. Wan, *Org. Lett.* **2021**, *23*, 5049–5053.

https://doi.org/10.1021/acs.orglett.1c01581.

[26] M. Kamlar, E. Henriksson, I. Císařová, M. Malo, H. Sundén, *J. Org. Chem.* **2021**, *86*, 8660–8671. https://doi.org/10.1021/acs.joc.1c00445.

[27] E. P. Kramarova, A. D. Volodin, V. V. Negrebetsky, A. D. Shagina, T. M. Aliev, P. V. Dorovatovskii, R. A. Novikov, A. V. Vologzhanina, A. A. Korlyukov, *Molecules* **2021**, *26*, 3548. https://doi.org/10.3390/molecules26123548.

[28] M. Zhu, X. L. Huang, H. Xu, X. Zhang, C. Zheng, S. L. You, *CCS Chem.* **2021**, *3*, 652–664. https://doi.org/10.31635/ccschem.020.202000254.

[29] M. Zhu, X. L. Huang, S. Sun, C. Zheng, S. L. You, *J. Am. Chem. Soc.* **2021**, *143*, 13441–13449. https://doi.org/10.1021/jacs.1c07082.

[30] W. Wu, X. Han, Z. Weng, *Org. Chem. Front.* **2020**, *7*, 3499–3504. https://doi.org/10.1039/d0qo00955e.

[31] M. Albrecht, J. Zhang, J. Li, J. S. Ward, K. N. Truong, K. Rissanen, *J. Org. Chem.* **2020**, *85*, 12160–12174. https://doi.org/10.1021/acs.joc.0c01373.

[32] H. E. Ho, A. Pagano, J. A. Rossi-Ashton, J. R. Donald, R. G. Epton, J. C. Churchill, M. J. James, P. O'Brien, R. J. K. Taylor, W. P. Unsworth, *Chem. Sci.* **2020**, *11*, 1353–1360. https://doi.org/10.1039/c9sc05311e.

[33] N. Li, L. Tu, G. Cheng, H. Sa, Z. Li, T. Feng, Y. Zheng, J. Liu, *Tetrahedron Lett.* **2020**, *61*, 151579. https://doi.org/10.1016/j.tetlet.2019.151579.

[34] R. Das, N. P. Khot, A. S. Deshpande, M. Kapur, *Chem. Eur. J.* **2020**, *26*, 927–938. https://doi.org/10.1002/chem.201904512.

[35] M. M. Xia, L. Le Song, F. X. Li, Y. N. Hou, Z. F. Shi, X. P. Cao, *Adv. Synth. Catal.* **2020**, *362*, 601–608. https://doi.org/10.1002/adsc.201901214.

[36] J. Shen, R. Ye, A. Romanies, A. Roy, F. Chen, C. Ren, Z. Liu, H. Zeng, *J. Am. Chem. Soc.* **2020**, *142*, 10050–10058. https://doi.org/10.1021/jacs.0c02013.

[37] B. Ganesan, K. Govindan, G. C. Senadi, M. Kandasamy, W. Y. Lin, W. Y. Lin, W. Y. Lin, *Chem. Commun.* **2020**, *56*, 6488–6491. https://doi.org/10.1039/d0cc03033c.

[38] L. Carreras, A. Franconetti, A. Grabulosa, A. Frontera, A. Vidal-Ferran, *Org. Chem. Front.* **2020**, *7*, 1626–1634. https://doi.org/10.1039/d0qo00416b.

[39] P. E. Reyes-Gutiérrez, T. T. Amatov, P. Švec, I. Císařová, D. Šaman, R. Pohl, F. Teplý, L. Pospíšil, *Chempluschem* **2020**, *85*, 2212–2218. https://doi.org/10.1002/cplu.202000151.

[40] G. P. Junor, J. Lorkowski, C. M. Weinstein, R. Jazzar, C. Pietraszuk, G. Bertrand, *Angew. Chem. Int. Ed.* **2020**, *59*, 22028–22033. https://doi.org/10.1002/anie.202010744.

[41] A. Milián, P. Garciá-Garciá, A. Pérez-Redondo, R. Sanz, J. J. Vaquero, M. A. Fernández-Rodríguez, *Org. Lett.* **2020**, *22*, 8464–8469. https://doi.org/10.1021/acs.orglett.0c03067.

[42] S. M. Kim, O. Y. Kang, H. J. Lim, S. J. Park, *ACS Omega* **2020**, *5*, 10191–10199. https://doi.org/10.1021/acsomega.0c01086.

[43] D. Han, J. Yi, C. Liu, L. Liang, K. Huang, L. Jing, D. Qin, *Spectrochim. Acta - Part A Mol. Biomol. Spectrosc.* **2020**, *238*, 118405. https://doi.org/10.1016/j.saa.2020.118405.

[44] S. H. Bennett, A. Fawcett, E. H. Denton, T. Biberger, V. Fasano, N. Winter, V. K. Aggarwal, *J. Am. Chem. Soc.* **2020**, *142*, 16766–16775. https://doi.org/10.1021/jacs.0c07357.

[45] S. P. Singh, S. Tripathi, A. Yadav, R. Kant, H. K. Srivastava, A. K. Srivastava, *Chem. Commun.* **2020**, *56*, 12789–12792. https://doi.org/10.1039/d0cc04415f.

[46] K. Ishijima, S. Tanaka, H. Imoto, K. Naka, *Dalt. Trans.* **2020**, *49*, 15612–15621. https://doi.org/10.1039/d0dt02669g.

[47] L. M. Gronbach, A. Voss, M. Frahm, A. Villinger, J. Bresien, D. Michalik, M. Brasholz, *Org. Lett.* **2021**, *23*, 7834–7838. https://doi.org/10.1021/acs.orglett.1c02857.

[48] A. Washio, M. Hosaka, N. Uemura, Y. Yoshida, T. Mino, Y. Kasashima, M. Sakamoto, *Cryst. Growth Des.* **2021**, *21*, 2423–2428. https://doi.org/10.1021/acs.cgd.1c00036.

[49] Q. Liu, D. Yang, T. Chen, B. Zhang, C. Xing, L. Zhang, Y. Lu, G. Du, *Cryst. Growth Des.* **2021**, *21*, 6321–6331. https://doi.org/10.1021/acs.cgd.1c00785.

[50] M. Liu, L. Zhou, W. Shi, Y. Hu, J. Liao, Z. Duan, W. Wang, Y. Wu, B. Zheng, H. Guo, *Org. Lett.* **2021**, *23*, 7703–7707. https://doi.org/10.1021/acs.orglett.1c02557.

[51] G. Y. Zhu, T. Y. Zhai, X. Li, C. Y. Shi, X. Q. Zhu, L. W. Ye, *Org. Lett.* **2021**, *23*, 8067–8071.

https://doi.org/10.1021/acs.orglett.1c03092.
[52] D. Reiter, P. Frisch, D. Wendel, F. M. Hörmann, S. Inoue, *Dalt. Trans.* **2020**, *49*, 7060–7068. https://doi.org/10.1039/d0dt01522a.
[53] W. Dai, C. Li, Y. Liu, X. Han, X. Li, K. Chen, H. Liu, *Org. Chem. Front.* **2020**, *7*, 2612–2617. https://doi.org/10.1039/d0qo00320d.
[54] P. T. Tang, Y. X. Shao, L. N. Wang, Y. Wei, M. Li, N. J. Zhang, X. P. Luo, Z. Ke, Y. J. Liu, M. H. Zeng, M. H. Zeng, *Chem. Commun.* **2020**, *56*, 6680–6683. https://doi.org/10.1039/c9cc10080f.
[55] M. A. Novikov, A. Y. Bobrova, I. A. Mezentsev, M. G. Medvedev, Y. V. Tomilov, *J. Org. Chem.* **2020**, *85*, 6295–6308. https://doi.org/10.1021/acs.joc.9b03445.
[56] D. Dhara, P. K. Pal, R. Dolai, N. Chrysochos, H. Rawat, B. J. Elvers, I. Krummenacher, H. Braunschweig, C. Schulzke, V. Chandrasekhar, U. D. Priyakumar, A. Jana, *Chem. Commun.* **2021**, *57*, 9546–9549. https://doi.org/10.1039/d1cc04421d.
[57] K. Kiyokawa, K. Jou, S. Minakata, *Chem. Eur. J.* **2021**, *27*, 13971–13976. https://doi.org/10.1002/chem.202102635.
[58] M. V. Goryaeva, S. O. Kushch, Y. V. Burgart, V. I. Saloutin, *Russ. Chem. Bull.* **2020**, *69*, 2163–2166. https://doi.org/10.1007/s11172-020-3016-z.
[59] G. Valenti, P. Tinnemans, I. Baglai, W. L. Noorduin, B. Kaptein, M. Leeman, J. H. ter Horst, R. M. Kellogg, *Angew. Chem. Int. Ed.* **2021**, *60*, 5279–5282. https://doi.org/10.1002/anie.202013502.
[60] M. Zhu, H. Xu, X. Zhang, C. Zheng, S. L. You, *Angew. Chem. Int. Ed.* **2021**, *60*, 7036–7040. https://doi.org/10.1002/anie.202016899.
[61] R. Chitra, R. R. Choudhury, F. Capet, P. Roussel, P. Bhatt, *J. Mol. Struct.* **2021**, *1234*, 130142. https://doi.org/10.1016/j.molstruc.2021.130142.
[62] E. A. Nikiforova, D. V. Baibarodskikh, N. F. Kirillov, M. V. Dmitriev, D. P. Zverev, *Russ. J. Org. Chem.* **2020**, *56*, 2074–2078. https://doi.org/10.1134/S1070428020120040.
[63] Y. M. Sadykova, A. V. Zalaltdinova, A. K. Smailov, L. M. Trofimova, J. K. Voronina, A. R. Burilov, M. A. Pudovik, *Chem. Heterocycl. Compd.* **2020**, *56*, 1605–1610. https://doi.org/10.1007/s10593-020-02856-5.
[64] Y. Cui, F. Li, X. Zhang, *Chem. Commun.* **2021**, *57*, 3275–3278. https://doi.org/10.1039/d1cc00165e.
[65] R. Ye, J. Sun, Y. Han, C. G. Yan, *New J. Chem.* **2021**, *45*, 5075–5080. https://doi.org/10.1039/d0nj06036d.
[66] G. Q. Xu, T. F. Xiao, G. X. Feng, C. Liu, B. Zhang, P. F. Xu, *Org. Lett.* **2021**, *23*, 2846–2852. https://doi.org/10.1021/acs.orglett.1c00226.
[67] P. A. Zagorska, L. Grigorjeva, J. Bolsakova, *Chem. Heterocycl. Compd.* **2021**, *57*, 159–165. https://doi.org/10.1007/s10593-021-02888-5.
[68] J. R. Frost, C. B. Cheong, W. M. Akhtar, D. F. J. Caputo, K. E. Christensen, N. G. Stevenson, T. J. Donohoe, *Tetrahedron* **2021**, *86*, 132051. https://doi.org/10.1016/j.tet.2021.132051.
[69] Z. J. Zhang, X. Zhou, D. Li, Y. Chen, W. W. Xiao, R. T. Li, L. D. Shao, *J. Org. Chem.* **2021**, *86*, 7609–7624. https://doi.org/10.1021/acs.joc.1c00656.
[70] H. Kinouchi, K. Sugimoto, Y. Yamaoka, H. Takikawa, K. Takasu, *J. Org. Chem.* **2021**, *86*, 12615–12622. https://doi.org/10.1021/acs.joc.1c01108.
[71] G. S. Chen, Y. B. Fang, Z. Ren, X. Tian, Y. L. Liu, *Org. Chem. Front.* **2021**, *8*, 3820–3828. https://doi.org/10.1039/d1qo00575h.
[72] B. Zhu, H. Han, W. K. Su, B. Yan, Z. Li, C. Yu, X. Jiang, *Org. Lett.* **2021**, *23*, 4488–4492. https://doi.org/10.1021/acs.orglett.1c01441.
[73] X. Kong, F. Yu, Z. Chen, F. Gong, S. Yang, J. Liu, B. Luo, X. Fang, *Sci. China Chem.* **2021**, *64*, 991–998. https://doi.org/10.1007/s11426-021-9972-3.
[74] P. Wang, R. Yu, S. Ali, Z. Wang, Z. Liu, J. Gao, H. Zheng, *Molecules* **2021**, *26*, 2974. https://doi.org/10.3390/molecules26102974.
[75] Y. X. Cao, P. Silalai, C. F. Liu, K. Y. Yu, X. Bao, X. H. Zhao, R. Saeeng, C. A. Fan, *Chem. Eur. J.* **2021**, *27*, 8473–8478. https://doi.org/10.1002/chem.202100762.
[76] M. M. D. Roy, S. R. Baird, E. Dornsiepen, L. A. Paul, L. Miao, M. J. Ferguson, Y. Zhou, I. Siewert, E. Rivard, *Chem. Eur. J.* **2021**, *27*, 8572–8579. https://doi.org/10.1002/chem.202100969.
[77] D. Wu, S. S. Cui, F. Bian, W. Yu, *Org. Lett.* **2021**, *23*, 6057–6061. https://doi.org/10.1021/acs.orglett.1c02133.

[78] J. S. Martin, X. Zeng, X. Chen, C. Miller, C. Han, Y. Lin, N. Yamamoto, X. Wang, S. Yazdi, Y. Yan, M. C. Beard, Y. Yan, *J. Am. Chem. Soc.* **2021**, *143*, 11361–11369. https://doi.org/10.1021/jacs.1c00503.

[79] P. Anaikutti, P. Makam, *Bioorg. Chem.* **2020**, *105*, 104379. https://doi.org/10.1016/j.bioorg.2020.104379.

[80] Z. Cui, K. Zhang, L. Gu, Z. Bu, J. Zhao, Q. Wang, *Chem. Commun.* **2021**, *57*, 9402–9405. https://doi.org/10.1039/d1cc03478b.

[81] Z. Zhu, C. M. Glinkerman, D. L. Boger, *J. Am. Chem. Soc.* **2020**, *142*, 20778–20787. https://doi.org/10.1021/jacs.0c09775.

[82] A. Kondoh, M. Terada, *Chem. Eur. J.* **2021**, *27*, 585–588. https://doi.org/10.1002/chem.202002943.

[83] Z. H. Luan, J. P. Qu, Y. B. Kang, *J. Am. Chem. Soc.* **2020**, *142*, 20942–20947. https://doi.org/10.1021/jacs.0c10707.

[84] N. Kise, S. Yamamoto, T. Sakurai, *J. Org. Chem.* **2020**, *85*, 13973–13982. https://doi.org/10.1021/acs.joc.0c02000.

[85] Y. Te Liu, C. W. Cheng, H. C. Lu, T. Y. Chang, C. Y. Chen, H. C. Yang, S. H. Yu, S. Zehra, S. H. Liu, M. K. Leung, K. M. Lee, H. H. Chen, *J. Org. Chem.* **2020**, *85*, 13655–13663. https://doi.org/10.1021/acs.joc.0c01833.

[86] H. Choi, H. J. Shirley, H. R. M. Aitken, T. Schulte, T. Söhnel, P. A. Hume, M. A. Brimble, D. P. Furkert, *Org. Lett.* **2020**, *22*, 1022–1027. https://doi.org/10.1021/acs.orglett.9b04567.

[87] A. R. Chadeayne, D. N. K. Pham, J. A. Golen, D. R. Manke, *Acta Cryst. E* **2020**, *76*, 1201–1205. https://doi.org/10.1107/S2056989020008683.

[88] E. I. Linkova, V. S. Grinev, O. A. Mayorova, A. Y. Yegorova, *IUCrData* **2020**, *5*, x200919-. https://doi.org/10.1107/S2414314620009190/HB4353ISUP3.CML.

[89] L. Singla, H. R. Yadav, A. R. Choudhury, *Acta Crystallogr. Sect. B Struct. Sci. Cryst. Eng. Mater.* **2020**, *76*, 604–617. https://doi.org/10.1107/S2052520620006873.

[90] D. Pechalrieu, D. Dauzonne, P. B. Arimondo, M. Lopez, *Eur. J. Med. Chem.* **2020**, *186*, 111829. https://doi.org/10.1016/j.ejmech.2019.111829.

[91] T. V. Omelian, A. V. Dobrydnev, O. Y. Utchenko, E. N. Ostapchuk, I. S. Konovalova, Y. M. Volovenko, *Monatshefte fur Chemie* **2020**, *151*, 1759–1772. https://doi.org/10.1007/S00706-020-02694-3/FIGURES/7.

[92] W. E. Noland, H. V. Kumar, Y. Reddi, C. J. Cramer, A. V. Novikov, H. Kim, Y. Zhu, Y. C. Chin, Y. Zhou, P. Radakovic, A. Uprety, J. Xie, G. C. Flick, *J. Org. Chem.* **2020**, *85*, 5265–5287. https://doi.org/10.1021/acs.joc.9b03363.

[93] R. Liu, M. Yang, W. Xie, W. Dong, H. Zhou, S. Yadav, V. I. Potkin, G. Qiu, *J. Org. Chem.* **2020**, *85*, 5312–5320. https://doi.org/10.1021/acs.joc.9b03466.

[94] Y. C. Wang, Z. Fang, K. K. Huang, G. Qiu, J. B. Liu, *Mol. Catal.* **2020**, *495*, 111163. https://doi.org/10.1016/j.mcat.2020.111163.

[95] Y. A. Zhang, Z. Ding, P. Liu, W. S. Guo, L. R. Wen, M. Li, *Org. Chem. Front.* **2020**, *7*, 1321–1326. https://doi.org/10.1039/d0qo00300j.

[96] Z. Kuang, H. Chen, J. Qiu, Z. Ou, Y. Lan, Q. Song, *Chem* **2020**, *6*, 2347–2363. https://doi.org/10.1016/j.chempr.2020.06.034.

[97] J. L. Liu, J. L. Tu, F. Liu, *Org. Lett.* **2020**, *22*, 7369–7372. https://doi.org/10.1021/acs.orglett.0c02742.

[98] Y. Mu, Y. Yuan, Y. Wang, M. Xu, Y. Feng, Y. Zhao, Y. Li, *Org. Biomol. Chem.* **2020**, *18*, 6916–6926. https://doi.org/10.1039/d0ob01626h.

[99] F. Wang, Q. Zhou, X. Zhang, X. Fan, *J. Org. Chem.* **2021**, *86*, 11708–11722. https://doi.org/10.1021/acs.joc.1c01198.

[100] T. Suárez-Rodríguez, Á. L. Suárez-Sobrino, A. Ballesteros, *Chem. – A Eur. J.* **2021**, *27*, 13079–13084. https://doi.org/10.1002/CHEM.202102534.

[101] C. Chen, C. G. Daniliuc, G. Kehr, G. Erker, *Angew. Chem. Int. Ed.* **2021**, *60*, 19905–19911. https://doi.org/10.1002/anie.202106724.

[102] T. Wang, Y. Liu, J. Xu, *Synth. Commun.* **2020**, *50*, 315–321. https://doi.org/10.1080/00397911.2017.1402348.

[103] A. Mbodji, G. Gbabode, M. Sanselme, Y. Cartigny, N. Couvrat, M. Leeman, V. Dupray, R. M. Kellogg, G. Coquerel, *Cryst. Growth Des.* **2020**, *20*, 2562–2569. https://doi.org/10.1021/acs.cgd.9b01699.

[104] D. Liu, J. Sun, J. Xie, H. Shi, C. G. Yan, *J. Org. Chem.* **2021**, *86*, 1827–1842. https://doi.org/10.1021/acs.joc.0c02645.

[105] G. Perumal, M. Kandasamy, B. Ganesan, K. Govindan, H. Sathya, M. Y. Hung, G. Chandru Senadi, Y. C. Wu, W.

Y. Lin, *Tetrahedron* **2021**, *80*, 131891. https://doi.org/10.1016/j.tet.2020.131891.

[106] Z. J. Niu, L. H. Li, X. S. Li, H. C. Liu, W. Y. Shi, Y. M. Liang, *Org. Lett.* **2021**, *23*, 1315–1320. https://doi.org/10.1021/acs.orglett.0c04300.

[107] B. Hernández, P. Narea, J. Cisterna, L. Maxwell, A. Cárdenas, I. Brito, G. E. Delgado, *J. Mol. Struct.* **2021**, *1245*, 226–232. https://doi.org/10.1016/j.molstruc.2021.131070.

[108] B. Su, Y. Hou, L. Wang, X. Li, D. Pan, T. Yan, A. Zhang, F. Paison, L. Ding, *Curr. Org. Synth.* **2019**, *16*, 1174–1184. https://doi.org/10.2174/1570179416666191022113022.

[109] S. D. Schnell, M. Schilling, J. Sklyaruk, A. Linden, S. Luber, K. Gademann, *Org. Lett.* **2021**, *23*, 2426–2430. https://doi.org/10.1021/acs.orglett.0c04113.

[110] K. Dong, C. Y. Zhao, X. J. Wang, L. Z. Wu, Q. Liu, *Org. Lett.* **2021**, *23*, 2816–2820. https://doi.org/10.1021/acs.orglett.1c00762.

[111] A. V. Serebryannikova, E. E. Galenko, M. S. Novikov, A. F. Khlebnikov, *Tetrahedron* **2021**, *88*, 132153. https://doi.org/10.1016/j.tet.2021.132153.

[112] C. Fu, S. P. Kelley, J. Tu, M. Harmata, *J. Org. Chem.* **2021**, *86*, 7028–7037. https://doi.org/10.1021/acs.joc.1c00032.

[113] Y. Dong, A. T. Wrobel, G. J. Porter, J. J. Kim, J. Z. Essman, S. L. Zheng, T. A. Betley, *J. Am. Chem. Soc.* **2021**, *143*, 7480–7489. https://doi.org/10.1021/jacs.1c02074.

[114] D. P. Hari, R. Madhavachary, V. Fasano, J. Haire, V. K. Aggarwal, *J. Am. Chem. Soc.* **2021**, *143*, 7462–7470. https://doi.org/10.1021/jacs.1c01966.

[115] G. Zhang, A. Yu, Y. Lei, X. Meng, L. Zhang, *Org. Chem. Front.* **2021**, *8*, 3718–3723. https://doi.org/10.1039/d1qo00312g.

[116] Y. Wang, Z. Lin, J. C. A. Oliveira, L. Ackermann, *J. Org. Chem.* **2021**, *86*, 15935–15945. https://doi.org/10.1021/acs.joc.1c00682.

[117] F. Chen, Y. Shao, M. Li, C. Yang, S. J. Su, H. Jiang, Z. Ke, W. Zeng, *Nat. Commun.* **2021**, *12*, 1–9. https://doi.org/10.1038/s41467-021-23326-2.

[118] S. K. Kushvaha, M. Francis, J. Kumar, E. Nag, P. Ravichandran, S. Roy, K. Chandra Mondal, *RSC Adv.* **2021**, *11*, 22849–22858. https://doi.org/10.1039/d1ra03071j.

[119] W. Riley, A. C. Jones, K. Singh, D. L. Browne, A. M. Stuart, *Chem. Commun.* **2021**, *57*, 7406–7409. https://doi.org/10.1039/d1cc02587b.

[120] A. Jończyk, J. K. Maurin, M. Moreń, A. Kowalkowska, *ChemistrySelect* **2021**, *6*, 5575–5585. https://doi.org/10.1002/slct.202100787.

[121] R. H. Dai, L. Han, Q. Wang, S. K. Tian, *Chem. Commun.* **2021**, *57*, 8449–8451. https://doi.org/10.1039/d1cc03141d.

[122] A. Liu, T. Guo, S. Zhang, H. Yang, Q. Zhang, Y. Chai, S. Zhang, *Org. Lett.* **2021**, *23*, 6326–6331. https://doi.org/10.1021/acs.orglett.1c02128.

[123] J. X. Chen, P. D. Jadhav, C. N. Chen, R. S. Liu, *Org. Lett.* **2021**, *23*, 6246–6251. https://doi.org/10.1021/acs.orglett.1c01987.

[124] X. Tao, K. Škoch, C. G. Daniliuc, G. Kehr, G. Erker, *Chem. Sci.* **2020**, *11*, 1542–1548. https://doi.org/10.1039/c9sc03870a.

[125] I. Suzuki, Y. Sakamoto, Y. Seo, Y. Ninomaru, K. Tokuda, I. Shibata, *J. Org. Chem.* **2020**, *85*, 2759–2769. https://doi.org/10.1021/acs.joc.9b03055.

[126] P. Urbanovský, J. Kotek, I. Císařová, P. Hermann, *RSC Adv.* **2020**, *10*, 21329–21349. https://doi.org/10.1039/d0ra03075a.

[127] L. Cao, H. Zhao, Z. Tan, R. Guan, H. Jiang, M. Zhang, *Org. Lett.* **2020**, *22*, 4781–4785. https://doi.org/10.1021/acs.orglett.0c01580.

[128] T. Hu, Y. Xu, S. Zhang, H. Y. Xiong, G. Zhang, *Org. Lett.* **2020**, *22*, 8866–8871. https://doi.org/10.1021/acs.orglett.0c03239.

[129] C. S. Lopez, R. Sanz, C. Feberero, C. Sedano, S. Suarez-Pantiga, *J. Org. Chem.* **2020**, *85*, 12561–12578. https://doi.org/10.1021/acs.joc.0c01732.

[130] C. te Grotenhuis, J. T. Mattos, A. T. Radosevich, *Phosphorus, Sulfur Silicon Relat. Elem.* **2020**, *195*, 940–946. https://doi.org/10.1080/10426507.2020.1804188.

[131] V. Tiwari, J. T. Bingham, S. Vyas, A. Singh, *Org. Biomol. Chem.* **2020**, *18*, 9044–9049. https://doi.org/10.1039/d0ob01697g.

[132] S. Wübbolt, C. B. Cheong, J. R. Frost, K. E. Christensen, T. J. Donohoe, *Angew. Chemie* **2020**, *132*, 11435–11440. https://doi.org/10.1002/ange.202003614.

[133] K. Terashima, T. Kawasaki-Takasuka, T. Agou, T. Kubota, T. Yamazaki, *Chem. Commun.* **2020**, *56*, 3031–3034. https://doi.org/10.1039/c9cc08936e.

[134] X. Liu, H. Song, X. Zhai, C. H. Tung, W. Wang, *Org. Biomol. Chem.* **2020**, *18*, 1572–1576. https://doi.org/10.1039/d0ob00037j.

[135] S. Jin, H. T. Dang, G. C. Haug, V. D. Nguyen, H. D. Arman, O. V. Larionov, *Chem. Sci.* **2020**, *11*, 9101–9108. https://doi.org/10.1039/d0sc03118f.

[136] T. Ito, N. Iwasawa, J. Takaya, *Angew. Chem. Int. Ed.* **2020**, *59*, 11913–11917. https://doi.org/10.1002/anie.202004444.

[137] T. R. Penjarla, M. Kundarapu, K. Rangan, A. Bhattacharya, *Org. Biomol. Chem.* **2020**, *18*, 9623–9631. https://doi.org/10.1039/d0ob01903h.

[138] I. Escofet, H. Armengol-Relats, H. Bruss, M. Besora, A. M. Echavarren, *Chem. Eur. J.* **2020**, *26*, 15738–15745. https://doi.org/10.1002/chem.202004237.

[139] M. M. Efremova, A. P. Molchanov, A. S. Novikov, G. L. Starova, A. A. Muryleva, A. V. Slita, V. V. Zarubaev, *Tetrahedron* **2020**, *76*, 131104. https://doi.org/10.1016/j.tet.2020.131104.

[140] N. Uemura, M. Hosaka, A. Washio, Y. Yoshida, T. Mino, M. Sakamoto, *Cryst. Growth Des.* **2020**, *20*, 4898–4903. https://doi.org/10.1021/acs.cgd.0c00829.

[141] J. C. Jiménez-Cruz, R. Guzmán-Mejía, E. Juaristi, O. Sánchez-Antonio, M. A. García-Revilla, J. B. González-Campos, J. Aviña-Verduzco, *New J. Chem.* **2020**, *44*, 13382–13392. https://doi.org/10.1039/d0nj02630a.

[142] M. Ojha, M. Anitha, R. K. Bansal, *J. Mol. Struct.* **2021**, *1233*, 130103. https://doi.org/10.1016/j.molstruc.2021.130103.

[143] V. Shcherbakova, D. Dibchak, M. Snisarenko, Y. Skalenko, A. V. Denisenko, A. S. Kuznetsova, P. K. Mykhailiuk, *J. Org. Chem.* **2021**, *86*, 2200–2209. https://doi.org/10.1021/acs.joc.0c02355.

[144] S. Ben Salah, M. Sanselme, Y. Champavier, M. Othman, A. Daïch, I. Chataigner, A. Martin Lawson, *Eur. J. Org. Chem.* **2021**, *2021*, 102–116. https://doi.org/10.1002/ejoc.202001404.

[145] S. Yang, Y. Chen, Z. Yuan, F. Bu, C. Jiang, Z. Ding, *Org. Biomol. Chem.* **2020**, *18*, 9873–9882. https://doi.org/10.1039/d0ob01987a.

[146] B. Xu, L. Troian-Gautier, R. Dykstra, R. T. Martin, O. Gutierrez, U. K. Tambar, *J. Am. Chem. Soc.* **2020**, *142*, 6206–6215. https://doi.org/10.1021/jacs.0c00147.

[147] A. Kharma, C. Jacob, Í. A. O. Bozzi, G. A. M. Jardim, A. L. Braga, K. Salomão, C. C. Gatto, M. F. S. Silva, C. Pessoa, M. Stangier, L. Ackermann, E. N. da Silva Júnior, *Eur. J. Org. Chem.* **2020**, *2020*, 4474–4486. https://doi.org/10.1002/ejoc.202000216.

[148] A. Schild, R. Bhardwaj, N. Wenger, D. Tscherrig, P. Kandasamy, J. Dernič, R. Baur, C. Peinelt, M. A. Hediger, M. Lochner, *Int. J. Mol. Sci.* **2020**, *21*, 1–28. https://doi.org/10.3390/ijms21165604.

[149] S. Boudriga, S. Haddad, V. Murugaiyah, M. Askri, M. Knorr, C. Strohmann, C. Golz, *Molecules* **2020**, *25*, 1963. https://doi.org/10.3390/molecules25081963.

[150] M. V. Grudova, D. M. Gil, V. N. Khrustalev, E. V. Nikitina, A. A. Sinelshchikova, M. S. Grigoriev, A. V. Kletskov, A. Frontera, F. I. Zubkov, *New J. Chem.* **2020**, *44*, 20167–20180. https://doi.org/10.1039/d0nj04328a.

[151] N. Uemura, S. Toyoda, W. Shimizu, Y. Yoshida, T. Mino, M. Sakamoto, *Symmetry* **2020**, *12*, 910. https://doi.org/10.3390/sym12060910.

[152] F. P. Zhu, X. Guo, F. M. Zhang, X. M. Zhang, H. Wang, Y. Q. Tu, *Org. Lett.* **2020**, *22*, 2076–2080. https://doi.org/10.1021/acs.orglett.0c00565.

[153] A. K. Sahu, R. Unnava, S. Shit, A. K. Saikia, *J. Org. Chem.* **2020**, *85*, 1961–1971. https://doi.org/10.1021/acs.joc.9b02686.

[154] M. D. Kosobokov, M. O. Zubkov, V. V. Levin, V. A. Kokorekin, A. D. Dilman, *Chem. Commun.* **2020**, *56*, 9453–9456. https://doi.org/10.1039/d0cc04617e.

[155] R. Calvo, A. Le Tellier, T. Nauser, D. Rombach, D. Nater, D. Katayev, *Angew. Chem. Int. Ed.* **2020**, *59*, 17162–17168. https://doi.org/10.1002/anie.202005720.

[156] D. Chen, W. Du, X. Yang, T. Liu, *J. Org. Chem.* **2020**, *85*, 9088–9095. https://doi.org/10.1021/acs.joc.0c01006.

[157] Y. S. Rao, D. S. Latha, N. Devunuri, A. I. Almansour, N. Arumugam, S. Yaragorla, *Eur. J. Org. Chem.* **2020**, *2020*, 4134–4145. https://doi.org/10.1002/ejoc.202000511.

[158] H. Li, J. Wu, J. Zheng, W. D. Z. Li, *Chem. Commun.* **2021**, *57*, 11092–11095. https://doi.org/10.1039/d1cc04576h.

[159] T. Nakamura, K. Ban, Y. Yoshida, T. Mino, Y. Kasashima, M. Sakamoto, *Chem. Eur. J.* **2021**, *27*, 16338–16341. https://doi.org/10.1002/chem.202103345.

[160] K. Sanada, A. Washio, K. Nishihata, F. Yagishita, Y. Yoshida, T. Mino, S. Suzuki, Y. Kasashima, M. Sakamoto, *Cryst. Growth Des.* **2021**, *21*, 6051–6055. https://doi.org/10.1021/acs.cgd.1c01010.

[161] T. Mashiko, Y. Shingai, J. Sakai, S. Kamo, S. Adachi, A. Matsuzawa, K. Sugita, *Angew. Chem. Int. Ed.* **2021**, *60*, 24484–24487. https://doi.org/10.1002/anie.202110556.

[162] M. Guillot, J. de Meester, S. Huynen, L. Collard, K. Robeyns, O. Riant, T. Leyssens, *Angew. Chem. Int. Ed.* **2020**, *59*, 11303–11306. https://doi.org/10.1002/anie.202002464.

[163] Z. H. Wang, L. W. Shen, K. X. Xie, Y. You, J. Q. Zhao, W. C. Yuan, *Org. Lett.* **2020**, *22*, 3114–3118. https://doi.org/10.1021/acs.orglett.0c00874.

[164] J. Yang, Y. Xia, *Chem. Sci.* **2021**, *12*, 4389–4394. https://doi.org/10.1039/d1sc00001b.

[165] P. Karishma, A. Gogia, S. K. Mandal, R. Sakhuja, *Adv. Synth. Catal.* **2021**, *363*, 762–775. https://doi.org/10.1002/adsc.202001146.

[166] J. Zhang, Z. Xie, *Chem. Sci.* **2021**, *12*, 5616–5620. https://doi.org/10.1039/d0sc07047e.

[167] J. L. Tyler, A. Noble, V. K. Aggarwal, *Angew. Chem. Int. Ed.* **2021**, *60*, 11824–11829. https://doi.org/10.1002/anie.202102754.

[168] W. X. Wei, Y. Li, Y. T. Wen, M. Li, X. S. Li, C. T. Wang, H. C. Liu, Y. Xia, B. S. Zhang, R. Q. Jiao, Y. M. Liang, *J. Am. Chem. Soc.* **2021**, *143*, 7868–7875. https://doi.org/10.1021/jacs.1c04114.

[169] D. P. Gerasimova, A. F. Saifina, D. V. Zakharychev, R. R. Fayzullin, A. R. Kurbangalieva, O. A. Lodochnikova, *CrystEngComm* **2021**, *23*, 3907–3918. https://doi.org/10.1039/d1ce00227a.

[170] A. S. Zalivatskaya, A. A. Golovanov, I. A. Boyarskaya, M. A. Kruykova, O. V. Khoroshilova, A. V. Vasilyev, *Eur. J. Org. Chem.* **2021**, *2021*, 2634–2649. https://doi.org/10.1002/ejoc.202100280.

[171] J. K. Huang, K. S. Shia, *Org. Lett.* **2021**, *23*, 5709–5713. https://doi.org/10.1021/acs.orglett.1c01807.

[172] M. N. Elinson, A. N. Vereshchagin, Y. E. Ryzhkova, F. V. Ryzhkov, A. N. Fakhrutdinov, M. P. Egorov, *Electrochem* **2021**, *2*, 295–310. https://doi.org/10.3390/electrochem2020021.

[173] J. C. Bristow, S. V. A. Cliff, S. Yang, J. D. Wallis, *CrystEngComm* **2021**, *23*, 4500–4512. https://doi.org/10.1039/d1ce00377a.

[174] C. P. Surya, G. Harichandran, S. Muthu, *J. Mol. Struct.* **2021**, *1242*, 130861. https://doi.org/10.1016/j.molstruc.2021.130861.

[175] S. S. Niu, Y. Cheng, *Org. Chem. Front.* **2021**, *8*, 4192–4201. https://doi.org/10.1039/d1qo00483b.

[176] T. A. Bartholome, J. J. Martinez, A. Kaur, D. J. D. Wilson, J. L. Dutton, C. D. Martin, *Organometallics* **2021**, *40*, 1966–1973. https://doi.org/10.1021/acs.organomet.1c00261.

[177] A. Lebrêne, T. Martzel, L. Gouriou, M. Sanselme, V. Levacher, S. Oudeyer, C. Afonso, C. Loutelier-Bourhis, J. F. Brière, *J. Org. Chem.* **2021**, *86*, 8600–8609. https://doi.org/10.1021/acs.joc.1c00252.

[178] K. M. Lambert, J. B. Cox, L. Liu, A. C. Jackson, S. Yruegas, K. B. Wiberg, J. L. Wood, *Angew. Chem. Int. Ed.* **2020**, *59*, 9757–9766. https://doi.org/10.1002/anie.202003829.

[179] M. Fischer, C. Hering-Junghans, *Chem. Sci.* **2021**, *12*, 10279–10289. https://doi.org/10.1039/d1sc02947a.

[180] Y. Wang, Z. Qi, Y. Niu, H. Feng, E. Benassi, B. Qian, *Chem. Commun.* **2021**, *57*, 7533–7536. https://doi.org/10.1039/d1cc02517a.

[181] V. Kremláček, E. Kertész, Z. Benkő, M. Erben, R. Jirásko, A. Růžička, R. Jambor, L. Dostál, *Chem. Eur. J.* **2021**, *27*, 13149–13160. https://doi.org/10.1002/chem.202101686.

[182] Y. Zhao, D. Mandal, J. Guo, Y. Wu, D. W. Stephan, *Chem. Commun.* **2021**, *57*, 7758–7761. https://doi.org/10.1039/d1cc03048e.

[183] Y. Tao, X. Hao, L. Jing, L. Sun, S. Cherukupalli, S. Liu, G. Wu, S. Xu, X. Zhang, X. Shi, Y. Song, X. Liu, P. Zhan, *Bioorg. Chem.* **2021**, *115*, 105254. https://doi.org/10.1016/j.bioorg.2021.105254.

[184] H. Zheng, K. Wang, I. Faghihi, W. P. Griffith, H. Arman, M. P. Doyle, *ACS Catal.* **2021**, *11*, 9869–9874. https://doi.org/10.1021/acscatal.1c02674.

[185] J. L. Wu, W. H. Chiou, *J. Org. Chem.* **2020**, *85*, 9051–9063. https://doi.org/10.1021/acs.joc.0c00964.

[186] M. Wang, M. Zhang, Y. Luo, Z. Liu, C. Yang, J. Lan, D. Wu, J. You, *Org. Lett.* **2020**, *22*, 135–139. https://doi.org/10.1021/acs.orglett.9b04046.

[187] H. Zhou, R. Wang, H. Zhang, W. Chen, X. B. Lu, *Org. Biomol. Chem.* **2020**, *18*, 905–911. https://doi.org/10.1039/c9ob02398d.

[188] L. Seijo, P. Ondet, S. Olivero, E. Duñach, *New J. Chem.* **2020**, *44*, 10479–10483. https://doi.org/10.1039/d0nj01857k.

[189] K. Tanaka, Y. Iwama, M. Kishimoto, N. Ohtsuka, Y. Hoshino, K. Honda, K. Tanaka, Y. Hoshino, K. Honda, *Org. Lett.* **2020**, *22*, 5207–5211. https://doi.org/10.1021/acs.orglett.0c01852.

[190] M. Scherübl, C. G. Daniliuc, A. Studer, *Angew. Chem. Int. Ed.* **2021**, *60*, 711–715. https://doi.org/10.1002/anie.202012654.

[191] D. T. Yang, J. Zheng, J. B. Peng, X. Wang, S. Wang, *J. Org. Chem.* **2021**, *86*, 829–836. https://doi.org/10.1021/acs.joc.0c02379.

[192] W. Li, M. De Groen, H. J. M. Kramer, R. De Gelder, P. Tinnemans, H. Meekes, J. H. Ter Horst, *Cryst. Growth Des.* **2021**, *21*, 112–124. https://doi.org/10.1021/acs.cgd.0c00890.

[193] R. Y. Zhang, F. Jin, X. G. Bao, H. Y. Li, X. P. Xu, S. J. Ji, *J. Org. Chem.* **2021**, *86*, 1141–1151. https://doi.org/10.1021/acs.joc.0c02610.

[194] J. Li, C. G. Daniliuc, G. Kehr, G. Erker, *Eur. J. Inorg. Chem.* **2020**, *2020*, 1096–1100. https://doi.org/10.1002/ejic.201901085.

[195] J. Ma, F. Schäfers, C. Daniliuc, K. Bergander, C. A. Strassert, F. Glorius, *Angew. Chem. Int. Ed.* **2020**, *59*, 9639–9645. https://doi.org/10.1002/anie.202001200.

[196] A. El-Harairy, M. Shaheen, J. Li, Y. Wu, M. Li, Y. Gu, *RSC Adv.* **2020**, *10*, 13507–13516. https://doi.org/10.1039/d0ra00990c.

[197] A. Prajapati, M. Kumar, R. Thakuria, A. K. Basak, *Tetrahedron Lett.* **2020**, *61*, 152347. https://doi.org/10.1016/j.tetlet.2020.152347.

[198] O. Shalimov, E. Rusanov, O. Muzychka, P. Onys’ko, *Molecules* **2020**, *25*, 2887. https://doi.org/10.3390/molecules25122887.

[199] W. Shimizu, N. Uemura, Y. Yoshida, T. Mino, Y. Kasashima, M. Sakamoto, *Cryst. Growth Des.* **2020**, *20*, 5676–5681. https://doi.org/10.1021/acs.cgd.0c00955.

[200] W. Bin Cao, S. Li, M. M. Xu, H. Li, X. P. Xu, Y. Lan, S. J. Ji, *Angew. Chem. Int. Ed.* **2020**, *59*, 21425–21430. https://doi.org/10.1002/anie.202008110.

[201] S. Dong, C. G. Daniliuc, G. Kehr, G. Erker, *Chem. Eur. J.* **2020**, *26*, 745–753. https://doi.org/10.1002/chem.201904919.

[202] A. A. Moroz, V. E. Zhulanov, M. V. Dmitriev, A. N. Maslivets, *Tetrahedron* **2020**, *76*, 130880. https://doi.org/10.1016/j.tet.2019.130880.

[203] D. M. Patel, H. J. Patel, J. M. Padrón, H. M. Patel, *RSC Adv.* **2020**, *10*, 19600–19609. https://doi.org/10.1039/d0ra02990d.

[204] J. Fung, T. V. Duong, K. C. A. Braceros, M. L. Brooks, K. Schloesser-Lingscheit, T. K. S. Tagawa, J. M. Wilson, K. K. Jones, E. J. Valente, *J. Chem. Crystallogr.* **2021**, *51*, 14–41. https://doi.org/10.1007/s10870-020-00829-2.

[205] B. François, L. Eberlin, F. Berrée, A. Whiting, B. Carboni, *Eur. J. Org. Chem.* **2020**, *2020*, 3282–3293. https://doi.org/10.1002/ejoc.202000330.

[206] M. Eddahmi, N. M. M. Moura, L. Bouissane, O. Amiri, M. A. F. Faustino, J. A. S. Cavaleiro, R. F. Mendes, F. A. A. Paz, M. G. P. M. S. Neves, E. M. Rakib, *Molecules* **2020**, *25*, 126. https://doi.org/10.3390/molecules25010126.

[207] X. Zhang, M. Feng, G. Yang, Z. Chai, *J. Org. Chem.* **2020**, *85*, 430–440. https://doi.org/10.1021/acs.joc.9b02444.

[208] K. M. Dawood, A. Z. A. Elassar, O. A. Al-Fulaij, *J. Heterocycl. Chem.* **2020**, *57*, 370–376. https://doi.org/10.1002/jhet.3787.

[209] G. Force, A. Pérot, R. Guillot, V. Gandon, D. Leboeuf, *Synthesis* **2020**, *52*, 553–564. https://doi.org/10.1055/s-0039-1690745.

[210] S. Wang, R. Guillot, J. F. Carpentier, Y. Sarazin, C. Bour, V. Gandon, D. Lebœuf, *Angew. Chem. Int. Ed.* **2020**, *59*, 1134–1138. https://doi.org/10.1002/anie.201911761.

[211] M. Y. Chang, Y. L. Tsai, Y. L. Chang, *J. Org. Chem.* **2020**, *85*, 1033–1043.

https://doi.org/10.1021/acs.joc.9b02980.

[212] L. Huo, C. Dong, M. Wang, X. Lu, W. Zhang, B. Yang, Y. Yuan, S. Qiu, H. Liu, H. Tan, *Org. Lett.* **2020**, *22*, 934–938. https://doi.org/10.1021/acs.orglett.9b04486.

[213] S. B. Kamble, P. J. Maliekal, P. D. Dharpure, P. M. Badani, A. V. Karnik, *J. Org. Chem.* **2020**, *85*, 7739–7747. https://doi.org/10.1021/acs.joc.0c00363.

[214] W. Zang, Y. Wei, M. Shi, *Org. Lett.* **2020**, *22*, 5466–5472. https://doi.org/10.1021/acs.orglett.0c01819.

[215] H. L. Bian, S. Z. Tang, M. E. Chen, X. M. Zhang, J. W. Lv, X. W. Chen, F. M. Qi, S. W. Chen, F. M. Zhang, *Org. Lett.* **2020**, *22*, 5314–5319. https://doi.org/10.1021/acs.orglett.0c01466.

[216] A. Méndez, J. R. Valdez-Camacho, J. Escalante, *Molecules* **2020**, *25*, 5008. https://doi.org/10.3390/molecules25215008.

[217] A. Merschel, D. Rottschäfer, B. Neumann, H. G. Stammler, R. S. Ghadwal, *ACS Appl. Mater. Interfaces* **2020**, DOI 10.1021/acs.organomet.0c00045. https://doi.org/10.1021/acs.organomet.0c00045.

[218] M. E. Shirbhate, S. Kwon, A. Song, S. Kim, D. Kim, H. Huang, Y. Kim, H. Lee, S. J. Kim, M. H. Baik, J. Yoon, K. M. Kim, *ACS Appl. Mater. Interfaces* **2020**, DOI 10.1021/jacs.9b13232. https://doi.org/10.1021/jacs.9b13232.

[219] A. Baralle, T. Inukai, T. Yanagi, K. Nogi, A. Osuka, A. Nagaki, J. I. Yoshida, H. Yorimitsu, *Chem. Lett.* **2020**, *49*, 160–163. https://doi.org/10.1246/cl.190831.

[220] H. Zhang, M. Riomet, A. Roller, N. Maulide, *Org. Lett.* **2020**, *22*, 2376–2380. https://doi.org/10.1021/acs.orglett.0c00571.

[221] X. Chen, Y. Zhou, J. Jin, K. Farshadfar, A. Ariafard, W. Rao, P. W. H. Chan, *Adv. Synth. Catal.* **2020**, *362*, 1084–1095. https://doi.org/10.1002/adsc.201901263.

[222] K. L. V. Obydennov, T. A. Kalinina, O. A. Vysokova, P. A. Slepukhin, V. A. Pozdina, M. V. E. Ulitko, T. V. Glukhareva, *Acta Crystallogr. Sect. C Struct. Chem.* **2020**, *76*, 795–809. https://doi.org/10.1107/S2053229620009328.

[223] A. Ahmedova, G. Pavlović, M. Marinov, P. Marinova, G. Momekov, K. Paradowska, S. Yordanova, S. Stoyanov, N. Vassilev, N. Stoyanov, *Inorganica Chim. Acta* **2021**, *528*, 120605. https://doi.org/10.1016/j.ica.2021.120605.

[224] J. Fu, I. R. Tsapy Takia, P. Chen, W. Liu, C. Jiang, W. Yao, X. Zeng, Y. Wang, X. Han, *Org. Chem. Front.* **2021**, *8*, 6323–6329. https://doi.org/10.1039/d1qo01013a.

[225] Y. K. Jeon, W. S. Kim, *Org. Lett.* **2021**, *23*, 7545–7549. https://doi.org/10.1021/acs.orglett.1c02751.

[226] M. R. Becker, E. R. Wearing, C. S. Schindler, *Nat. Chem.* **2020**, *12*, 898–905. https://doi.org/10.1038/s41557-020-0541-1.

[227] L. L. Zhu, L. Tian, B. Cai, G. Liu, H. Zhang, Y. Wang, *Chem. Commun.* **2020**, *56*, 2979–2982. https://doi.org/10.1039/d0cc00601g.

[228] A. Purtsas, O. Kataeva, H. J. Knölker, *Chem. Eur. J.* **2020**, *26*, 2499–2508. https://doi.org/10.1002/chem.201905595.

[229] T. Mies, A. J. P. White, P. J. Parsons, A. G. M. Barrett, *J. Org. Chem.* **2021**, *86*, 1802–1817. https://doi.org/10.1021/acs.joc.0c02638.

[230] C. Wang, Z. Lai, H. Xie, S. Cui, *Angew. Chem. Int. Ed.* **2021**, *60*, 5147–5151. https://doi.org/10.1002/anie.202014686.

[231] J. Hua, M. Bian, T. Ma, M. Yang, W. He, Z. Yang, C. K. Liu, Z. Fang, K. Guo, *Catal. Sci. Technol.* **2021**, *11*, 2299–2305. https://doi.org/10.1039/d0cy02273j.

[232] S. Kansiz, A. Gul, N. Dege, E. Agar, E. Saif, *Acta Cryst. E* **2021**, *77*, 1087–1090. https://doi.org/10.1107/S2056989021010215.

[233] Z. J. Shen, B. Huang, N. Ma, L. Yao, C. Yang, L. Guo, W. Xia, *Adv. Synth. Catal.* **2021**, *363*, 1944–1954. https://doi.org/10.1002/adsc.202001583.

[234] A. Sperga, A. Kazia, J. Veliks, *Org. Biomol. Chem.* **2021**, *19*, 2688–2691. https://doi.org/10.1039/d1ob00270h.

[235] N. Chen, T. Zhou, H. Zhang, Y. Zhu, M. Lang, J. Wang, S. Peng, *J. Org. Chem.* **2021**, *86*, 4714–4732. https://doi.org/10.1021/acs.joc.1c00104.

[236] N. Q. Jiang, H. Y. Li, Z. J. Cai, S. J. Ji, *Org. Chem. Front.* **2021**, *8*, 5369–5376. https://doi.org/10.1039/d1qo00769f.

[237] Y. Li, Y. Wang, T. Yang, Z. Lin, X. Jiang, *Green Chem.* **2021**, *23*, 2986–2991. https://doi.org/10.1039/d0gc04407e.

[238] C. Lázaro-Milla, H. Yanai, P. Almendros, *Org. Lett.* **2021**, *23*, 2921–2926. https://doi.org/10.1021/acs.orglett.1c00557.

[239] V. Dašková, J. Buter, A. K. Schoonen, M. Lutz, F. de Vries, B. L. Feringa, *Angew. Chem. Int. Ed.* **2021**, *60*, 11120–

11126. https://doi.org/10.1002/anie.202014955.
[240] M. N. Ahmed, M. Madni, S. Anjum, S. Andleeb, S. Hameed, A. M. Khan, M. Ashfaq, M. N. Tahir, D. M. Gil, A. Frontera, *CrystEngComm* **2021**, *23*, 3276–3287. https://doi.org/10.1039/d1ce00256b.
[241] Y. Tu, H. Dong, H. Wang, Y. Ao, Y. Liu, *Chem. Commun.* **2021**, *57*, 4524–4527. https://doi.org/10.1039/d1cc00985k.
[242] J. Chen, Y. P. Zhu, J. H. Li, Q. A. Wang, *Chem. Commun.* **2021**, *57*, 5215–5218. https://doi.org/10.1039/d1cc01565f.
[243] V. Osyanin, D. V. Osipov, K. S. Korzhenko, O. P. Demidov, Y. N. Klimochkin, *Chem. Heterocycl. Compd.* **2021**, *57*, 305–313. https://doi.org/10.1007/s10593-021-02908-4.
[244] K. Škoch, C. G. Daniliuc, G. Kehr, G. Erker, *Angew. Chem. Int. Ed.* **2021**, *60*, 6757–6763. https://doi.org/10.1002/anie.202014562.
[245] W.-Q. Zhu, Z.-W. Zhang, W.-Y. Han, Y.-C. Fang, P. Yang, L.-Q. Li, Y.-Z. Chen, *Org. Chem. Front.* **2021**, *8*, 3413–3420. https://doi.org/10.1039/d1qo00458a.
[246] D. Narula, S. S. Bari, P. Yadav, S. Khullar, S. K. Mandal, G. Kaur, G. R. Chaudhary, A. Bhalla, *ChemistrySelect* **2021**, *6*, 3932–3940. https://doi.org/10.1002/slct.202101104.
[247] M. Sakamoto, N. Uemura, R. Saito, H. Shimobayashi, Y. Yoshida, T. Mino, T. Omatsu, *Angew. Chem. Int. Ed.* **2021**, *60*, 12819–12823. https://doi.org/10.1002/anie.202103382.
[248] C. Siva Sankara, I. N. N. Namboothiri, *Org. Lett.* **2021**, *23*, 1. https://doi.org/10.1021/acs.orglett.1c01360.
[249] Y. Aidibi, M. Allain, A. El-Ghayoury, P. Leriche, L. Sanguinet, *Dye. Pigment.* **2021**, *193*, 109476. https://doi.org/10.1016/j.dyepig.2021.109476.
[250] A. Bouzina, M. Berredjem, B. Belhani, S. Bouacida, C. Marminon, M. Le Borgne, Z. Bouaziz, M. Aissaoui, *Res. Chem. Intermed.* **2021**, *47*, 1359–1376. https://doi.org/10.1007/s11164-020-04378-3.
[251] X. Wang, H. R. Wang, X. Xu, D. Zhao, *Eur. J. Org. Chem.* **2021**, *2021*, 3039–3042. https://doi.org/10.1002/ejoc.202100535.
[252] T. Cao, Z. Yang, Y. Sun, N. Zhao, S. Lu, J. Zhang, L. Wang, *Eur. J. Org. Chem.* **2021**, *2021*, 2950–2954. https://doi.org/10.1002/ejoc.202100521.
[253] J. Beck, O. Fuhr, M. Nieger, S. Bräse, *R. Soc. Open Sci.* **2020**, *7*, 200626. https://doi.org/10.1098/rsos.200626.
[254] J. Liu, Y. Zhou, J. Zhu, Z. X. Yu, *Org. Lett.* **2021**, *23*, 7566–7570. https://doi.org/10.1021/acs.orglett.1c02766.
[255] S. J. Gharpure, D. S. Vishwakarma, *Eur. J. Org. Chem.* **2020**, *2020*, 6887–6891. https://doi.org/10.1002/ejoc.201901598.
[256] N. Kise, S. Yamamoto, T. Sakurai, *Tetrahedron* **2020**, *76*, 130820. https://doi.org/10.1016/j.tet.2019.130820.
[257] S. Bhandari, S. Sana, V. Lahoti, R. Tokala, N. Shankaraiah, *RSC Adv.* **2020**, *10*, 16101–16109. https://doi.org/10.1039/d0ra00684j.
[258] M. B. de Freitas-Marques, M. I. Yoshida, C. Fernandes, B. L. Rodrigues, W. N. Mussel, *J. Struct. Chem.* **2020**, *61*, 151–159. https://doi.org/10.1134/S0022476620010175.
[259] Y. Sun, Z. F. Gao, W. Bin Yan, B. R. Yao, W. Y. Xin, C. H. Wang, Q. G. Meng, G. G. Hou, *Eur. J. Med. Chem.* **2020**, *198*, 112366. https://doi.org/10.1016/j.ejmech.2020.112366.
[260] S. Xu, H. M. Holst, S. B. McGuire, N. J. Race, *J. Am. Chem. Soc.* **2020**, *142*, 8090–8096. https://doi.org/10.1021/jacs.0c02095.
[261] S. Budde, F. Goerdeler, J. Floß, P. Kreitmeier, E. F. Hicks, O. Moscovitz, P. H. Seeberger, H. M. L. Davies, O. Reiser, *Org. Chem. Front.* **2020**, *7*, 1789–1795. https://doi.org/10.1039/d0qo00168f.
[262] Z. He, L. Liu, Z. Zhao, S. K. Mellerup, Y. Ge, X. Wang, N. Wang, S. Wang, *Chem. Eur. J.* **2020**, *26*, 12403–12410. https://doi.org/10.1002/chem.202000775.
[263] M. I. Adamovskyi, M. M. Avramenko, D. M. Volochnyuk, S. V. Ryabukhin, *ACS Omega* **2020**, *5*, 20932–20942. https://doi.org/10.1021/acsomega.0c02394.
[264] P. Franceschi, J. Mateos, A. Vega-Peñaloza, L. Dell'Amico, *Eur. J. Org. Chem.* **2020**, *2020*, 6718–6722. https://doi.org/10.1002/ejoc.202001057.
[265] V. P. Andrade, M. Mittersteiner, H. G. Bonacorso, M. A. P. Martins, N. Zanatta, *Eur. J. Org. Chem.* **2020**, *2020*, 5527–5536. https://doi.org/10.1002/ejoc.202000879.
[266] F. Wang, H. Zhou, Y. Li, H. Bao, *Org. Chem. Front.* **2021**, *8*, 1817–1822. https://doi.org/10.1039/d1qo00107h.
[267] M. S. Morales-Ríos, D. E. González-Juárez, G. Martínez-Gudiño, N. Pérez-Hernández, L. M. Del Razo, H. L.

Mendoza-Figueroa, J. B. García-Vázquez, *J. Mol. Struct.* **2020**, *1202*, 127267. https://doi.org/10.1016/j.molstruc.2019.127267.

[268] R. M. Bär, L. Langer, M. Nieger, S. Bräse, *Adv. Synth. Catal.* **2020**, *362*, 1356–1361. https://doi.org/10.1002/adsc.201901453.

[269] C. Liu, C. Zhu, Y. Cai, Z. Yang, H. Zeng, F. Chen, H. Jiang, *Chem. Eur. J.* **2020**, *26*, 1953–1957. https://doi.org/10.1002/chem.201905445.

[270] M. He, N. Chen, L. Liu, Y. Zhu, Q. Li, H. Li, M. Lang, J. Wang, S. Peng, *J. Org. Chem.* **2020**, *85*, 4418–4429. https://doi.org/10.1021/acs.joc.0c00149.

[271] Y. M. Yan, M. L. Wang, Y. L. Liu, Y. C. He, *Tetrahedron* **2020**, *76*, 131389. https://doi.org/10.1016/j.tet.2020.131389.

[272] I. Yavari, P. Ravaghi, M. Safaei, *Monatshefte fur Chemie* **2020**, *151*, 1121–1129. https://doi.org/10.1007/s00706-020-02638-x.

[273] Z. Luo, S. Bhavanarushi, A. Sreenivas, N. S. Reddy, A. Valeru, I. Khan, Y. Xu, B. Liu, J. Xie, *J. Heterocycl. Chem.* **2020**, *57*, 3334–3341. https://doi.org/10.1002/jhet.4049.

[274] N. Erdeljac, C. Mück-Lichtenfeld, C. G. Daniliuc, R. Gilmour, *Chem. Eur. J.* **2020**, *26*, 13704–13715. https://doi.org/10.1002/chem.202003361.

[275] N. Erdeljac, C. Mück-Lichtenfeld, C. G. Daniliuc, R. Gilmour, *Chem. Eur. J.* **2020**, *26*, 13704–13715. https://doi.org/10.1002/chem.202003361.

[276] C. J. Smedley, G. Li, A. S. Barrow, T. L. Gialelis, M. C. Giel, A. Ottonello, Y. Cheng, S. Kitamura, D. W. Wolan, K. B. Sharpless, J. E. Moses, *Angew. Chem. Int. Ed.* **2020**, *59*, 12460–12469. https://doi.org/10.1002/anie.202003219.

[277] W. H. Chiou, K. H. Hsu, W. W. Huang, *ACS Omega* **2020**, *5*, 3717–3724. https://doi.org/10.1021/acsomega.9b04400.

[278] J. L. Wu, W. H. Chiou, *J. Org. Chem.* **2020**, *85*, 9051–9063. https://doi.org/10.1021/acs.joc.0c00964.

[279] B. D. Ding, J. Sun, W. Jiang, G. Jin, C. G. Yan, *ChemistrySelect* **2020**, *5*, 1092–1096. https://doi.org/10.1002/slct.201904941.

[280] L. Wang, X. X. An, Y. F. Cui, W. K. Dong, *Zeitschrift fur Krist. New Cryst. Struct.* **2020**, *235*, 359–360. https://doi.org/10.1515/ncrs-2019-0670.

[281] P. Das, D. W. Almond, L. N. Tumbelty, B. E. Austin, G. Moura-Letts, *Org. Lett.* **2020**, *22*, 5491–5495. https://doi.org/10.1021/acs.orglett.0c01846.

[282] Y. L. Zhang, R. T. Guo, H. Luo, X. S. Liang, X. C. Wang, *Org. Lett.* **2020**, *22*, 5627–5632. https://doi.org/10.1021/acs.orglett.0c01992.

[283] Y. Moon, W. Lee, S. Hong, *J. Am. Chem. Soc.* **2020**, *142*, 12420–12429. https://doi.org/10.1021/jacs.0c05025.

[284] S. B. Duan, H. Y. Zhang, B. Y. Hao, J. Zhao, Y. P. Han, Y. Zhang, Y. M. Liang, *Org. Chem. Front.* **2021**, *8*, 5895–5901. https://doi.org/10.1039/d1qo00893e.

[285] E. A. Younes, M. J. Issa, M. F. Abdollahi, Y. F. Ding, A. J. Rasras, G. S. P. Mok, J. Bin Lin, Y. Zhao, *New J. Chem.* **2021**, *45*, 17366–17376. https://doi.org/10.1039/d1nj03044b.

[286] S. Liu, H. Qian, T. Zhang, H. Xie, Z. Han, W. Guo, H. Huang, J. Sun, *Angew. Chem. Int. Ed.* **2021**, *60*, 21272–21276. https://doi.org/10.1002/anie.202108258.

[287] W. Li, Y. Wang, H. Qi, R. Shi, J. Li, S. Chen, X. M. Xu, W. L. Wang, *Org. Biomol. Chem.* **2021**, *19*, 8086–8095. https://doi.org/10.1039/d1ob01206a.

[288] V. Kremláček, M. Hejda, E. Rychagova, S. Ketkov, R. Jambor, A. Růžička, L. Dostál, *Eur. J. Inorg. Chem.* **2021**, *2021*, 4030–4041. https://doi.org/10.1002/ejic.202100648.

[289] R. P. Singh, M. R. Bhandari, F. M. Torres, T. Doundoulakis, D. Gout, C. J. Lovely, *Org. Lett.* **2020**, *22*, 3412–3417. https://doi.org/10.1021/acs.orglett.0c00883.

[290] M. Taj Muhammad, Y. Jiao, C. Ye, M. F. Chiou, M. Israr, X. Zhu, Y. Li, Z. Wen, A. Studer, H. Bao, *Nat. Commun.* **2020**, *11*, 1–8. https://doi.org/10.1038/s41467-019-14254-3.

[291] M. Koy, P. Bellotti, F. Katzenburg, C. G. Daniliuc, F. Glorius, *Angew. Chem. Int. Ed.* **2020**, *59*, 2375–2379. https://doi.org/10.1002/anie.201911012.

[292] H. Ishikawa, K. Ban, N. Uemura, Y. Yoshida, T. Mino, Y. Kasashima, M. Sakamoto, *Eur. J. Org. Chem.* **2020**, *2020*, 1001–1005. https://doi.org/10.1002/ejoc.201901826.

[293] S. Jin, H. T. Dang, G. C. Haug, R. He, V. D. Nguyen, V. T. Nguyen, H. D. Arman, K. S. Schanze, O. V. Larionov, *J.*

Am. Chem. Soc. **2020**, *142*, 1603–1613. https://doi.org/10.1021/jacs.9b12519.

[294] Z. Zhou, R. K. Kawade, Z. Wei, F. Kuriakose, Ö. Üngör, M. Jo, M. Shatruk, R. Gershoni-Poranne, M. A. Petrukhina, I. V. Alabugin, *Angew. Chem. Int. Ed.* **2020**, *59*, 1256–1262. https://doi.org/10.1002/anie.201911319.

[295] Á. Acevedo-García, J. G. Alvarado-Rodríguez, N. Andrade-López, J. A. Álvarez-Hernández, *Inorg. Chem. Commun.* **2020**, *112*, 107750. https://doi.org/10.1016/j.inoche.2019.107750.

[296] B. Audic, N. Cramer, *Org. Lett.* **2020**, *22*, 5030–5034. https://doi.org/10.1021/acs.orglett.0c01606.

[297] Y. J. Wang, X. X. Li, Z. Chen, *J. Org. Chem.* **2020**, *85*, 7694–7703. https://doi.org/10.1021/acs.joc.0c00146.

[298] J. Lou, J. Ma, B. H. Xu, Y. G. Zhou, Z. Yu, *Org. Lett.* **2020**, *22*, 5202–5206. https://doi.org/10.1021/acs.orglett.0c01645.

[299] N. Zou, J. X. Lan, G. G. Yan, C. Liang, G. F. Su, D. L. Mo, *Org. Lett.* **2020**, *22*, 8446–8450. https://doi.org/10.1021/acs.orglett.0c02947.

[300] K. Norseeda, N. Chaisan, C. Thongsornkleeb, J. Tummatorn, S. Ruchirawat, *J. Org. Chem.* **2020**, *85*, 14271–14272. https://doi.org/10.1021/acs.joc.0c02380.

[301] I. Gospodinov, K. V. Domasevitch, C. C. Unger, T. M. Klapötke, J. Stierstorfer, *Cryst. Growth Des.* **2020**, *20*, 755–764. https://doi.org/10.1021/acs.cgd.9b01177.

[302] N. Mroweh, F. Pop, C. Mézière, M. Allain, P. Auban-Senzier, N. Vanthuyne, P. Alemany, E. Canadell, N. Avarvari, *Cryst. Growth Des.* **2020**, *20*, 2516–2526. https://doi.org/10.1021/acs.cgd.9b01665.

[303] R. Shankar, N. Mahavar, *Dalt. Trans.* **2020**, *49*, 16633–16637. https://doi.org/10.1039/d0dt03252b.

[304] T. C. Jankins, R. Martin-Montero, P. Cooper, R. Martin, K. M. Engle, *J. Am. Chem. Soc.* **2021**, *143*, 14981–14986. https://doi.org/10.1021/jacs.1c07162.

[305] T. I. Kostelnik, H. Scheiber, R. Cappai, N. Choudhary, F. Lindheimer, M. De Guadalupe Jaraquemada-Peláez, C. Orvig, *Inorg. Chem.* **2021**, *60*, 5343–5361. https://doi.org/10.1021/acs.inorgchem.1c00290.

[306] A. G. Lvov, M. Mörtel, F. W. Heinemann, M. M. Khusniyarov, *J. Mater. Chem. C* **2021**, *9*, 4757–4763. https://doi.org/10.1039/d1tc00761k.

[307] Z. Wang, G. Yue, X. Ji, H. Song, P. Yan, J. Zhao, X. Jia, *J. Org. Chem.* **2021**, *86*, 14131–14143. https://doi.org/10.1021/acs.joc.1c01586.

[308] R. J. Ross, R. Jeyaseelan, M. Lautens, *Org. Lett.* **2020**, *22*, 4838–4843. https://doi.org/10.1021/acs.orglett.0c01655.

[309] H. Z. Tejeneki, A. Nikbakht, S. Balalaie, F. Rominger, *J. Org. Chem.* **2020**, *85*, 8544–8552. https://doi.org/10.1021/acs.joc.0c00895.

[310] A. Schild, R. Bhardwaj, N. Wenger, D. Tscherrig, P. Kandasamy, J. Dernič, R. Baur, C. Peinelt, M. A. Hediger, M. Lochner, *Int. J. Mol. Sci.* **2020**, *21*, 1–28. https://doi.org/10.3390/ijms21165604.

[311] X. Buol, K. Robeyns, C. C. Garrido, N. Tumanov, L. Collard, J. Wouters, T. Leyssens, *Pharmaceutics* **2020**, *12*, 1–14. https://doi.org/10.3390/pharmaceutics12070653.

[312] S. Peil, G. Bistoni, R. Goddard, A. Fürstner, *J. Am. Chem. Soc.* **2020**, *142*, 18541–18553. https://doi.org/10.1021/jacs.0c07808.

[313] J. Bresien, Y. Pilopp, A. Schulz, L. S. Szych, A. Villinger, R. Wustrack, *Inorg. Chem.* **2020**, *59*, 13561–13571. https://doi.org/10.1021/acs.inorgchem.0c01934.

[314] B. T. Jones, J. García-Cárceles, L. Caiger, I. R. Hazelden, R. J. Lewis, T. Langer, J. F. Bower, *J. Am. Chem. Soc.* **2021**, *143*, 15593–15598. https://doi.org/10.1021/jacs.1c08615.

[315] X. Lei, Y. Li, Y. Lai, S. Hu, C. Qi, G. Wang, Y. Tang, *Angew. Chem. Int. Ed.* **2021**, *60*, 4221–4230. https://doi.org/10.1002/anie.202013169.

[316] T. Bartelmann, F. Gnannt, M. Zitzmann, P. Mayer, H. Dube, *Chem. Sci.* **2021**, *12*, 3651–3659. https://doi.org/10.1039/d0sc04981f.

[317] Y. J. Chen, F. G. Zhang, J. A. Ma, *Org. Lett.* **2021**, *23*, 6062–6066. https://doi.org/10.1021/acs.orglett.1c02139.
